# Transcriptome-Guided Identification of Two GPCR Candidates Contributing to Trap Formation in *Arthrobotrys oligospora*

**DOI:** 10.3390/ijms27219599

**Published:** 2026-10-27

**Authors:** Junjie Liu, Yunlong Li, Fang Zhan, Mengsha Deng, Yuhua Wang, Xin Wang

**Affiliations:** 1 Key Laboratory for Conservation and Utilization of Bio-Resources in Yunnan, Yunnan University, Kunming 650091, China; liujunjie1@stu.ynu.edu.cn (J.L.);; 2 Yunnan Key Laboratory of Basic Research and Innovative Application for Green Biological Production, Yunnan University, Kunming 650091, China; 3 Southwest United Graduate School, Kunming 650092, China

**Keywords:** *Arthrobotrys oligospora*, G protein-coupled receptor, nematode-trapping fungus, trap morphogenesis, Gα response, BRET, multi-transcriptome analysis

## Abstract

G protein-coupled receptors (GPCRs) connect environmental cues to fungal development, but the functions of many candidate receptors in nematode-trapping fungi remain unresolved. We combined reference-genome mining, public transcriptome analysis and functional assays to investigate GPCR candidates associated with trap formation in *Arthrobotrys oligospora* ATCC 24927. Genome mining yielded 87 candidate receptors, including two provisional entries. Transcriptome analysis across nine retained Gene Expression Omnibus (GEO) series supported an exploratory shortlist of three candidates, designated AoGrn1, AoGrn2 and AoGrn3. Deletion of *AoGrn1* or *AoGrn2* reduced nematode-induced trap formation, and complementation partially restored the phenotype. *AoGrn3* deletion produced no statistically detectable change in trap density under the tested conditions. In HEK293F cells, nematode extract elicited bioluminescence resonance energy transfer responses in the AoGrn1–AoGpa2 and AoGrn2–AoGpa1 combinations. AlphaFold2-Multimer models suggested intracellular receptor–Gα interfaces, although model score preferences did not consistently match the observed response profiles. These findings support roles for AoGrn1 and AoGrn2 in trap formation and identify receptor–Gα combinations for further investigation of prey-associated signaling. The active extract components and the corresponding coupling relationships in the native fungus remain to be established.

## 1. Introduction

Fungi adapt their growth and development to changing environmental conditions. In pathogenic and predatory species, environmental sensing initiates the formation of specialized infection or capture structures. G protein-coupled receptors (GPCRs) connect extracellular cues to heterotrimeric G proteins and downstream signaling networks that regulate fungal development, stress adaptation and host interactions [1]. Fungal genomes contain expanded receptor families, including the fungus-specific Pth11-like proteins first systematically described in the rice blast fungus [2]. In *Fusarium graminearum*, a genome-wide analysis identified 105 putative GPCRs and linked several infection-induced receptors to infection-cushion formation, invasive growth and wheat infection. One receptor, GIV1, connects host-derived signals to cyclic AMP-dependent protein kinase A (cAMP–PKA) and the Gpmk1 mitogen-activated protein kinase (MAPK) pathway [3]. Defining ligand recognition and G-protein engagement across these receptor families is central to understanding environmentally induced fungal morphogenesis.

Nematode-trapping fungi offer a tractable system for investigating how extracellular cues trigger predatory differentiation. Under nutrient limitation, *Arthrobotrys oligospora* forms adhesive networks that capture and digest nematodes [4,5]. Nematode-derived ascaroside pheromones induce trap formation, establishing prey-associated chemicals as developmental signals [6]. In *A. oligospora*, transcriptomic and genetic analyses identified Gpr2, Gpr3 and Gin3 as components of prey sensing. Gpr2 and Gpr3 contribute to ascaroside responses through AoGpa2, whereas Gin3 participates in sensing additional nematode cues; these receptors engage cAMP–PKA signaling and modulate MAPK activity [7]. In *Arthrobotrys flagrans*, GprC localizes to the plasma membrane and outer mitochondrial membrane, couples to GasA, and coordinates transcriptional reprogramming with mitochondrial respiration during trap development [8]. These studies reveal multiple receptor-dependent routes from prey detection to fungal differentiation.

Several downstream signaling modules coordinate trap morphogenesis in *A. oligospora*. The genome and proteome of strain ATCC 24927 established a foundation for analyzing the transition to predation [9]. The Gβ subunit Gpb1, the Fus3 pheromone-response MAPK cascade and its downstream transcription factor Ste12 are required for normal nematode-induced trap development [10,11,12]. AoGpa2 activates adenylyl cyclase and cAMP–PKA signaling, while Ric8 and regulators of G-protein signaling modulate development, metabolism and pathogenicity [13,14,15,16]. The high-osmolarity glycerol and cell-wall-integrity MAPK pathways also contribute to trap differentiation and stress adaptation [17,18,19,20]. Time-resolved transcriptomics has resolved distinct programs associated with prey sensing, trap construction and digestion [21]. Connecting these downstream programs to individual receptors requires broader functional characterization of the GPCR repertoire and its Gα response preferences.

Here, we investigated which uncharacterized GPCR candidates contribute to nematode-induced trap formation in *A. oligospora* ATCC 24927. We first defined a reference-genome receptor inventory and used public transcriptome data to prioritize candidates responsive to nematode exposure and signaling-pathway perturbations. Three candidates, designated AoGrn1, AoGrn2 and AoGrn3, were selected for deletion analysis, with complementation used to assess the trap phenotypes associated with AoGrn1 and AoGrn2. We further examined nematode-extract-dependent Gα response profiles in HEK293F cells and used AlphaFold2-Multimer models to explore possible receptor–Gα interfaces. Together, these analyses assess candidate receptor contributions to predatory development and generate hypotheses about their proximal signaling partners.

## 2. Results

### 2.1. Reference-Genome Mining Defines 87 Candidate GPCRs and Their Predicted Gα Interfaces

**Figure 1:**
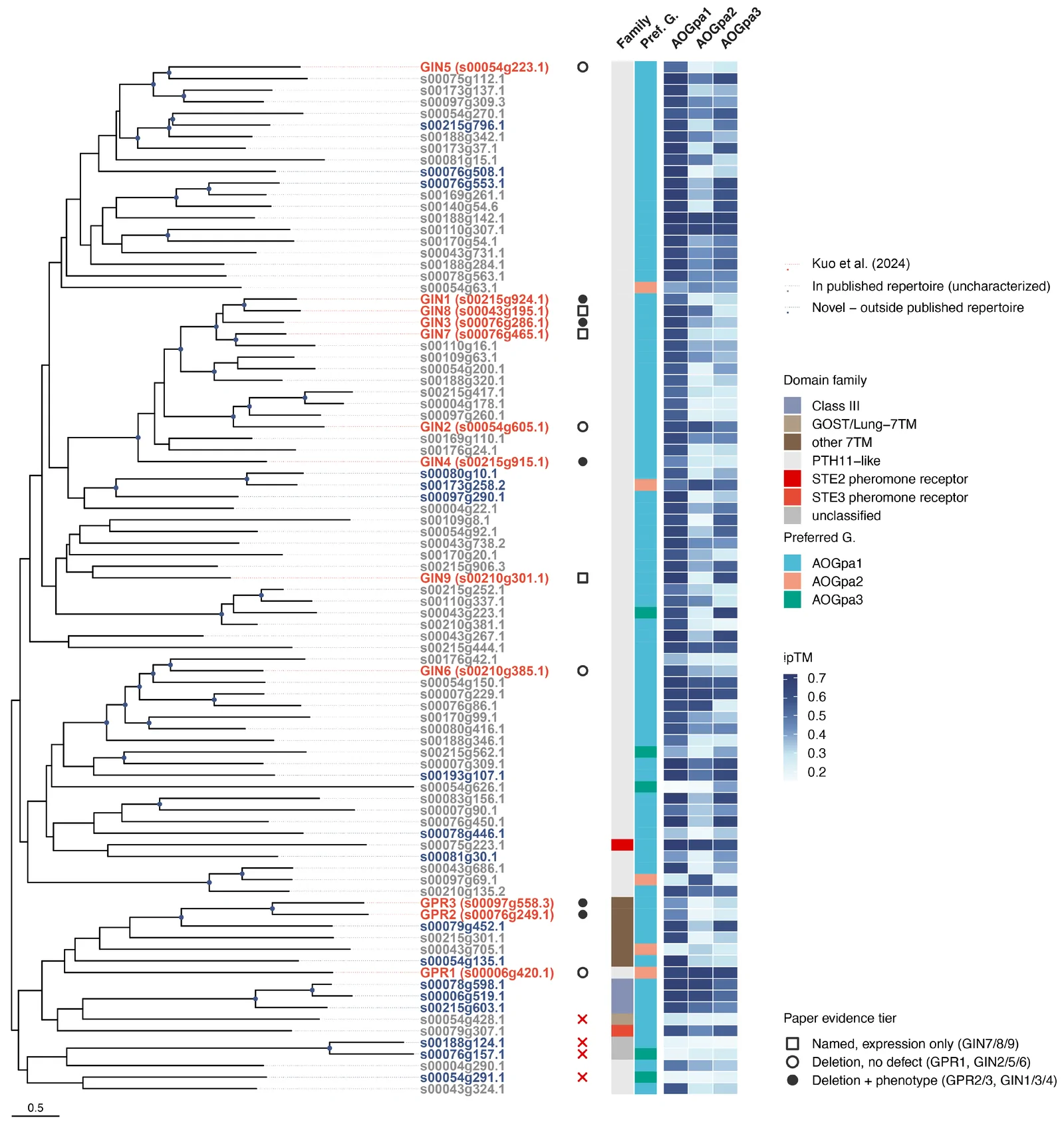
Reference-genome GPCR repertoire and predicted GPCR–Gα interaction landscape in *A. oligospora* ATCC 24927. A midpoint-rooted maximum-likelihood tree was inferred from 89 full-length receptor sequences; blue internal-node dots indicate ultrafast bootstrap support ≥ 95, and the scale bar denotes substitutions per site. Tip-label color indicates receptors named/studied in the published repertoire [7], receptors present but uncharacterized in that repertoire, and candidates outside it. Annotation strips show domain family, the highest-scoring Gα partner and ipTM values for AoGpa1, AoGpa2 and AoGpa3. Symbols summarize prior expression/deletion evidence. Red crosses identify four candidates lacking predicted contacts with all three Gα subunits. Gα ranking is interpreted within each receptor; absolute ipTM values are not compared across receptors.

Re-annotation of the *A. oligospora* ATCC 24927 reference genome yielded an inventory of 89 candidate GPCRs for subsequent analyses (Figure 1, Table S1). The current topology annotations indicated seven transmembrane segments in 84 candidates and six in five candidates. Two six-transmembrane candidates, AOL_s00076g157.1 and AOL_s00188g124.1, had only generic transmembrane-protein annotations and were retained as provisional entries. Most candidates carried Pth11-like annotations, with smaller groups assigned to Class III, GOST/Lung-7TM, STE2, STE3 and other GPCR-associated categories. Comparison with the published receptor repertoire identified 17 additional candidates distributed across several phylogenetic groups [7].

We modeled each candidate with the three *A. oligospora* Gα subunits, generating 267 receptor–Gα combinations and 801 scored models across three model weights. Gα partners were ranked within each candidate receptor using the interface-predicted TM-score (ipTM). Using the highest ipTM for each receptor–Gα pair, AoGpa1 was the highest-scoring partner for 79 of 89 candidates. AoGpa1 scores exceeded those of AoGpa2 for 81 candidates and those of AoGpa3 for 79 candidates. No interchain contacts were detected under the applied contact criterion in seven AoGpa1, 22 AoGpa2 and 11 AoGpa3 models selected for interface analysis.

We assessed the stability of partner rankings by comparing alternative model score summaries. AoGpa1 was the highest-scoring partner for 80 of 89 candidates when scores were averaged across the three model weights. The highest-scoring partner was unchanged between maximum- and mean-score summaries for 84 candidates and was consistent across all three matched model weights for 69 candidates. These analyses revealed a predominant AoGpa1 model score preference within the candidate inventory, without establishing functional coupling selectivity. The predicted complexes provided candidate interfaces for subsequent analysis of the experimentally selected receptors.

Closer inspection of the domain annotations identified two entries, AOL_s00076g157.1 and AOL_s00188g124.1, annotated only as transmembrane proteins. Both contained just six predicted transmembrane segments, falling short of the seven-transmembrane topology expected of a GPCR, and were therefore not supported as GPCR candidates. We retained them in the initial inventory and structural analysis but excluded them from subsequent transcriptome-based prioritization. The remaining 87 candidates were used in all subsequent analyses.

### 2.2. Integrated Multi-Transcriptome Analysis Prioritizes Three Uncharacterized GPCRs

**Figure 2:**
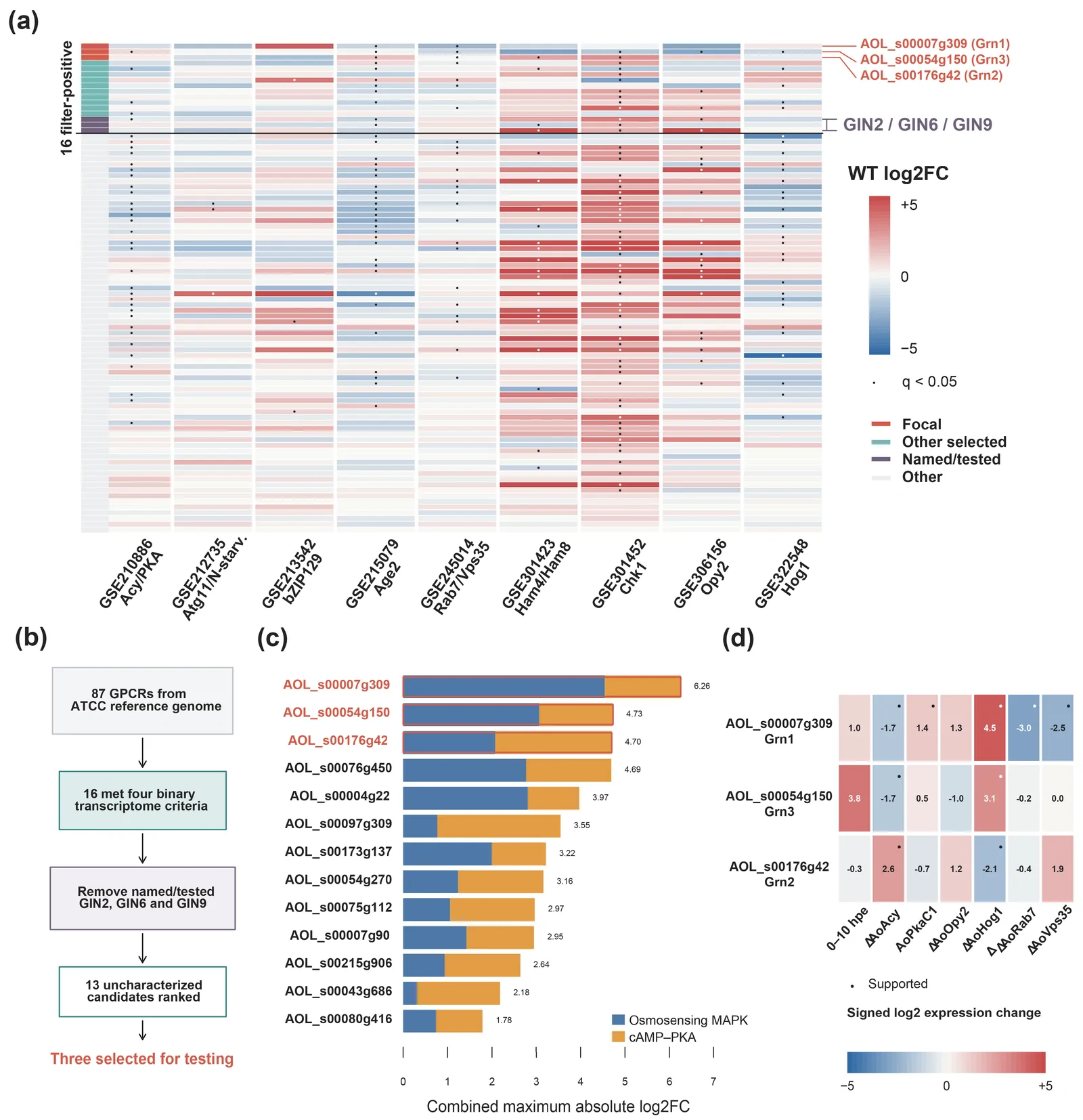
Multi-transcriptome analysis prioritizes three candidate GPCRs for functional testing. (**a**), WT transcriptional responses of the 87 candidate GPCRs across nine GEO series after excluding GSE253759 and GSE303416. Each cell represents the median within-series log2 fold change relative to the corresponding baseline. Red and blue indicate increased and decreased transcript abundance, respectively; gray cells indicate unmapped receptors. Row annotations distinguish the three focal candidates, other filter-positive candidates, previously named or tested receptors, and remaining candidates. The 16 filter-positive receptors are grouped at the top. Dots indicate at least one underlying contrast meeting the support criteria of Benjamini–Hochberg-adjusted q < 0.05 and |log2 fold change| ≥ 1. (**b**), Candidate-selection workflow. Four criteria—membership in the reference GPCR inventory, transcriptional responsiveness to nematode exposure, and supported responses in osmosensing MAPK and cAMP–PKA perturbation datasets—identified 16 candidates. Exclusion of the previously characterized GIN2, GIN6 and GIN9 left 13 candidates for ranking. Arrows indicate the direction of the workflow. (**c**), Candidates ranked by the sum of their maximum absolute log2 fold changes in the osmosensing MAPK (blue) and cAMP–PKA (orange) modules. Red outlines identify the three candidates selected for experimental analysis: 200 (AoGrn1; *AOL_s00007g309*), 5K6 (AoGrn3; *AOL_s00054g150*) and PR8 (AoGrn2; *AOL_s00176g42*). This ranking provided an exploratory shortlist. (**d**), Signed transcriptional responses of the three focal candidates. The first column shows ortholog-mapped WT responses between 0 and 10 h after nematode exposure in the published TWF154 dataset. The remaining columns show median log2 fold changes in the indicated mutant backgrounds relative to their corresponding WT controls. Numbers indicate expression changes, and dots identify at least one supported underlying contrast.

We mapped the 87 candidates onto a cross-GEO expression resource comprising nine retained series with comparable wild-type (WT) induction contrasts, yielding 783 receptor–series estimates (Figure 2a). For each receptor, responses within each series were summarized as the median log_2_ fold change across the available processed expression matrices. These matrices contained transcripts-per-million (TPM) or fragments-per-kilobase-per-million (FPKM) values; raw-count data were modeled separately. Of the mapped receptors, 78 showed a Benjamini–Hochberg-adjusted q < 0.05 response in at least one series and 47 had support in at least three independent series. GSE253759 was excluded from independent-support counts because of its similarity to GSE215079. Response directions varied among studies, indicating broad, context-dependent regulation of the receptor repertoire.

To identify key receptors for further investigation, we prioritized candidates using four criteria: membership in the 87-GPCR inventory, responsiveness to nematode exposure, support in osmosensing MAPK perturbation datasets, and support in cAMP–PKA perturbation datasets. Sixteen receptors met all four criteria (Figure 2b). Excluding the previously named or experimentally examined GIN2, GIN6 and GIN9 left 13 uncharacterized candidates for ranking.

Candidates were ranked by the sum of their maximum absolute log2 fold changes in the osmosensing MAPK and cAMP–PKA datasets. AOL_s00007g309, AOL_s00054g150 and AOL_s00176g42 ranked highest, with scores of 6.260, 4.731 and 4.702, respectively (Figure 2c). The fourth-ranked receptor, AOL_s00076g450, scored 4.692. We advanced the three highest-ranked candidates for experimental analysis, with AOL_s00007g309 showing the clearest numerical separation.

The selected receptors displayed distinct response profiles (Figure 2d). Between baseline and 10 hpe, AOL_s00007g309 increased by 0.952 log2 units (13.37 to 25.95 TPM), and AOL_s00054g150 increased by 3.769 log2 units from a low baseline (0.02 to 1.55 TPM). AOL_s00176g42 decreased by 0.323 log2 units (16.95 to 13.53 TPM). Across signaling perturbations, AOL_s00007g309 responded most strongly in Δ*AoHog1* (+4.545 log2 fold change), with additional supported changes in Δ*AoAcy* (−1.715) and Δ*AoPkaC1* (+1.374). AOL_s00054g150 showed its largest changes in Δ*AoHog1* (+3.072) and Δ*AoAcy* (−1.659), whereas AOL_s00176g42 changed in opposite directions in Δ*AoAcy* (+2.630) and Δ*AoHog1* (−2.072). Thus, nematode responsiveness and transcriptional sensitivity to signaling perturbation jointly supported the selection of these candidates.

### 2.3. AoGrn1 and AoGrn2 Promote Trap Formation

**Figure 3:**
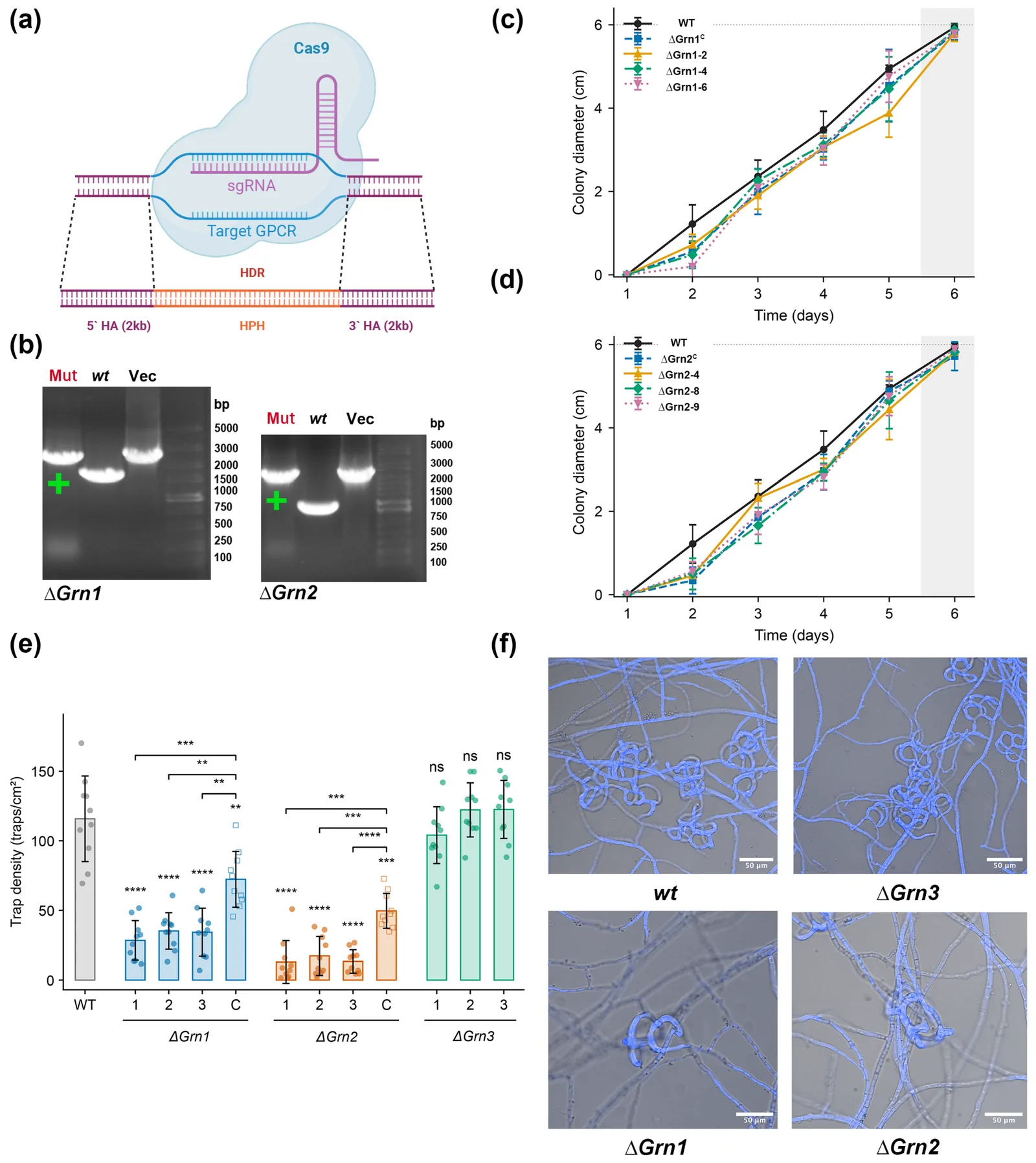
Independent deletion and complementation analyses support roles for AoGrn1 and AoGrn2 in trap formation in *A. oligospora*. (**a**), Schematic of CRISPR/Cas9-mediated replacement of the target gene with an HPH resistance cassette flanked by approximately 2 kb homology arms. HDR, homology-directed repair; HA, homology arm. Dashed lines indicate the regions of homologous recombination. (**b**), Representative diagnostic PCR products for the *AoGrn1* and *AoGrn2* loci. Mut, deletion strain; wt, wild type; Vec, targeting vector. DNA fragment sizes are indicated in base pairs. The green + indicates the expected diagnostic PCR band for the mutant strains. (**c**,**d**), Colony-diameter trajectories of WT, three independent deletion isolates and the corresponding complemented strain for AoGrn1 (c) and AoGrn2 (**d**). Data represent mean ± SD from five independent culture plates per strain, with the same plates measured daily. Gray shading indicates day 6, when colony expansion was limited by the plate edge. The same WT reference is shown in both panels. (**e**), Trap densities measured 30 h after addition of approximately 200 L4-stage *Caenorhabditis elegans*. Three independent deletion isolates (1–3) were examined for each gene; C denotes the corresponding complemented strain. Bars represent mean ± SD, and individual points represent independent culture plates (*n* = 10 per strain). Dashed lines with open squares identify complemented strains. Traps were counted within two randomly selected regions covering a combined area of 4.5 cm^2^ per plate, and the total was divided by 4.5 to give traps cm^−2^. All induction assays were performed in one experimental batch. Symbols above individual bars indicate comparisons with WT; brackets indicate comparisons between complemented strains and the corresponding deletion isolates. Pairwise comparisons used two-sided, unpaired Welch *t*-tests with Holm correction across 17 comparisons: 11 non-WT-versus-WT comparisons and six complemented-strain-versus-deletion-isolate comparisons. ** *p* < 0.01, *** *p* < 0.001, **** *p* < 0.0001; ns, adjusted *p* ≥ 0.05. (**f**), Representative micrographs of traps formed by WT and the indicated deletion strains. Scale bars, 50 μm.

For clarity and consistency with existing literature, the candidate GPCRs in *A. oligospora* were systematically named AoGrnX (GPCR related to nematodes), followed by a sequential number. The three prioritized loci were assigned aliases based on the final three characters of their UniProt accessions: AoGrn1, AOL_s00007g309/G1X200; AoGrn3, AOL_s00054g150/G1X5K6; and AoGrn2, AOL_s00176g42/G1XPR8 (Supplementary Table S2). Targeted replacement constructs yielded diagnostic PCR products consistent with replacement of each locus (Figure 3a,b). Complemented strains were generated for Δ*AoGrn1* and Δ*AoGrn2*. For AoGrn1 and AoGrn2, WT, deletion and complemented strains showed visually similar growth trajectories over five days (Figure 3c).

Relative transcript abundance was measured in three biological replicates per genotype and time point, each assayed in triplicate reactions and normalized to Tub. Target transcripts were undetected in Δ*AoGrn1*, Δ*AoGrn2* and Δ*AoGrn3* at 0 and 10 h (0/9 reactions per target–genotype–time combination), while Tub was detected in all samples (Supplementary Figure S6, Table S7 and Data S2). In the wild type, 10 h/0 h ratios were 2.57, 1.72 and 9.76 for AoGrn1, AoGrn2 and AoGrn3, respectively (geometric means of three paired replicates); none reached significance after Holm correction across the 27 planned comparisons (*p* = 0.310, 0.797 and 0.092), and these trends are therefore reported descriptively.

At 30 h after induction with nematode extract, WT cultures produced 115.8 ± 30.8 traps cm^−2^ (mean ± s.d., *n* = 10 plates per isolate). All three Δ*AoGrn1* isolates formed fewer traps, with isolate means of 28.4–35.2 traps cm^−2^ (69.6–75.5% below WT), and the three Δ*AoGrn2* isolates were more strongly affected, at 12.9–17.2 traps cm^−2^ (85.1–88.9% below WT); all six Δ*AoGrn1* and Δ*AoGrn2* isolates differed significantly from WT (Holm-adjusted *p* < 0.0001; Figure 3e). By contrast, the three Δ*AoGrn3* isolates produced 104.1–122.5 traps cm^−2^, indistinguishable from WT (all adjusted *p* ≥ 0.988). Representative micrographs were consistent with these quantifications in which WT and Δ*AoGrn3* mycelia formed numerous adhesive networks, whereas Δ*AoGrn1* and Δ*AoGrn2* formed markedly fewer traps (Figure 3f). Complementation restored trap density to 72.3 ± 20.1 traps cm^−2^ for AoGrn1 and 49.6 ± 12.5 traps cm^−2^ for AoGrn2 (mean ± s.d., *n* = 10 plates per strain); both complemented strains exceeded each of their three corresponding deletion isolates (all adjusted *p* ≤ 0.0013) yet remained below WT (adjusted *p* = 0.0074 and 0.00031, respectively), indicating partial restoration of trap formation and supporting the involvement of both AoGrn1 and AoGrn2 in this process.

### 2.4. AoGrn1 and AoGrn2 Show Distinct Gα Responses in HEK293F Cells

**Figure 4:**
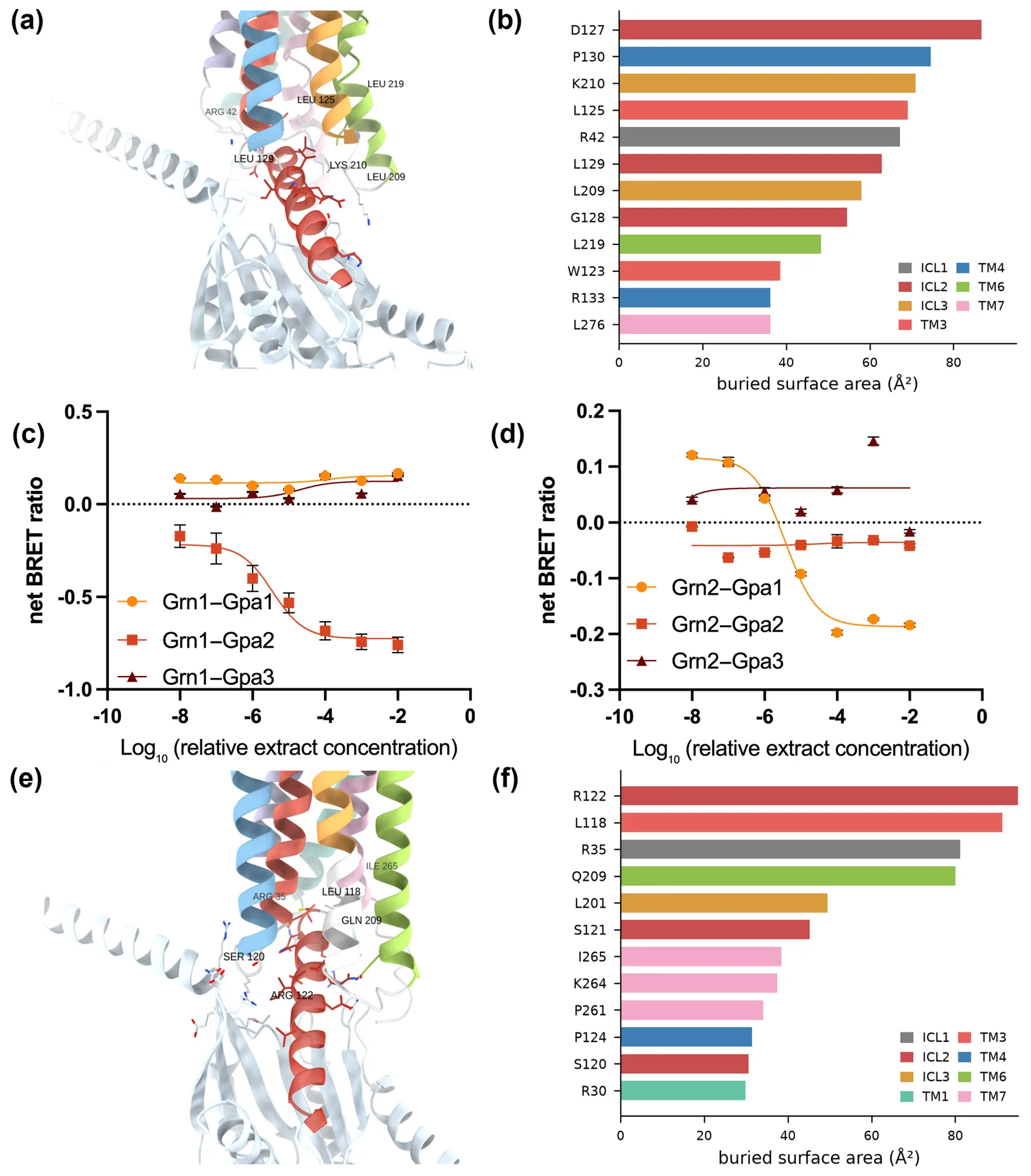
Nematode-extract-dependent BRET responses and predicted receptor–Gα interfaces of AoGrn1 and AoGrn2. (**a**,**b**) Predicted AoGrn1–AoGpa2 interface and receptor-side buried surface area per residue. (**c**,**d**) BRET responses across serial dilutions of nematode extract for AoGrn1 (**c**) and AoGrn2 (**d**), each paired with AoGpa1, AoGpa2 or AoGpa3. Under the tested HEK293F biosensor configuration, concentration-dependent decreases in net BRET were observed for AoGrn1–AoGpa2 and AoGrn2–AoGpa1. The horizontal dashed line at 0.0 in (**c**,**d**) indicates the baseline net BRET ratio. (**e**,**f**) Predicted AoGrn2–AoGpa1 interface and receptor-side buried surface area per residue. Receptor transmembrane helices and intracellular loops are color-coded as indicated. The Gα subunit is shown in pale blue, with its α5 helix highlighted in red.

**Figure 5:**
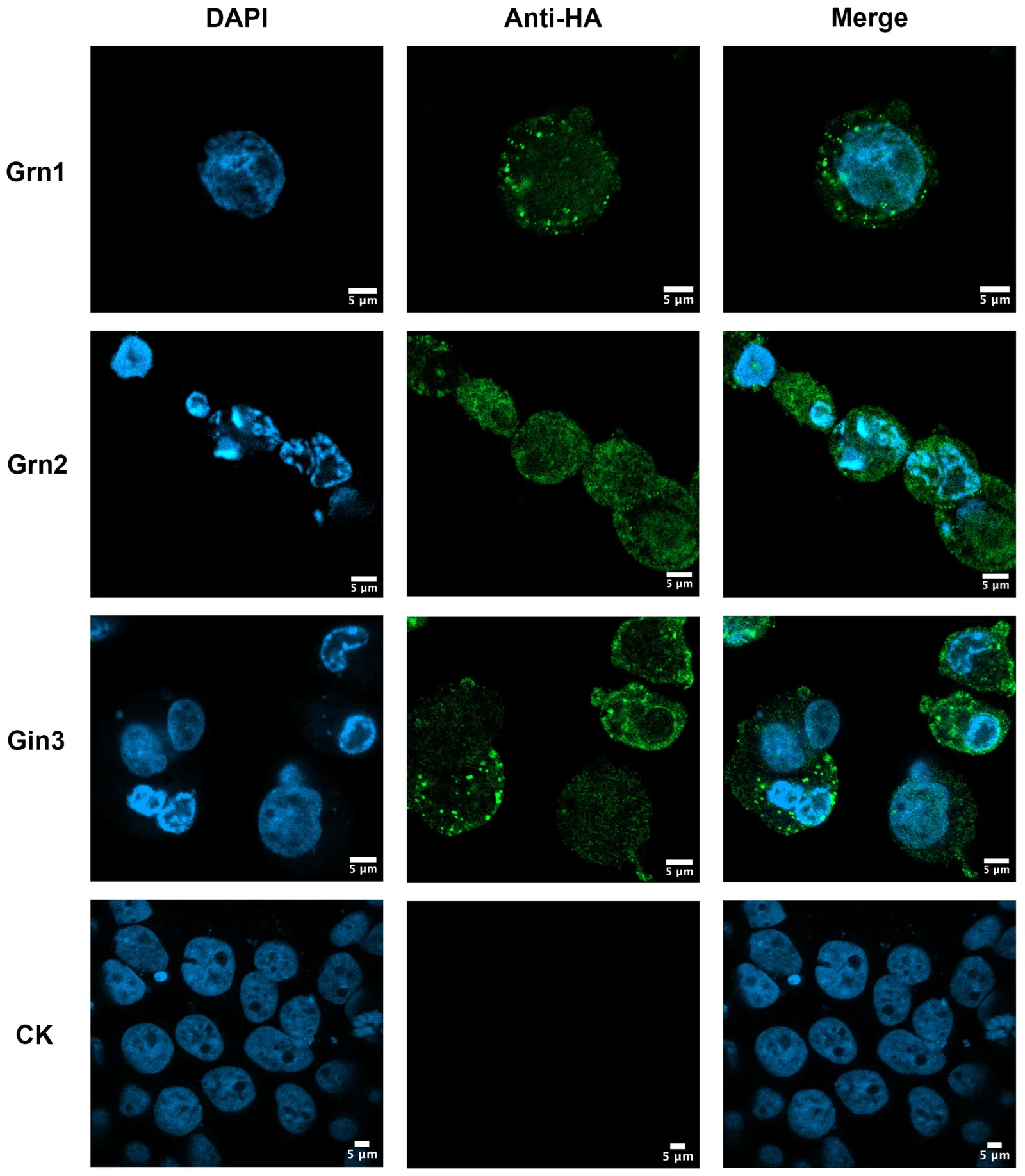
Subcellular localization of HA-tagged AoGrn1, AoGrn2, Gin3 and Empty Vector. Representative confocal immunofluorescence images of cells expressing HA-tagged AoGrn1, AoGrn2, Gin3 (positive control), and Empty Vector (EV, negative control). Cells were fixed and immunostained with a rabbit anti-HA primary antibody followed by a CoraLite488-conjugated secondary antibody (green) to visualize receptor localization. Cell nuclei were counterstained with DAPI (blue). Merged images (Merge) show the total (permeabilized) receptor pool, with both membrane-associated and intracellular signal. Images were acquired on a Zeiss LSM 980 confocal microscope. Scale bars, 5 μm.

We examined AoGrn1 and AoGrn2 with each of the three Gα subunits using a BRET biosensor in HEK293F cells. Across the nematode-extract (NE) dilution series, decreases in net BRET were observed for AoGrn1–AoGpa2 and AoGrn2–AoGpa1, whereas the other receptor–Gα combinations showed comparatively flat profiles (Figure 4c,d). Gin3 positive-control and receptor-free sensor measurements are provided in Supplementary Figure S7. These observations identify two receptor–Gα combinations with NE-associated BRET changes under the tested heterologous conditions. The decreases are consistent with changes in the relative arrangement of the tagged G-protein subunits, but do not establish exclusive coupling to these partners. Immunofluorescence analysis detected robust expression of HA-Grn1, HA-Grn2, and the positive control HA-Gin3 in transfected 293F cells, with no background fluorescence observed in empty vector (EV) controls. Both HA-Grn1 and HA-Grn2 exhibited subcellular distribution patterns comparable to HA-Gin3, characterized by peripheral membrane-associated fluorescence (prominent puncta in Grn1 and cell-boundary outlining in Grn2) alongside intracellular perinuclear and vesicular staining (Figure 5).

Selected models of the two combinations exhibiting BRET decreases placed the C-terminal Gα α5 helix within the intracellular receptor cavity (Figure 4a,e). In the AoGrn1–AoGpa2 model, D127 in intracellular loop 2 (ICL2), P130 in transmembrane helix 4 (TM4) and K210 in ICL3 made the largest receptor-side contributions to buried surface area (86.7, 74.6 and 70.9 Å^2^, respectively; Figure 4b). In the AoGrn2–AoGpa1 model, the largest contributions were from R122 in ICL2, L118 in TM3 and R35 in ICL1 (95.2, 91.3 and 81.2 Å^2^, respectively; Figure 4f). These model-derived contacts provide candidate sites for mutational testing.

The two receptors did not show the same relationship between the structural predictions and the BRET profiles, and we therefore treated the modeled interfaces as hypotheses rather than as evidence for coupling. ipTM values were low in absolute terms (0.29–0.37 across the evaluated models and seeds for AoGrn2–AoGpa1) and were used only to rank Gα partners within a receptor, as stated in the Figure 4 legend. For AoGrn2, the number of receptor-side residue pairs meeting the predefined contact criteria decreased in the order AoGpa1 (65) > AoGpa2 (13) > AoGpa3 (5), which matched the combination showing a net BRET decrease. For AoGrn1, the three interfaces were comparably populated (58–64 pairs) and AoGpa1 received the highest ipTM scores, yet the BRET decrease was restricted to AoGpa2; for this receptor, the models do not discriminate among partners. In both cases, the contact residues in Figure 4b,f identify candidate positions for mutational testing rather than an assignment of coupling specificity.

## 3. Discussion

AoGrn1 and AoGrn2 contribute to nematode-induced trap formation in *A. oligospora*. Deletion of either gene reduced trap production, and complementation partially restored the phenotype. In HEK293F cells, nematode extract was associated with decreases in net BRET in the AoGrn1–AoGpa2 and AoGrn2–AoGpa1 combinations. These findings associate two candidate receptors with predatory development and reveal distinct Gα response profiles under the tested heterologous conditions. Together, the genetic and signaling analyses provide a basis for investigating how these receptors contribute to the transition from vegetative growth to trap formation.

Previous research established that nematode-derived ascarosides induce trap formation and that *A. oligospora* uses multiple receptors to respond to prey-associated signals [6,7]. Gpr2 and Gpr3 contribute to ascaroside sensing, whereas Gin3 participates in responses to additional nematode-derived cues [7]. In *A. flagrans*, GprC and GasA coordinate transcriptional reprogramming and mitochondrial respiration during predatory development [8]. The AoGrn1 and AoGrn2 phenotypes extend this framework by identifying further candidate receptors associated with trap development. Their responses to crude extract do not establish whether they detect ascarosides, other prey-derived molecules or overlapping mixtures of stimuli. Fus3 MAPK, Gpa2-dependent cAMP–PKA signaling and the HOG pathway provide established routes through which environmental information can influence trap morphogenesis [11,13,17]. The transcriptional responses used here connect receptor expression to perturbations of these pathways, while the positions of AoGrn1 and AoGrn2 within them remain to be determined.

The association between receptor function and specialized morphogenesis also has precedents in plant-pathogenic fungi. Pth11 contributes to surface-cue-dependent appressorium differentiation in the rice blast fungus, and structure–function analysis links its CFEM domain to proper appressorium development [22,23]. In *F. graminearum*, an expanded GPCR subfamily contributes to infection-related development [3]. These comparisons support investigating receptor-dependent developmental transitions in A. oligospora, but they do not establish shared ligands or an equivalent CFEM-dependent mechanism for AoGrn1 and AoGrn2.

The deletion and partial-complementation phenotypes provide the principal evidence linking AoGrn1 and AoGrn2 to trap development. Partial restoration supports a gene-associated contribution, although differences in transgene expression or insertion context could influence the extent of complementation. Trap formation involves coordinated signaling and cellular differentiation [24]; disruption of cell-fusion components or cAMP phosphodiesterase activity can affect both colony growth and trapping phenotypes [25,26]. Basal growth therefore requires consideration when interpreting these phenotypes. Mean colony-growth AUCs were lower in the non-WT strains, but individual comparisons with WT did not reach significance after Holm correction. These measurements describe colony expansion on nutrient-rich medium and leave open a possible contribution from growth differences under trap-inducing conditions. AoGrn3 deletion produced no statistically detectable trap-number phenotype under the conditions tested. Its contrasting phenotype illustrates that transcriptional prioritization identifies candidates without necessarily predicting a detectable single-gene deletion effect. Redundancy and condition-dependent functions remain hypotheses for further testing.

The expression measurements further distinguish transcriptional responsiveness from functional contribution to trap formation. WT transcript-abundance estimates were higher at 10 h after nematode exposure than at baseline, but none of the three time comparisons reached significance after Holm adjustment across 27 comparisons. AoGrn3 showed the largest estimated increase, despite the absence of a detectable trap-number phenotype in its deletion strain. Thus, expression-change magnitude alone provides limited guidance about the effect of deleting a candidate receptor. Time-resolved transcriptomics has identified distinct programs associated with prey sensing, trap development and digestion [21], providing a rationale for evaluating receptor expression across matched developmental stages. Corresponding target transcripts were not detected in the deletion strains under the assay conditions. For AoGrn2, the direction of the post-exposure estimate differed from the modest decrease in an earlier public transcriptomic interval. The qPCR estimates assume comparable target and reference amplification efficiencies; the higher efficiency estimated for Tub limits their quantitative interpretation. Sampling at matched time points, together with time-matched unexposed controls, would help resolve the temporal expression pattern.

Comparison of structural predictions with BRET measurements revealed receptor-specific agreement between model scores and experimental response profiles. AoGrn1 had its highest model scores with AoGpa1, whereas its detectable response occurred with AoGpa2. For AoGrn2, both the highest-scoring partner and the observed response involved AoGpa1, although the absolute ipTM scores remained low. Benchmarking of transient protein complexes has shown that AlphaFold prediction accuracy varies across interaction classes and that interface confidence measures help evaluate the models [27]. This provides methodological context rather than a validation of the fungal receptor–Gα complexes. Here, agreement in partner ranking therefore does not validate the predicted interface or establish coupling selectivity. Evolutionary analysis has identified candidate determinants of human GPCR–G-protein recognition [28], and systematic experimental profiling has mapped receptor sequence features associated with coupling [29]. These studies provide a rationale for mutational testing of the fungal receptors; their specificity rules have not been validated for AoGrn1 or AoGrn2. BRET additionally depends on the proximity and relative orientation of its donor and acceptor [30], so its response is not equivalent to a static structural score. The predicted intracellular contacts provide candidate positions for testing their contributions to signaling while controlling for receptor expression and localization.

The heterologous assays provide a controlled setting for comparing candidate receptor–Gα combinations. Immunofluorescence documented an HA-associated signal in the heterologous expression system. Because the cells were permeabilized, these images do not distinguish surface-localized from intracellular receptor pools. Optimization of G-protein BRET sensors has shown that reporter placement and subunit configuration influence assay performance [30]; consequently, a flat profile in the present configuration does not establish an absolute inability to engage a particular Gα subunit. Native cellular context also warrants investigation: GprC has plasma-membrane and mitochondrial functions in *A. flagrans* [8], while methionine triggers Gpr4-dependent cAMP signaling and receptor internalization in *Cryptococcus neoformans* [31]. These examples motivate examining AoGrn1 and AoGrn2 localization and trafficking in the fungus. Furthermore, the active components of the nematode extract remain unidentified. Fractionation followed by testing of defined compounds, together with native Gα-dependence experiments, would help connect the heterologous responses to endogenous trap development.

The initial inventory comprised 89 proteins, of which 87 were retained for transcriptome-based prioritization after exclusion of two entries without GPCR-domain support. This retained set broadens the basis for receptor investigation in *A. oligospora*. Expanded fungal receptor families have been described in Magnaporthe and *F. graminearum* [2,3], but inventory size alone does not establish functional diversification. Comparison with the published TWF154 repertoire must account for strain background, gene annotation and candidate-identification criteria [7,9,10]. Candidates lacking a mapped counterpart therefore require sequence and functional validation before being considered strain-specific receptors. Cross-study transcriptional responses guided experimental prioritization, while structural modeling provided separate hypotheses about receptor–Gα interfaces. Excluding the duplicate-like and provisionally annotated GEO series preserved candidate eligibility, whereas alternative scoring schemes changed the order of closely ranked candidates. These results support treating the ranking as an exploratory shortlist. Functional analysis then distinguished the trap-associated phenotypes of AoGrn1 and AoGrn2 from the absence of a detectable trap-number phenotype for AoGrn3.

Together, the genetic phenotypes and heterologous response profiles identify AoGrn1 and AoGrn2 as candidates for studying receptor-mediated control of predatory development. Establishing their native signaling partners and defining the active nematode-derived components should clarify how these receptors contribute to the construction of fungal trapping networks.

## 4. Materials and Methods

### 4.1. Fungal Strains, Culture Conditions and Nematode Material

*A. oligospora* ATCC 24927 (American Type Culture Collection, Manassas, VA, USA) was the WT background for the deletion and complemented strains (Table S3). Cultures were maintained on potato dextrose agar (PDA; 200 g/L potato infusion, 20 g/L dextrose and 15 g/L agar) at 28 °C. Water agar (1.5% *w*/*v* agar in distilled water) was used for nutrient-deprived assays. *C. elegans* N2 was maintained at 20 °C on nematode growth medium (3 g/L NaCl, 2.5 g/L peptone and 17 g/L agar, supplemented with 1 mM CaCl_2_, 1 mM MgSO_4_, 5 µg/mL cholesterol and 25 mM potassium phosphate buffer, pH 6.0), with *Escherichia coli* OP50 as food [32]. OP50 was cultured in Luria–Bertani medium (10 g/L tryptone, 5 g/L yeast extract and 10 g/L NaCl).

### 4.2. Reference-Genome GPCR Mining and Annotation

Candidate sequences were mined from the *A. oligospora* ATCC 24927 reference assembly AOL24927_1.0 (NCBI accession GCA_000225545.1; WGS accession ADOT00000000) [9]. Proteome-wide transmembrane topology was predicted with TMbed [33], and proteins with six, seven or eight predicted transmembrane helices were taken forward for domain annotation with InterProScan v5.37-76.0 [34]. Domain descriptions associated with established fungal GPCR classes were manually reviewed. The initial inventory contained 89 proteins: 84 with seven predicted transmembrane helices and five with six. Two of the six-transmembrane entries, AOL_s00076g157.1 and AOL_s00188g124.1, had only generic transmembrane-protein annotations and lacked GPCR-domain support. They were retained in the initial phylogenetic and structural analyses but excluded from transcriptome-based prioritization, leaving 87 retained candidates. Candidate status denotes sequence-based annotation rather than experimentally validated receptor activity. Protein orthology between the ATCC 24927 and TWF154 strains was inferred using OrthoFinder v3.1.4 with DIAMOND and default parameters. A custom Python 3 script mapped OrthoFinder sequence IDs to the original protein and gene IDs and integrated orthogroup assignments and inferred orthologues. One-to-one, one-to-many, many-to-one and many-to-many relationships were retained in the ID mapping table.

### 4.3. Phylogenetic Analysis

Full-length sequences of the 89 initial inventory proteins were aligned using the automatic strategy in MAFFT v7. A maximum-likelihood tree was inferred with IQ-TREE 2. ModelFinder selected Q.pfam+F+I+R5 according to the Bayesian information criterion, and branch support was evaluated with 1000 ultrafast bootstrap replicates. The single-species receptor tree was midpoint-rooted using phangorn. Nodes with ultrafast bootstrap support ≥ 95 are highlighted in Figure 1.

### 4.4. AlphaFold2-Multimer Modeling and Gα Preference Scores

The 89 initial inventory proteins were paired individually with full-length AoGpa1, AoGpa2 and AoGpa3, yielding 267 receptor–Gα combinations. Models were generated using LocalColabFold 1.5.5 (commit e8ebd9a612b9da5fe12bbbc09513d5c52a9af633) with AlphaFold2-Multimer v3 weights on an NVIDIA RTX 3090 GPU. The archived configuration specifies MMseqs2 UniRef plus environmental-sequence MSAs, unpaired_paired MSA pairing with the greedy strategy, model weights 1–3, a maximum of three recycles with an early-stop tolerance of 0.5, random seed 0 and no structural templates. The original screen yielded 801 scored models. For each receptor–Gα pair, all three model scores were retained. Gα partners were ranked within each receptor by the maximum ipTM, and rankings were compared with those obtained from the mean ipTM and from each matched model weight.

The six combinations of AoGrn1 or AoGrn2 with the three Gα partners were additionally evaluated with random seed 101 using the archived MSAs and otherwise matched settings. Partner rankings and score distributions were compared across seeds. Interface PAE, pLDDT, α5-helix placement and receptor–Gα contacts were used as model-quality and geometry descriptors. pDockQ saturated at 0.742 and was excluded from partner ranking. The ipTM margins were treated as descriptive score differences without assigning calibrated biological selectivity classes. Gβγ and a defined nucleotide state were not included in the complex predictions. Model weights and random seeds were treated as computational sensitivity settings, not independent biological replicates.

### 4.5. Public Transcriptome Collection and Receptor Prioritization

Processed expression and count matrices were collected from GEO series for *Arthrobotrys oligospora* ATCC 24927. The full resource comprised 12 series, 213 GEO sample records and 19 expression/count matrices (Supplementary Table S8). Different matrix representations could contain the same samples and were not treated as additional biological replicates. Nine series with evaluable WT time-induction contrasts were retained for Figure 2a after excluding GSE253759 because of its similarity to GSE215079 and GSE303416 because its sample mapping was provisional. GSE322740 was excluded from the WT-induction display because its day-5 aerial-hypha design did not provide a time-induction contrast. Induction conditions were series-specific and included nitrogen deficiency in GSE212735. TPM, FPKM and raw-count matrices were analyzed separately; absolute abundance values were not pooled across units.

For the screening-effect tables used for support counting and candidate ranking, processed TPM/FPKM values were transformed as log_2_(value + 0.1). WT time-induction and same-time mutant-versus-WT effects were calculated as differences between group means on the transformed scale and evaluated using two-sided Welch *t* tests. Benjamini–Hochberg adjustment was applied within each matrix-by-contrast-type-by-time family. A supported screening contrast required both adjusted q < 0.05 and |log_2_ fold change| ≥ 1. Within each accession, the WT response was summarized by the median log_2_ fold change across the available screening contrasts, which could include multiple time points and processed matrices. A receptor was counted as supported by a series when at least one underlying WT-induction contrast met both criteria; multiple supported contrasts within a series contributed only one supporting series. Figure 2a support symbols follow this contrast-level definition, whereas cell colors represent the median effects. Additional within-series analyses used limma for processed expression matrices and DESeq2 v1.46.0 for raw count matrices. These model results were retained separately from the screening effects used for support counting and ranking.

Candidate eligibility required membership in the 87 retained GPCR candidates, responsiveness to nematode exposure, and expression-response support in at least one osmosensing MAPK and one cAMP–PKA perturbation dataset. Sixteen candidates met these criteria. After excluding GIN2, GIN6 and GIN9 as previously named or examined candidates, the remaining 13 were ranked. For each of AoHog1, AoOpy2, AoAcy and AoPkaC1, mutant-versus-WT screening log_2_ fold changes were summarized by their median across the available time points. The ranking score was the sum of the largest absolute regulator median within each module: AoHog1/AoOpy2 for osmosensing MAPK and AoAcy/AoPkaC1 for cAMP–PKA. Sensitivity analyses reassessed WT-response support after excluding GSE253759 and GSE303416 individually and jointly, and after removing each of the 11 original WT-induction series in turn (Supplementary Table S9). Alternative rankings replaced each module maximum with the median absolute regulator effect or used each of the four combinations containing one regulator from each module (Supplementary Table S10). Structural scores did not enter the transcriptome eligibility or ranking rule. Complete source files, sample and matrix mappings, Figure 2 source values, screening effects, separate series-model results and analysis scripts are provided in Supplementary Data S5.

### 4.6. Gene Deletion Transformant Verification and Complementation

Protoplasts were prepared from mycelia obtained from 4- to 5-day-old PDA cultures. Mycelia were transferred to tryptone-glucose broth (10 g/L tryptone, 10 g/L glucose), incubated overnight without shaking and then cultured for 24 h at 28 °C and 160 rpm. Approximately 0.2 g of mycelium was washed with MN buffer (0.3 M NaCl, 0.3 M MgSO_4_) and digested at 31 °C and 180 rpm for 3–5 h with a 20 mL enzyme solution containing 6.25 mg/mL cellulase, 6.25 mg/mL snailase, and 1.25 mg/mL lysing enzyme. Released protoplasts were filtered, collected at 5000 rpm for 10 min, washed with KTC buffer (1.2 M KCl, 50 mM CaCl_2_, 10 mM Tris-HCl, pH 7.5), and resuspended in approximately 100 µL of KTC buffer.

Target-specific sgRNAs were synthesized commercially (GenScript, Nanjing, China) and folded by heating at 96 °C for 6 min followed by cooling to 23 °C at 0.1 °C s^−1^. Folded sgRNAs were incubated with SpCas9 protein (New England Biolabs, Ipswich, MA, USA) to assemble ribonucleoprotein complexes. For each target, the Cas9–sgRNA complex and donor DNA containing a hygromycin phosphotransferase (HPH) cassette flanked by approximately 2 kb 5′ and 3′ homology arms were mixed with 100 µL of protoplast suspension and incubated on ice for 40 min. After addition of 1 mL of PTC buffer (50% *w*/*v* PEG 6000, 50 mM CaCl_2_, 10 mM Tris-HCl, pH 7.5), samples were incubated at 28 °C for 30 min, transferred to 10 mL of PDAS recovery medium (200 g/L potato infusion, 205.2 g/L sucrose, 10 g/L molasses, 0.3 g/L yeast extract) and regenerated for 12 h at 28 °C and 160 rpm. Protoplasts were concentrated and plated on PDAS containing 100 µg mL^−1^ hygromycin B. Resistant colonies were recovered at 28 °C and screened by diagnostic PCR. Diagnostic primers were designed to anneal 50 bp upstream and 50 bp downstream of the respective target genes.

Hygromycin-resistant colonies were purified through five rounds of successive hyphal-tip transfers on selective plates and subsequently screened by diagnostic PCR using primers annealing approximately 50 bp upstream and downstream of the *AoGrn1* or *AoGrn2* coding regions. Homozygous candidate deletion mutants (Δ*AoGrn1* and Δ*AoGrn2*) were identified by the presence of a single *HPH*-containing amplicon larger than the wild-type (WT) locus product and the complete absence of the smaller WT-sized band (Figure S1). Representative locus-spanning amplicons were further subjected to Sanger sequencing to verify the targeted replacement (Figures S2 and S3). For each target gene, three PCR-verified single-colony transformants recovered from the same transformation reaction were maintained as separately labeled isolates for downstream phenotypic characterization. For genetic complementation, the full-length WT *AoGrn1* or *AoGrn2* coding sequence driven by the *oliC* promoter was introduced into protoplasts prepared from the respective deletion mutant pools via PEG-mediated transformation under nourseothricin selection.

### 4.7. Growth and Trap Formation Assays

Colony diameters were recorded daily from day 1 to day 6 for five independent culture plates per strain, with the same plates followed over time. The three deletion isolates for each gene were analyzed separately. A plate-level area under the diameter–time curve (AUC) was calculated by trapezoidal integration over days 1–5. Day 6 was retained in the plotted trajectories but excluded from the primary AUC because colony expansion was limited by the plate edge. All observations were retained. Growth trajectories and AUCs are shown as mean ± SD, with individual plate-level AUC values provided in Figure S4, Table S5, and Data S3.

For trap formation assays, 0.5 cm mycelial plugs were inoculated onto water-agar (WA) plates and pre-cultured at 28 °C for 30 h, followed by induction with approximately 200 live L4-stage *C. elegans* per plate. Trap density was assessed at 30 h post-induction. Prior to quantification, the bottom of each plate was divided into four equal sectors, and two randomly selected regions covering a combined area of 4.5 cm^2^ were examined per plate. Each closed individual hyphal loop (ring) was counted as a single trap, and the total count within the sampled area was divided by 4.5 to calculate the trap density (traps cm^−2^). All plates were coded so that the investigator was blinded to strain identity during counting. Three separately labeled deletion isolates were tested for each target gene (Δ*AoGrn1* and Δ*AoGrn2*), alongside the WT and the *AoGrn1* and *AoGrn2* complemented strains. Ten replicate plates were assayed per strain in a single experimental batch. Each plate contributed one density measurement, with isolates and plates retained as distinct levels of replication.

### 4.8. Nematode Extract (NE) Preparation

Approximately 50,000 mixed-stage *C. elegans* were washed five times with M9 buffer to remove surface *Escherichia coli* OP50, flash-frozen in liquid nitrogen, and pulverized. The homogenized tissue was resuspended in 10 mL of distilled water, clarified by centrifugation at 12,000× *g* to remove insoluble debris, and passed through a 0.22 μm membrane filter to clear residual particulate matter. The resulting filtrate from a single pooled extraction batch served as the nematode extract (NE) stock, corresponding to a nominal input of 5000 nematode equivalents mL^−1^, and was stored at −80 °C in single-use aliquots to prevent repeated freeze–thaw cycles. For BRET assays, the NE stock was first diluted tenfold in distilled water to prepare the highest working solution (500 nematode equivalents mL^−1^), followed by tenfold serial dilutions. In each assay well, 20 μL of the respective NE working dilution was added to achieve a final total reaction volume of 80 μL, resulting in an additional fourfold in-well dilution. Doses on the concentration–response axes are reported as relative working dilutions referenced to the 5000 equivalents mL^−1^ NE stock, because the exact active constituents and their molar concentrations were not determined.

### 4.9. BRET Assays

The amino acid sequences of the candidate receptors and heterotrimeric G-protein subunits—G*α* subunits AoGpa1 (AOL_s00075g181; UniProt: G1X8I2), AoGpa2 (AOL_s00109g19; G1XJZ0), and AoGpa3 (AOL_s00075g14; G1X815), the G*β* subunit (AOL_s00004g438; G1WYS7), and the G*γ* subunit (AOL_s00083g167; G1XGN6)—were derived from A. oligospora and codon optimized for expression in HEK293F cells. Candidate receptors were engineered with an N-terminal hemagglutinin (HA) signal peptide followed by a FLAG epitope tag. The BRET2 heterotrimeric G-protein sensor comprised a G*α* subunit C-terminally fused to Renilla luciferase 8 (G*α* − RLuc8, donor), a G*γ* subunit C-terminally fused to green fluorescent protein 2 (G*γ* − GFP2, acceptor), and an untagged G*β* subunit. Receptor and sensor constructs were transiently cotransfected into HEK293F cells using polyethylenimine (PEI; MCE, Monmouth Junction, NJ, USA) in Opti-MEM (Gibco, Grand Island, NY, USA) at a plasmid DNA mass ratio of 1:1:1:1 (receptor:G*α*:G*β*:G*γ*).

Approximately 48 h after transfection, cells were harvested, resuspended in BRET assay buffer (1 × HBSS supplemented with 20 mM HEPES and 0.1% BSA), and distributed into 96-well plates. Following the manufacturer’s instructions, the BRET2 substrate coelenterazine 400a was added, and donor (410 nm) and acceptor (515 nm) emissions were measured sequentially using a SP-Max 3500FL multimode microplate reader (Shanghai Shanpu Biotechnology, Shanghai, China) configured with a 1 min initial shake, a 100 ms delay, and a 1000 ms integration time at 4 min intervals. Five consecutive pre-treatment readings were collected to monitor baseline stability, after which cells were treated with serial NE working dilutions (prepared in BRET assay buffer) or ligand-free vehicle control (BRET assay buffer alone), followed by five post-treatment readings at 4 min intervals. Gin3-expressing and receptor-free sensor configurations were included in parallel as control conditions (Figure S7).

For each well, the BRET ratio (*R*) was calculated as the acceptor emission (515 nm) divided by the donor emission (410 nm). The pre-treatment baseline (*R*_0_) for each well was defined as the average of the final two stabilized baseline readings prior to ligand addition, and the fractional response at post-treatment time *t* was calculated as *f*(*t*) = [*R*(*t*) − *R*_0_]/*R*_0_. To correct for ligand-independent baseline drift and buffer effects, the vehicle-corrected response was calculated as ΔBRET(*t*) = *f*_NE_(*t*) − *f*_vehicle_(*t*), where *f*_vehicle_(*t*) was derived from identically transfected control wells treated with vehicle (BRET assay buffer without ligand) using the corresponding sensor configuration. Concentration–response analyses were evaluated at the third post-treatment reading (12 min after addition), which was selected to capture the stabilized response window. Each condition was tested in three independent biological experiments with two technical replicate wells per experiment. Technical duplicates were first averaged within each experiment, and the resulting experiment-level means (*n* = 3 independent experiments) were used to calculate group means and error bars.

Concentration–response trajectories were analyzed using the preconfigured three-parameter log(agonist) − versus − response model in GraphPad Prism version 11.0.0 with a fixed standard Hill slope of 1.0, estimating Bottom, Top (Span = Top − Bottom), and logEC_50_ alongside profile-likelihood 95% confidence intervals. Vehicle measurements were used in response normalization and displayed separately from the nonzero log-dose fits. Apparent EC_50_ values for crude NE are interpretable only on the stated relative working-dilution scale. Where response spans were minimal or non-monotonic—resulting in unstable parameter estimates or unidentifiable confidence intervals—fitted curves are shown solely to illustrate trends without reporting numeric EC_50_ values. A fitted curve alone does not establish a receptor-dependent response or exclude non-specific optical interference.

### 4.10. Immunofluorescence Microscopy

Cells grown on glass coverslips were rinsed once with PBS supplemented with Ca^2+^ and Mg^2+^ (PBS^++^; containing 0.9 mM CaCl_2_ and 0.5 mM MgCl_2_) and fixed with 4% paraformaldehyde (diluted from 8% formaldehyde, Electron Microscopy Sciences are Hatfield, PA, USA, #157-8) in PBS^++^ for 10 min at room temperature (RT). Following fixation, the cells were rinsed once with PBS^++^ and incubated with quenching solution (50 mM NH_4_Cl in PBS^++^) for 5 min at RT to quench residual aldehyde fluorescence, followed by three washes with PBS^++^. Subsequently, the cells were permeabilized with 0.2% Triton X-100 in PBS^++^ for 15 min at RT and rinsed once with PBS^++^. Non-specific binding sites were blocked by incubating the cells in blocking buffer (3% FBS in PBS^++^) for 1 h at RT.

After blocking, the cells were incubated with a rabbit polyclonal anti-HA tag primary antibody (Abcam (Cambridge, UK), #ab9110; 1:500 dilution) diluted in blocking buffer for 1 h at RT. Following three washes with blocking buffer, the cells were incubated with CoraLite488-conjugated goat anti-rabbit IgG(H+L) secondary antibody (Proteintech (Rosemont, IL, USA), #SA00013-2; 1:500 dilution) diluted in blocking buffer for 1 h at RT in the dark. For nuclear counterstaining, the cells were washed twice with PBS^++^, stained with DAPI (MedChemExpress (Monmouth Junction, NJ, USA), #HY-D0814; 10 μg/mL in PBS^++^) for 5 min in the dark, and washed twice again with PBS^++^. Finally, the coverslips were briefly dipped in ddH_2_O to remove residual salts, drained on a Kimwipe, and mounted cell-side down onto glass slides using 10 μL of ProLong Gold Antifade Mountant (Invitrogen, Carlsbad, CA, USA, #P36930). Fluorescence images were acquired using a Zeiss LSM 980 confocal laser-scanning microscope (Carl Zeiss, Jena, Germany).

### 4.11. Detailed Interface and Conformational Analysis

Five AlphaFold2-Multimer models were generated for each combination of AoGrn1 or AoGrn2 with AoGpa1, AoGpa2 or AoGpa3. The highest-ipTM model was used for visualization and primary interface analysis, with interface PAE providing an additional confidence measure. Contacting receptor residues were assigned to transmembrane helices, intracellular loops or the C-terminal tail. Core-interface analyses included the seven-transmembrane bundle and intracellular loops; lower-confidence C-terminal-tail contacts were analyzed separately. High-confidence contacts required pLDDT ≥ 70 for both interacting residues and interface PAE < 15 Å. Interface size, buried solvent-accessible surface area (BSA), interaction chemistry and Gα α5-helix participation were quantified for each pair. Receptor-side interface residue sets were compared across the three highest-ranked models to assess interface reproducibility.

### 4.12. Total RNA Extraction and Quantitative Reverse-Transcription PCR

Mycelia of WT, Δ*AoGrn1*, Δ*AoGrn2* and Δ*AoGrn3* were sampled at baseline and 10 h after the addition of live *C. elegans*. Three biological samples were analyzed per genotype–time condition, with three technical reactions per sample and assay. Samples were ground in liquid nitrogen, and total RNA was extracted using the TaKaRa MiniBEST Universal RNA Extraction Kit (Takara Bio, Kusatsu, Japan; cat. no. 9767). Genomic DNA was removed using gDNA Eraser before reverse transcription with PrimeScript RT Master Mix (Takara Bio). The cDNA templates were standardized to an RNA-equivalent concentration of 60 ng/μL. qPCR was performed on a Roche LightCycler 480 (Roche, Basel, Switzerland) using TB Green Premix Ex Taq II (Takara Bio; cat. no. RR820A). The amplification program consisted of 95 °C for 2 min, followed by 35 cycles of 95 °C for 10 s, 58 °C for 30 s and 72 °C for 30 s. Melt-curve analysis was performed from 60 to 95 °C. AoGrn1, AoGrn2 and AoGrn3 were assayed across the four genotypes, with Tub as the reference assay. Primer sequences, amplicon lengths and standard-curve results are provided in Supplementary Tables S4 and S6.

Instrument-reported crossing-point (Cp) values were used as cycle-threshold estimates. Detected technical reactions were averaged within each biological sample, and ΔCt was calculated as mean Cp(target) − mean Cp(Tub). For each target, ΔΔCt was calculated by subtracting the mean ΔCt of the three WT baseline samples, and relative abundance was calculated as 2^(−ΔΔCt) [35]. This calibration sets the WT baseline geometric mean to 1 while retaining variation among individual baseline samples. Reactions without an instrument Cp readout were designated as not detected (ND). Zero codes representing ND in the source workbook were excluded from Ct calculations and were not interpreted as zero expression. All detected reactions were retained, including 14 triplicates with a Ct SD > 0.5 or a range > 1 cycle. ND targets were summarized by detection frequency without assigning numerical fold changes or performing *t* tests. Source Cp values and derived analyses are provided in Supplementary Table S7, Figure S6 and Data S2.

Relative-abundance plots show individual biological samples and arithmetic mean ± SD. Recorded replicate identifiers (1–3) were used to match samples for paired analyses. Two-sided paired *t* tests were applied to −ΔCt contrasts, with three pairs and two degrees of freedom. Nine within-genotype time comparisons, twelve same-time mutant-versus-WT comparisons and six genotype-by-time contrasts formed a global family of 27 tests, with Holm adjustment across the complete family. Pointwise 95% t intervals for log_2_ contrasts were back-transformed to fold units. Exact sign-flip sensitivity analyses enumerated all eight sign assignments; with three pairs, the minimum attainable two-sided *p* value was 0.25. Family-specific adjusted values were retained in the source data, whereas Figure S6 displays the global Holm correction.

Standard-dilution measurements were analyzed separately from the biological expression samples (Supplementary Table S6 and Figure S5). Estimated amplification efficiencies were 99.14% for AoGrn1, 99.39% for AoGrn2, 99.96% for AoGrn3 and 109.04% for Tub. Comparative-Ct fold estimates and their statistical summaries assumed an amplification factor of 2 for both target and reference and did not incorporate these efficiency estimates. The difference between the estimated target and reference efficiencies therefore limits the quantitative interpretation of the reported fold changes.

### 4.13. Statistical Analysis

Trap density and colony-growth measurements are reported as mean ± SD, with the plate as the measurement unit. Deletion isolates were analyzed separately. Trap-density comparisons followed the analysis reported in Figure 3e: two-sided unpaired Welch *t* tests with Holm adjustment across 17 comparisons, comprising eleven non-WT-versus-WT comparisons and six complemented-strain-versus-corresponding-deletion-isolate comparisons. The ten plates per strain came from one induction batch and were not treated as ten independent transformation events. The single batch limits inference about between-batch reproducibility.

For trap induction assays, comparisons were supported by exact rank-based permutation tests (enumerating all 184,756 allocations), with full details provided in Data S7. Growth AUCs over days 1–5 were compared with WT using two-sided exact Mann–Whitney U tests, enumerating all 252 allocations of the ten plate labels in each comparison, with Holm adjustment jointly across eight non-WT-versus-WT comparisons. The omnibus Kruskal–Wallis statistic was assessed using 99,999 permutations of plate labels. Welch tests and AUCs including day 6 were used as sensitivity analyses. Transcriptome *p* values were adjusted by the Benjamini–Hochberg procedure within the stated analysis families. Paired receptor-level comparisons of AlphaFold-derived metrics used two-sided Wilcoxon signed-rank tests; model weights and seeds were treated as sensitivity settings. The qPCR analysis and global 27-comparison correction are described in Section 4.12. Exact *p* values and the scope of each multiple-comparison correction are reported with the corresponding results. Adjusted *p* or q < 0.05 was used as the stated significance threshold; a nonsignificant result was not interpreted as evidence of equivalence.

## 5. Conclusions

Genome-scale receptor mapping and cross-study transcriptional prioritization identified AoGrn1 and AoGrn2 as candidate receptors contributing to nematode-induced trap formation in *A. oligospora*. Genetic deletion and complementation analyses supported their involvement in trap development, while transcriptional profiling confirmed their induction upon prey exposure. Furthermore, heterologous BRET assays in HEK293F cells revealed distinct nematode extract-elicited response profiles for the AoGrn1–AoGpa2 and AoGrn2–AoGpa1 combinations. Together with structural interface modeling, these findings establish candidate receptor–Gα pairings and predicted contact residues for future studies on how *A. oligospora* senses prey cues to initiate predatory development.

## Data Availability

The *Arthrobotrys oligospora* ATCC 24927 reference genome used in this study is available from NCBI GenBank assembly accession GCA_000225545.1; https://www.ncbi.nlm.nih.gov/datasets/genome/GCA_000225545.1/ (accessed on 22 October 2026). The public transcriptome datasets analyzed here are available from the NCBI Gene Expression Omnibus https://www.ncbi.nlm.nih.gov/geo/ (accessed on 22 October 2026) under accessions GSE210886, GSE212735, GSE213542, GSE215079, GSE245014, GSE253759, GSE301423, GSE301452, GSE303416, GSE306156, GSE322548 and GSE322740. The qRT-PCR crossing-point values, derived expression data and analysis notebook, together with the processed source data for Figure 2, are provided in Supplementary Data S2, Data S5.

## Supplementary Materials

The following supporting information can be downloaded at: https://www.mdpi.com/article/10.3390/ijms27219599/s1.

## Abbreviations

The following abbreviations are used in this manuscript:

AF2M	AlphaFold2-Multimer
BRET	bioluminescence resonance energy transfer
cAMP–PKA	cyclic AMP–protein kinase A
FPKM	fragments per kilobase per million mapped fragments
GEO	Gene Expression Omnibus
GPCR	G protein-coupled receptor
HPH	hygromycin phosphotransferase
hpe	hours post-exposure
ICL	intracellular loop
ipTM	interface-predicted TM-score
MAPK	mitogen-activated protein kinase
NE	nematode extract
PAE	predicted aligned error
TPM	transcripts per million
WT	wild type
Cp	crossing point
qRT-PCR	quantitative reverse-transcription polymerase chain reaction
ND	not detected
SD	standard deviation
SEM	standard error of the mean
