# Supplementary material for: Transcriptome-Guided Identification of Two GPCR Candidates Contributing to Trap Formation in Arthrobotrys oligospora
Source: Int J Mol Sci. 2026 Oct 27;27(21):9599. doi: 10.3390/ijms27219599 (PMC13763024; doi:10.3390/ijms27219599)
Supplement: Supplementary file 1 [file ijms-27-09599-s001.zip › ijms-4611842-supplementary.pdf]

Supplementary Materials

Genome-Wide GPCR Mapping Links AoGPR200 and AoGPRPR8 to Trap Formation and Distinct Gα Responses in Arthrobotrys oligospora

International Journal of Molecular Sciences | Manuscript ijms-4611842

Junjie Liu, Yunlong Li, Fang Zhan, Mengsha Deng, Yuhua Wang and Xin Wang

Contents

Supplementary tables

Table S1. Complete inventory of 89 candidate GPCR proteins.
Table S2. Mapping of prioritized receptor codes, loci and UniProt accessions.
Table S3. Fungal strain and genotype classes.
Table S4. Oligonucleotides for construct generation, sgRNA design and qRT-PCR.
Table S5. Plate-level growth AUC statistics and sensitivity analyses.
Table S6. RT-qPCR primers and assay performance.
Table S7. Source Ct measurements and biological-replicate expression analyses.
Table S8. Public transcriptome resource and matrix inventory.
Table S9. WT-response support and exclusion sensitivity.
Table S10. Candidate ranking and regulator-effect sensitivity.

Supplementary figures

Figure S1. Full diagnostic PCR gel panels.
Figure S2. Representative Sanger evidence for HPH-cassette junctions.
Figure S3. All-candidate junction evidence and HPH design-coordinate coverage.
Figure S4. Plate-level growth AUC over days 1–5.
Figure S5. Standard curves for RT-qPCR assays.
Figure S6. Relative transcript abundance of Grn1, Grn2 and Grn3.
Figure S7. BRET positive and receptor-free negative controls.

Source data

Data S1: GPCR inventory (XLSX/TSV). Data S2: verified qPCR source workbooks, all Ct values and biological-replicate analyses. Data S3: growth plate measurements and statistical outputs. Data S4: supplied diagnostic gel and BRET control images.

Data S5: Public transcriptome inputs, sample and matrix mapping, Figure 2 source tables, exclusion and ranking sensitivity, and analysis scripts.

Table S1. Complete inventory of 87 candidate GPCR proteins.

Sequence identifier	Study code	TM	Length (aa)	Annotation count	Status	Supplied GPCR-associated annotations
AOL_s00004g178.1	—	7	425	2	Candidate	PTH11-LIKE INTEGRAL MEMBRANE PROTEIN (AFU_ORTHOLOGUE AFUA_5G11245); Fungal rhodopsin domain
AOL_s00007g309.1	Grn1	7	384	2	Candidate	PTH11-LIKE INTEGRAL MEMBRANE PROTEIN (AFU_ORTHOLOGUE AFUA_5G11245); Fungal rhodopsin domain
AOL_s00173g258.2	—	7	419	2	Candidate	PTH11-LIKE INTEGRAL MEMBRANE PROTEIN (AFU_ORTHOLOGUE AFUA_5G11245); Fungal rhodopsin domain
AOL_s00054g428.1	—	7	561	2	Candidate	LUNG SEVEN TRANSMEMBRANE RECEPTOR; GOST, seven transmembrane domain
AOL_s00076g86.1	—	7	391	2	Candidate	PTH11-LIKE INTEGRAL MEMBRANE PROTEIN (AFU_ORTHOLOGUE AFUA_5G11245); Fungal rhodopsin domain
AOL_s00080g416.1	—	7	433	2	Candidate	PTH11-LIKE INTEGRAL MEMBRANE PROTEIN (AFU_ORTHOLOGUE AFUA_5G11245); Fungal rhodopsin domain
AOL_s00140g54.6	—	7	391	2	Candidate	Fungal rhodopsin domain; PTH11-LIKE INTEGRAL MEMBRANE PROTEIN (AFU_ORTHOLOGUE AFUA_5G11245)
AOL_s00054g150.1	Grn3	7	375	2	Candidate	Fungal rhodopsin domain; PTH11-LIKE INTEGRAL MEMBRANE PROTEIN (AFU_ORTHOLOGUE AFUA_5G11245)
AOL_s00054g92.1	—	7	476	2	Candidate	PTH11-LIKE INTEGRAL MEMBRANE PROTEIN (AFU_ORTHOLOGUE AFUA_5G11245); Fungal rhodopsin domain
AOL_s00006g519.1	—	7	402	1	Candidate	G-PROTEIN COUPLED RECEPTORS FAMILY 3 PROFILE DOMAIN-CONTAINING PROTEIN
AOL_s00210g385.1	—	7	412	2	Candidate	PTH11-LIKE INTEGRAL MEMBRANE PROTEIN (AFU_ORTHOLOGUE AFUA_5G11245); Fungal rhodopsin domain
AOL_s00076g508.1	—	7	365	2	Candidate	PTH11-LIKE INTEGRAL MEMBRANE PROTEIN (AFU_ORTHOLOGUE AFUA_5G11245); Fungal rhodopsin domain
AOL_s00006g420.1	—	7	709	5	Candidate	G protein-coupled glucose receptor regulating Gpa2; 7tm_classA_rhodopsin-like; Family A G protein-coupled receptor-like; G PROTEIN-COUPLED RECEPTOR 157-RELATED; G protein-coupled glucose receptor regulating Gpa2 C-term
AOL_s00170g54.1	—	7	475	2	Candidate	Fungal rhodopsin domain; PTH11-LIKE INTEGRAL MEMBRANE PROTEIN (AFU_ORTHOLOGUE AFUA_5G11245)
AOL_s00078g598.1	—	7	459	1	Candidate	G-PROTEIN COUPLED RECEPTORS FAMILY 3 PROFILE DOMAIN-CONTAINING PROTEIN
AOL_s00193g107.1	—	7	388	2	Candidate	Fungal rhodopsin domain; PTH11-LIKE INTEGRAL MEMBRANE PROTEIN (AFU_ORTHOLOGUE AFUA_5G11245)
AOL_s00081g30.1	—	7	505	1	Candidate	PTH11-LIKE INTEGRAL MEMBRANE PROTEIN (AFU_ORTHOLOGUE AFUA_5G11245)
AOL_s00043g195.1	—	7	405	2	Candidate	PTH11-LIKE INTEGRAL MEMBRANE PROTEIN (AFU_ORTHOLOGUE AFUA_5G11245); Fungal rhodopsin domain
AOL_s00215g796.1	—	7	483	2	Candidate	Fungal rhodopsin domain; PTH11-LIKE INTEGRAL MEMBRANE PROTEIN (AFU_ORTHOLOGUE AFUA_5G11245)
AOL_s00188g342.1	—	7	428	2	Candidate	PTH11-LIKE INTEGRAL MEMBRANE PROTEIN (AFU_ORTHOLOGUE AFUA_5G11245); Fungal rhodopsin domain
AOL_s00043g705.1	—	7	575	3	Candidate	G_PROTEIN_RECEP_F2_4 DOMAIN-CONTAINING PROTEIN; 7 transmembrane receptor (Secretin family); G-protein coupled receptors family 2 profile 2.
AOL_s00188g346.1	—	7	433	2	Candidate	PTH11-LIKE INTEGRAL MEMBRANE PROTEIN (AFU_ORTHOLOGUE AFUA_5G11245); Fungal rhodopsin domain
AOL_s00215g417.1	—	7	416	2	Candidate	Fungal rhodopsin domain; PTH11-LIKE INTEGRAL MEMBRANE PROTEIN (AFU_ORTHOLOGUE AFUA_5G11245)
AOL_s00097g309.3	—	7	405	2	Candidate	PTH11-LIKE INTEGRAL MEMBRANE PROTEIN (AFU_ORTHOLOGUE AFUA_5G11245); Fungal rhodopsin domain
AOL_s00007g90.1	—	7	535	2	Candidate	PTH11-LIKE INTEGRAL MEMBRANE PROTEIN (AFU_ORTHOLOGUE AFUA_5G11245); Fungal rhodopsin domain
AOL_s00080g10.1	—	6	338	2	Candidate	Fungal rhodopsin domain; PTH11-LIKE INTEGRAL MEMBRANE PROTEIN (AFU_ORTHOLOGUE AFUA_5G11245)
AOL_s00004g290.1	—	7	488	2	Candidate	PTH11-LIKE INTEGRAL MEMBRANE PROTEIN (AFU_ORTHOLOGUE AFUA_5G11245); Fungal rhodopsin domain
AOL_s00215g924.1	—	7	406	2	Candidate	PTH11-LIKE INTEGRAL MEMBRANE PROTEIN (AFU_ORTHOLOGUE AFUA_5G11245); Fungal rhodopsin domain
AOL_s00075g112.1	—	7	490	2	Candidate	PTH11-LIKE INTEGRAL MEMBRANE PROTEIN (AFU_ORTHOLOGUE AFUA_5G11245); Fungal rhodopsin domain
AOL_s00210g301.1	—	7	506	2	Candidate	PTH11-LIKE INTEGRAL MEMBRANE PROTEIN (AFU_ORTHOLOGUE AFUA_5G11245); Fungal rhodopsin domain
AOL_s00215g915.1	—	7	440	2	Candidate	PTH11-LIKE INTEGRAL MEMBRANE PROTEIN (AFU_ORTHOLOGUE AFUA_5G11245); Fungal rhodopsin domain

Sequence identifier	Study code	TM	Length (aa)	Annotation count	Status	Supplied GPCR-associated annotations
AOL_s00215g603.1	—	7	386	1	Candidate	G-PROTEIN COUPLED RECEPTORS FAMILY 3 PROFILE DOMAIN-CONTAINING PROTEIN
AOL_s00188g142.1	—	7	853	2	Candidate	PTH11-LIKE INTEGRAL MEMBRANE PROTEIN (AFU_ORTHOLOGUE AFUA_5G11245); Fungal rhodopsin domain
AOL_s00075g223.1	—	7	370	4	Candidate	PHEROMONE ALPHA FACTOR RECEPTOR; Pheromone alpha factor receptor.; Fungal pheromone mating factor STE2 GPCR signature; Fungal pheromone mating factor STE2 GPCR
AOL_s00043g223.1	—	7	449	2	Candidate	PTH11-LIKE INTEGRAL MEMBRANE PROTEIN (AFU_ORTHOLOGUE AFUA_5G11245); Fungal rhodopsin domain
AOL_s00215g906.3	—	7	483	2	Candidate	Fungal rhodopsin domain; PTH11-LIKE INTEGRAL MEMBRANE PROTEIN (AFU_ORTHOLOGUE AFUA_5G11245)
AOL_s00176g42.1	Grn2	7	457	2	Candidate	PTH11-LIKE INTEGRAL MEMBRANE PROTEIN (AFU_ORTHOLOGUE AFUA_5G11245); Fungal rhodopsin domain
AOL_s00054g223.1	—	7	459	2	Candidate	Fungal rhodopsin domain; PTH11-LIKE INTEGRAL MEMBRANE PROTEIN (AFU_ORTHOLOGUE AFUA_5G11245)
AOL_s00054g63.1	—	7	550	2	Candidate	PTH11-LIKE INTEGRAL MEMBRANE PROTEIN (AFU_ORTHOLOGUE AFUA_5G11245); Fungal rhodopsin domain
AOL_s00007g229.1	—	7	411	2	Candidate	Fungal rhodopsin domain; PTH11-LIKE INTEGRAL MEMBRANE PROTEIN (AFU_ORTHOLOGUE AFUA_5G11245)
AOL_s00110g16.1	—	7	420	2	Candidate	PTH11-LIKE INTEGRAL MEMBRANE PROTEIN (AFU_ORTHOLOGUE AFUA_5G11245); Fungal rhodopsin domain
AOL_s00110g337.1	—	7	448	2	Candidate	Fungal rhodopsin domain; PTH11-LIKE INTEGRAL MEMBRANE PROTEIN (AFU_ORTHOLOGUE AFUA_5G11245)
AOL_s00043g731.1	—	7	426	2	Candidate	PTH11-LIKE INTEGRAL MEMBRANE PROTEIN (AFU_ORTHOLOGUE AFUA_5G11245); Fungal rhodopsin domain
AOL_s00043g324.1	—	7	331	2	Candidate	PTH11-LIKE INTEGRAL MEMBRANE PROTEIN (AFU_ORTHOLOGUE AFUA_5G11245); Fungal rhodopsin domain
AOL_s00054g200.1	—	7	445	2	Candidate	PTH11-LIKE INTEGRAL MEMBRANE PROTEIN (AFU_ORTHOLOGUE AFUA_5G11245); Fungal rhodopsin domain
AOL_s00076g249.1	—	7	406	7	Candidate	G PROTEIN-COUPLED RECEPTOR 157-RELATED; Family A G protein-coupled receptor-like; G-protein coupled receptors family 2 profile 2.; G protein-coupled glucose receptor regulating Gpa2; GCR1-cAMP receptor family signature; G protein-coupled glucose receptor regulating Gpa2 C-term; G-protein coupled receptors family 1 profile.
AOL_s00176g24.1	—	7	477	2	Candidate	Fungal rhodopsin domain; PTH11-LIKE INTEGRAL MEMBRANE PROTEIN (AFU_ORTHOLOGUE AFUA_5G11245)
AOL_s00054g626.1	—	7	309	5	Candidate	Bacterial opsin signature; Bac_rhodopsin_2; 7tm_Opsin-1_euk; Family A G protein-coupled receptor-like; Bacteriorhodopsin-like protein
AOL_s00170g20.1	—	7	560	2	Candidate	PTH11-LIKE INTEGRAL MEMBRANE PROTEIN (AFU_ORTHOLOGUE AFUA_5G11245); Fungal rhodopsin domain
AOL_s00215g444.1	—	7	411	2	Candidate	Fungal rhodopsin domain; PTH11-LIKE INTEGRAL MEMBRANE PROTEIN (AFU_ORTHOLOGUE AFUA_5G11245)
AOL_s00097g260.1	—	7	409	2	Candidate	PTH11-LIKE INTEGRAL MEMBRANE PROTEIN (AFU_ORTHOLOGUE AFUA_5G11245); Fungal rhodopsin domain
AOL_s00076g465.1	—	7	420	2	Candidate	PTH11-LIKE INTEGRAL MEMBRANE PROTEIN (AFU_ORTHOLOGUE AFUA_5G11245); Fungal rhodopsin domain
AOL_s00097g69.1	—	7	413	2	Candidate	Fungal rhodopsin domain; PTH11-LIKE INTEGRAL MEMBRANE PROTEIN (AFU_ORTHOLOGUE AFUA_5G11245)
AOL_s00078g446.1	—	7	330	2	Candidate	PTH11-LIKE INTEGRAL MEMBRANE PROTEIN (AFU_ORTHOLOGUE AFUA_5G11245); Fungal rhodopsin domain
AOL_s00215g252.1	—	7	500	2	Candidate	PTH11-LIKE INTEGRAL MEMBRANE PROTEIN (AFU_ORTHOLOGUE AFUA_5G11245); Fungal rhodopsin domain
AOL_s00210g381.1	—	6	377	2	Candidate	Fungal rhodopsin domain; PTH11-LIKE INTEGRAL MEMBRANE PROTEIN (AFU_ORTHOLOGUE AFUA_5G11245)
AOL_s00083g156.1	—	7	688	2	Candidate	Fungal rhodopsin domain; PTH11-LIKE INTEGRAL MEMBRANE PROTEIN (AFU_ORTHOLOGUE AFUA_5G11245)
AOL_s00076g286.1	—	7	411	2	Candidate	PTH11-LIKE INTEGRAL MEMBRANE PROTEIN (AFU_ORTHOLOGUE AFUA_5G11245); Fungal rhodopsin domain
AOL_s00054g605.1	—	7	424	2	Candidate	PTH11-LIKE INTEGRAL MEMBRANE PROTEIN (AFU_ORTHOLOGUE AFUA_5G11245); Fungal rhodopsin domain
AOL_s00169g261.1	—	7	371	2	Candidate	PTH11-LIKE INTEGRAL MEMBRANE PROTEIN (AFU_ORTHOLOGUE AFUA_5G11245); Fungal rhodopsin domain
AOL_s00173g137.1	—	7	379	2	Candidate	Fungal rhodopsin domain; PTH11-LIKE INTEGRAL MEMBRANE PROTEIN (AFU_ORTHOLOGUE AFUA_5G11245)
AOL_s00043g267.1	—	7	422	2	Candidate	PTH11-LIKE INTEGRAL MEMBRANE PROTEIN (AFU_ORTHOLOGUE AFUA_5G11245); Fungal rhodopsin domain

Sequence identifier	Study code	TM	Length (aa)	Annotation count	Status	Supplied GPCR-associated annotations
AOL_s00054g135.1	—	7	558	1	Candidate	G PROTEIN-COUPLED RECEPTOR 157-RELATED
AOL_s00076g553.1	—	7	587	2	Candidate	PTH11-LIKE INTEGRAL MEMBRANE PROTEIN (AFU_ORTHOLOGUE AFUA_5G11245); Fungal rhodopsin domain
AOL_s00188g320.1	—	7	458	2	Candidate	PTH11-LIKE INTEGRAL MEMBRANE PROTEIN (AFU_ORTHOLOGUE AFUA_5G11245); Fungal rhodopsin domain
AOL_s00004g22.1	—	7	506	2	Candidate	PTH11-LIKE INTEGRAL MEMBRANE PROTEIN (AFU_ORTHOLOGUE AFUA_5G11245); Fungal rhodopsin domain
AOL_s00054g291.1	—	7	521	1	Candidate	PTH11-LIKE INTEGRAL MEMBRANE PROTEIN (AFU_ORTHOLOGUE AFUA_5G11245)
AOL_s00215g562.1	—	7	430	2	Candidate	Fungal rhodopsin domain; PTH11-LIKE INTEGRAL MEMBRANE PROTEIN (AFU_ORTHOLOGUE AFUA_5G11245)
AOL_s00079g307.1	—	7	725	4	Candidate	PHEROMONE A FACTOR RECEPTOR; 7tmD_STE3; Pheromone A receptor; Fungal pheromone STE3 GPCR signature
AOL_s00110g307.1	—	7	407	2	Candidate	Fungal rhodopsin domain; PTH11-LIKE INTEGRAL MEMBRANE PROTEIN (AFU_ORTHOLOGUE AFUA_5G11245)
AOL_s00054g270.1	—	7	383	2	Candidate	PTH11-LIKE INTEGRAL MEMBRANE PROTEIN (AFU_ORTHOLOGUE AFUA_5G11245); Fungal rhodopsin domain
AOL_s00078g563.1	—	6	418	2	Candidate	PTH11-LIKE INTEGRAL MEMBRANE PROTEIN (AFU_ORTHOLOGUE AFUA_5G11245); Fungal rhodopsin domain
AOL_s00169g110.1	—	7	451	2	Candidate	PTH11-LIKE INTEGRAL MEMBRANE PROTEIN (AFU_ORTHOLOGUE AFUA_5G11245); Fungal rhodopsin domain
AOL_s00109g63.1	—	7	408	2	Candidate	Fungal rhodopsin domain; PTH11-LIKE INTEGRAL MEMBRANE PROTEIN (AFU_ORTHOLOGUE AFUA_5G11245)
AOL_s00097g290.1	—	7	573	2	Candidate	PTH11-LIKE INTEGRAL MEMBRANE PROTEIN (AFU_ORTHOLOGUE AFUA_5G11245); Fungal rhodopsin domain
AOL_s00043g738.2	—	7	415	2	Candidate	Fungal rhodopsin domain; PTH11-LIKE INTEGRAL MEMBRANE PROTEIN (AFU_ORTHOLOGUE AFUA_5G11245)
AOL_s00173g37.1	—	7	489	2	Candidate	PTH11-LIKE INTEGRAL MEMBRANE PROTEIN (AFU_ORTHOLOGUE AFUA_5G11245); Fungal rhodopsin domain
AOL_s00170g99.1	—	7	380	2	Candidate	PTH11-LIKE INTEGRAL MEMBRANE PROTEIN (AFU_ORTHOLOGUE AFUA_5G11245); Fungal rhodopsin domain
AOL_s00097g558.3	—	7	401	8	Candidate	G PROTEIN-COUPLED RECEPTOR 157-RELATED; Family A G protein-coupled receptor-like; G protein-coupled glucose receptor regulating Gpa2; G-protein coupled receptors family 2 profile 2.; G protein-coupled glucose receptor regulating Gpa2 C-term; GCR1-cAMP receptor family signature; Putative plant GPCR, GCR1, signature; G-protein coupled receptors family 1 profile.
AOL_s00081g15.1	—	7	451	2	Candidate	Fungal rhodopsin domain; PTH11-LIKE INTEGRAL MEMBRANE PROTEIN (AFU_ORTHOLOGUE AFUA_5G11245)
AOL_s00079g452.1	—	7	539	3	Candidate	G protein-coupled glucose receptor regulating Gpa2; Family A G protein-coupled receptor-like; G PROTEIN-COUPLED RECEPTOR 157-RELATED
AOL_s00109g8.1	—	7	401	2	Candidate	Fungal rhodopsin domain; PTH11-LIKE INTEGRAL MEMBRANE PROTEIN (AFU_ORTHOLOGUE AFUA_5G11245)
AOL_s00215g301.1	—	7	469	4	Candidate	G PROTEIN-COUPLED RECEPTOR 157-RELATED; Family A G protein-coupled receptor-like; G-protein coupled receptors family 2 profile 2.; Slime mold cyclic AMP receptor
AOL_s00043g686.1	—	7	380	2	Candidate	Fungal rhodopsin domain; PTH11-LIKE INTEGRAL MEMBRANE PROTEIN (AFU_ORTHOLOGUE AFUA_5G11245)
AOL_s00210g135.2	—	7	374	2	Candidate	Fungal rhodopsin domain; PTH11-LIKE INTEGRAL MEMBRANE PROTEIN (AFU_ORTHOLOGUE AFUA_5G11245)
AOL_s00188g284.1	—	7	435	2	Candidate	PTH11-LIKE INTEGRAL MEMBRANE PROTEIN (AFU_ORTHOLOGUE AFUA_5G11245); Fungal rhodopsin domain
AOL_s00076g450.1	—	7	418	2	Candidate	PTH11-LIKE INTEGRAL MEMBRANE PROTEIN (AFU_ORTHOLOGUE AFUA_5G11245); Fungal rhodopsin domain

Table S2. Mapping of prioritized receptor codes, loci and UniProt accessions.

Study code	Receptor alias	Sequence identifier	ATCC 24927 locus	UniProt accession
Grn1	AoGPR200	AOL_s00007g309.1	AOL_s00007g309	G1X200
Grn2	AoGPRPR8	AOL_s00176g42.1	AOL_s00176g42	G1XPR8
Grn3	AoGPR5K6	AOL_s00054g150.1	AOL_s00054g150	G1X5K6

The study codes connect the prioritized candidate protein sequences to the receptor aliases used in the manuscript.

S2. Targeted replacement and transformant verification

Table S3. Fungal strain and genotype classes.

Strain	Genotype / background	Selection / status	Use
WT	A. oligospora ATCC 24927	Parental wild type	Culture, trap and qRT-PCR assays
ΔAoGPR200	AoGPR200 replaced with HPH cassette	Hygromycin-resistant deletion class	Growth, trap and qRT-PCR assays
ΔAoGPR5K6	AoGPR5K6 replaced with HPH cassette	Hygromycin-resistant deletion class	Trap assay and diagnostic PCR
ΔAoGPRPR8	AoGPRPR8 replaced with HPH cassette	Hygromycin-resistant deletion class	Growth, trap and qRT-PCR assays
ΔAoGPR200C	AoGPR200 restored under OliC promoter in ΔAoGPR200	Nourseothricin-selected complemented class	Growth and trap assays
ΔAoGPRPR8C	AoGPRPR8 restored under OliC promoter in ΔAoGPRPR8	Nourseothricin-selected complemented class	Growth and trap assays

The table lists genotype classes. Molecular validation records are shown separately in Figures S1–S3. Gel-lane identities and growth-plate identities have not been assigned to sequencing-isolate identifiers.

Table S4. Oligonucleotides for construct generation, sgRNA design and qRT-PCR.

Use	Name	Sequence (5′–3′)
Complementation	R-PR8-F	CCATCACATCACAATCGATCCATGGGAATGTCAGGGGAT
Complementation	R-200-F	CCATCACATCACAATCGATCCATGGGTTTCGGTGCGG
Complementation	R-PR8-R	CGCCCTTGCTCACCATTGAGCCTCCCGATCCACCGCGCCACCCTTCTCT
Complementation	R-200-R	CGCCCTTGCTCACCATTGAGCCTCCCGATCCACCAAAGACTTCCTTCGTGGCT
Homology template / diagnostic PCR	GPR5K6P1	aattcggatcttccagagatCGAACCAGGAGATTACGG
Homology template / diagnostic PCR	GPR5K6P2	atccttctttGCGGCTCCGCTAATCATC
Homology template / diagnostic PCR	GPR5K6P3	gcggagccgcAAAGAAGGATTACCTCTAAAC
Homology template / diagnostic PCR	GPR5K6P4	catgatccgtCAGAAGATGATATTGAAGGAG
Homology template / diagnostic PCR	GPR5K6P5	tcatcttctgACGGATCATGAAAAATAATAGTGC
Homology template / diagnostic PCR	GPR5K6P6	ttcaactgccgttcgacgatGTTGGGTTCCCGAGTACC
Homology template / diagnostic PCR	GPR5K6P7	cgaaccaggagattacggg
Homology template / diagnostic PCR	GPR5K6P8	gttgggttcccgagtacc
Homology template / diagnostic PCR	GPR5K6P9	gggaatcccaacagccg
Homology template / diagnostic PCR	GPR5K6P10	ctcgtgacctcgtgaatgac
Homology template / diagnostic PCR	GPRPR8P1	aattcggatcttccagagataggagctcaccatctcatg
Homology template / diagnostic PCR	GPRPR8P2	atccttcttttttacttgttggaaaggaagg
Homology template / diagnostic PCR	GPRPR8P3	acaagtaaaaaaagaaggattacctctaaac
Homology template / diagnostic PCR	GPRPR8P4	gggggtacgccagaagatgatattgaaggag
Homology template / diagnostic PCR	GPRPR8P5	tcatcttctggcgtacccccaaatattc
Homology template / diagnostic PCR	GPRPR8P6	ttcaactgccgttcgacgatccctcccaatcttcgtatc
Homology template / diagnostic PCR	GPRPR8P7	aggagctcaccatctcatg
Homology template / diagnostic PCR	GPRPR8P8	cccaatcttcgtatcttccgt
Homology template / diagnostic PCR	GPRPR8P9	gtcgggtagcagcgaag
Homology template / diagnostic PCR	GPRPR8P10	agttgtgatatgaacggcc
Homology template / diagnostic PCR	GPR200P1	aattcggatcttccagagattagtctccacaatattgtg
Homology template / diagnostic PCR	GPR200P2	atccttctttcttgaatttttggttgacttatc
Homology template / diagnostic PCR	GPR200P3	aaaattcaagaaagaaggattacctctaaac
Homology template / diagnostic PCR	GPR200P4	cagtgactttcagaagatgatattgaaggag
Homology template / diagnostic PCR	GPR200P5	tcatcttctgaaagtcactgcagcagcc
Homology template / diagnostic PCR	GPR200P6	ttcaactgccgttcgacgatctatctaaccgaaagaccaaagg
Homology template / diagnostic PCR	GPR200P7	ctccacaatattgtggggact
Homology template / diagnostic PCR	GPR200P8	caaagggaacccctgc

Use	Name	Sequence (5′–3′)
Homology template / diagnostic PCR	GPR200P9	ggaggatcttaaggagctcg
Homology template / diagnostic PCR	GPR200P10	cgccacgtcaagtgag
sgRNA	GPR200	GTCAAGCTCACCAAGAACAGAGG
sgRNA	GPRPR8	GGCATGAGTACATGGTACCGTGG
sgRNA	GPR5K6	TGTGCCATACTTCGTCACGGCGG
qRT-PCR: Tub (tubulin)	tubulin-F	CCACCTTCGTCGGTAACTCG
qRT-PCR: Tub (tubulin)	tubulin-R	TGCTCCTCATCGGCATAAG
qRT-PCR: AoGPR200	200F	TGTGGATGAGTGCTGTTATGT
qRT-PCR: AoGPR200	200R	GAAGTGCTCTAGCCGATGTT
qRT-PCR: AoGPRPR8	PR8F	CGGATGACGCTGATGAAGAT
qRT-PCR: AoGPRPR8	PR8R	TCGAGAGCTGACGAGACTATAA

Construct-generation and sgRNA sequences retain the original letter case. Guide and PAM boundaries were not distinguished in the supplied sgRNA entries; the complete strings are shown. qRT-PCR assay performance is reported in Table S6.

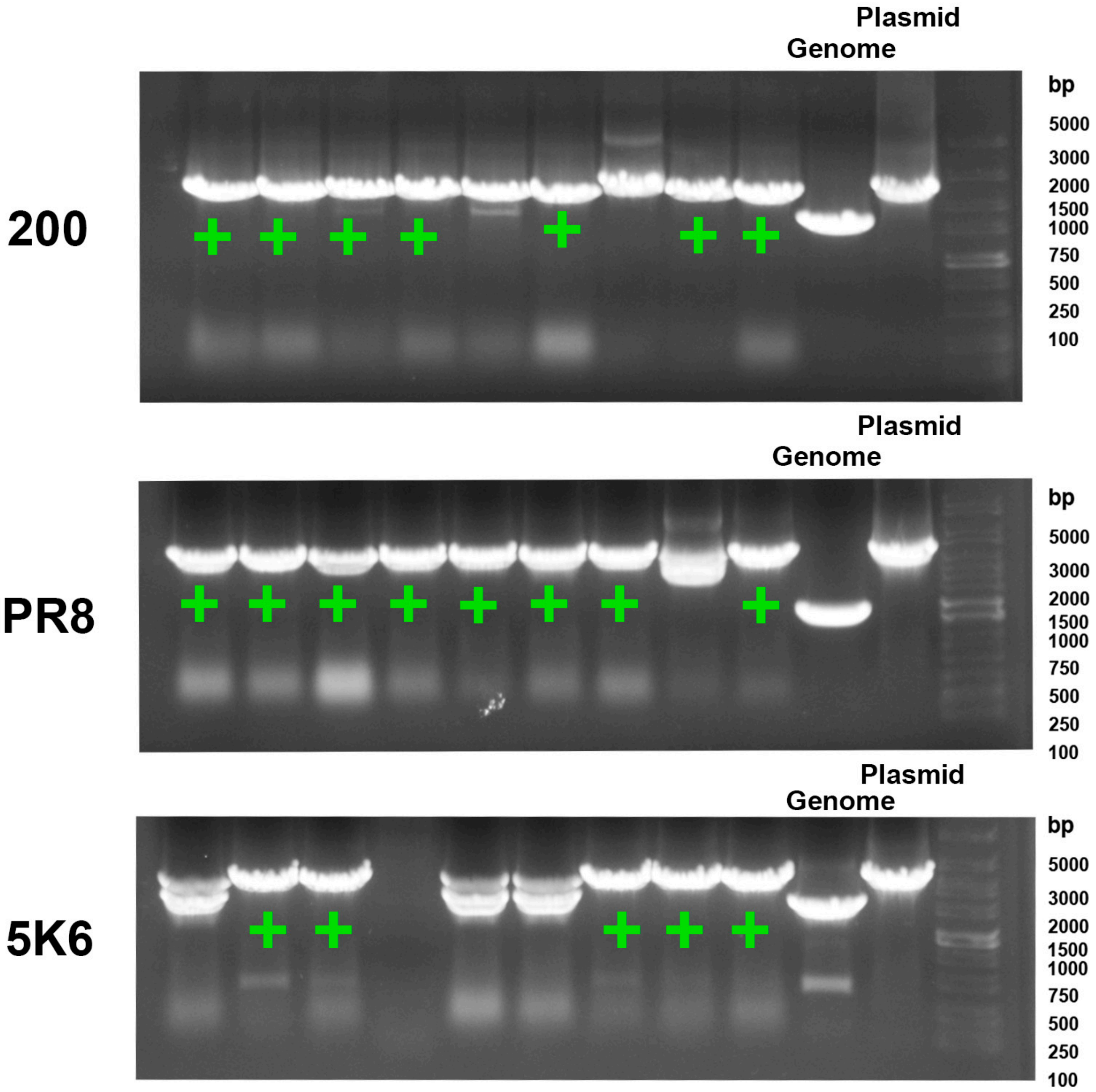

Figure S1. Full supplied diagnostic PCR gel panels for Grn1 (source label 200), Grn2 (PR8) and Grn3 (5K6), arranged from top to bottom. The three source panels, ladder labels and green + marks are retained in their supplied state, with no additional cropping, contrast adjustment or lane rearrangement. Genome and Plasmid denote the controls labelled in the source image; green + symbols reproduce the marked candidate selections. Individual candidate lane numbers were not recorded and are not assigned to the sequenced isolates. These diagnostic gels and the sequence-level junction evidence in Figures S2–S3 provide complementary verification.

S2.1. Bidirectional Sanger sequencing and junction assessment

Forward (F) and reverse (R) AB1 reads were analyzed for three candidate isolates at each locus and the corresponding wild-type (WT) PCR controls (16 reads in total). Grn1 denotes AoGPR200 (AOL_s00007g309), and Grn2 denotes AoGPRPR8 (AOL_s00176g42). Reads were aligned independently to the supplied WT fragments and homologous-recombination (HR) donor designs using local affine-gap alignment (match 2, mismatch −3, gap opening −6, gap extension −1). R reads were reverse-complemented for alignment, with source base positions and quality scores retained.

Both donor designs contained a 2121-bp HPH cassette at reference positions 5958–8078. Junction support required exact matching source calls with $Q \geq 20$ at all 30 reference positions on each side of the designed boundary. Q30 counts summarize the two complete reads per candidate. Cassette coverage counts design positions with at least one aligned Q20 source base; it is not a measurement of physical insert length.

S2.2. Junction results

Grn1-4 and Grn1-6 showed high-quality sequence spanning both expected cassette–flank junctions. Grn1-2 showed the expected downstream junction but an unresolved upstream boundary. All three Grn2 candidates showed supported downstream junctions; their upstream boundaries remained within low-quality initial forward calls. Grn1-4 and Grn2-9 were selected for Figure S2 because they had the highest combined forward-plus-reverse Q30 counts at their respective loci. Figure S3 retains all six candidates.

Shared sequence differences from the HR donor design occurred in the cassette terminal regions. A six-base difference near the annotated HygR start had repeat-equivalent gap placements that preserved the start codon and was not interpreted as coding-sequence disruption. The low-quality central overlap did not establish a complete high-quality cassette consensus. The data support the junction structures specified above; full cassette identity, copy number and unique genomic integration were not established by these reads.

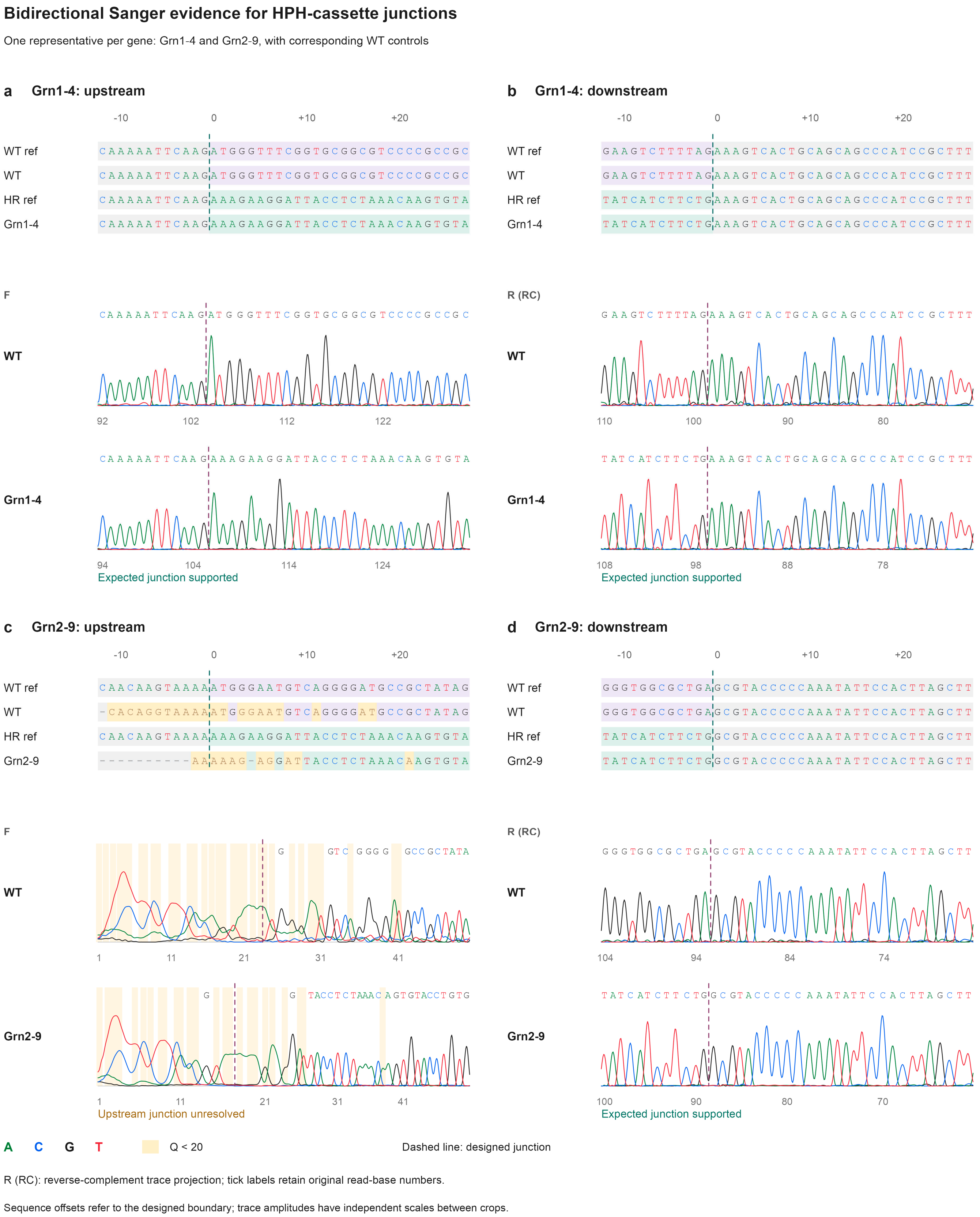

Figure S2. Representative Sanger evidence for HPH-cassette junctions. (a,b) Grn1-4 upstream and downstream junctions; both are supported. (c,d) Grn2-9 upstream and downstream junctions; only the downstream junction is supported. WT ref, wild-type reference; HR ref, donor design. Dashed lines mark the designed boundaries. Offsets are relative to the first cassette or downstream-flank base. Replaced WT and cassette sequences are juxtaposed at their designed coordinates, without implying homology. A, green; C, blue; G, black; T, red; amber, Q < 20. Low-quality letters in initial Grn2 trace labels are omitted for legibility; their calls remain in the sequence display. R (RC) indicates reverse-complement trace projection; source read positions descend left to right. Trace amplitudes use independent linear scales between crops; no smoothing or base-call correction was applied.

(a) Junction support and read quality

Locus	Candidate	Upstream / F	Downstream / R	F+R Q30 bases	HPH Q20 coverage
Grn1	Grn1-2	Unresolved	Supported	1005	1023 / 2121
Grn1	Grn1-4	Supported	Supported	1826	1865 / 2121
Grn1	Grn1-6	Supported	Supported	1795	1845 / 2121
Grn2	Grn2-4	Unresolved	Supported	1510	1746 / 2121
Grn2	Grn2-9	Unresolved	Supported	1825	1944 / 2121
Grn2	Grn2-8	Unresolved	Supported	1791	1890 / 2121

Grn1-4 and Grn2-9 are the representative isolates; all six candidates are retained.

b Per-read Q20 coverage of HPH design coordinates

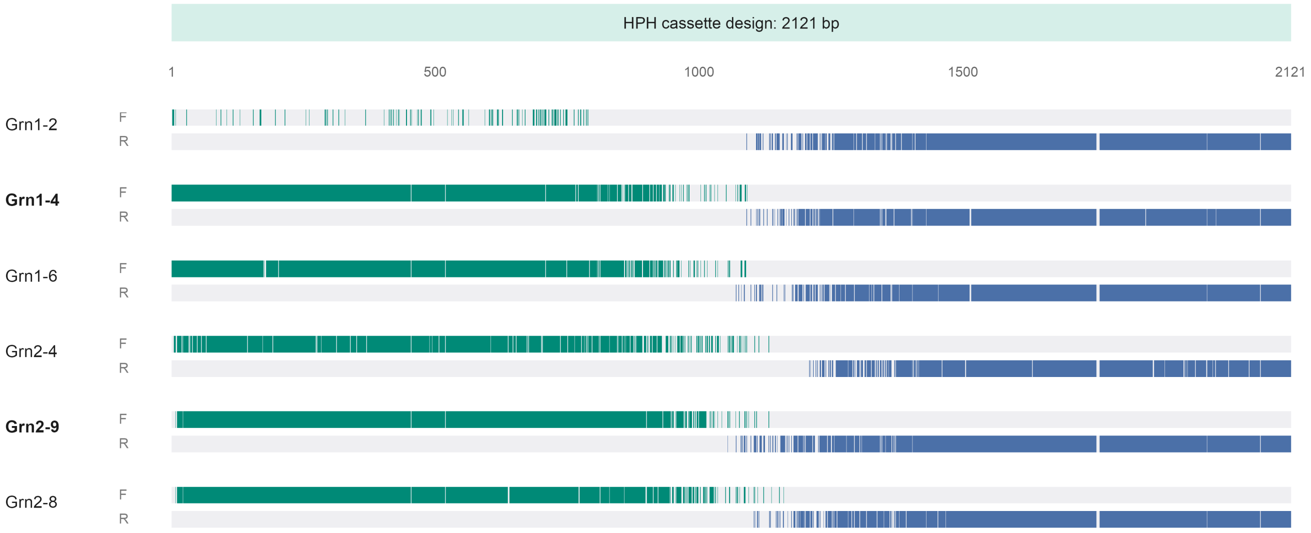

Q20 aligned source-base coverage; gray includes low-quality positions and design-coordinate deletions.

Reference-coordinate coverage does not establish physical insert length or a complete cassette consensus.

Figure S3. All-candidate junction evidence and HPH design-coordinate coverage. (a) An editable three-line table reports junction support, combined F+R Q30 base counts and Q20 cassette coverage. Supported denotes 30/30 exact Q20 calls on each side of the boundary; unresolved denotes insufficient evidence. (b) Aligned Q20 source-base coverage of the 2121-bp cassette design: forward, green; reverse, blue; gray, no aligned Q20 source base, including design-coordinate deletions. Bold isolate labels in panel b identify the representatives in Figure S2. Coverage counts reference positions and does not establish physical cassette length or a full-cassette consensus.

S3. Basal growth: plate-level statistical analysis

Colony diameter was measured daily on the same five independent culture plates per strain. There was no pairing between strains or batch structure. Each plate-level area under the curve (AUC) was calculated by the trapezoidal rule over days 1–5. Day 6 was excluded from the primary AUC because expansion was limited by the plate edge, and is retained in the complete source measurements (Data S3). The three deletion isolates for each gene were analysed separately.

Eight non-WT strains were compared with the common WT group using two-sided Mann–Whitney rank statistics. Exact P values were obtained by enumerating all 252 allocations of the ten plate labels per comparison, including tied ranks. Holm adjustment was applied jointly to all eight comparisons. Welch comparisons and AUCs including day 6 were retained as sensitivity analyses. All 45 plates were retained.

WT AUC was 9.53 ± 1.13 cm·day (mean ± SD). Non-WT means were 7.43–8.09 cm·day, descriptively 15.1–22.0% below WT, but no individual comparison reached significance after Holm correction (adjusted P = 0.0635–0.1429). The omnibus Kruskal–Wallis statistic was H(8) = 14.219, with permutation P = 0.0587 (99,999 permutations). These results do not establish growth equivalence.

Table S5. Plate-level growth AUC statistics and sensitivity analyses.

(A) Primary AUC, days 1–5

Strain	n	AUC mean ± SD (cm·day)	Difference vs WT	Exact P	Holm P
WT	5	9.53 ± 1.13	—	—	—
ΔGrn2c	5	7.58 ± 0.26	-1.95	0.0079	0.0635
ΔGrn2-1	5	8.00 ± 0.38	-1.53	0.0079	0.0635
ΔGrn2-2	5	7.43 ± 0.94	-2.10	0.0079	0.0635
ΔGrn2-3	5	7.68 ± 0.63	-1.85	0.0079	0.0635
ΔGrn1c	5	7.85 ± 0.96	-1.68	0.0794	0.1429
ΔGrn1-1	5	7.62 ± 0.47	-1.91	0.0079	0.0635
ΔGrn1-2	5	8.09 ± 0.87	-1.44	0.0714	0.1429
ΔGrn1-3	5	7.72 ± 0.69	-1.81	0.0079	0.0635

n denotes independent plates, not daily measurements. Difference is the mean AUC difference in cm·day. Growth-isolate names are retained from their source records and are not assigned to Sanger isolate names.

(B) Sensitivity analyses: Holm-adjusted P values

Strain	Welch, days 1–5	Mann–Whitney, days 1–6	Welch, days 1–6
ΔGrn2c	0.1084	0.0635	0.0964
ΔGrn2-1	0.1084	0.0635	0.0964
ΔGrn2-2	0.1051	0.0635	0.0964
ΔGrn2-3	0.1084	0.0635	0.0964
ΔGrn1c	0.1084	0.1270	0.0964
ΔGrn1-1	0.1084	0.0635	0.0401
ΔGrn1-2	0.1084	0.1270	0.0964
ΔGrn1-3	0.1084	0.0635	0.0964

Each sensitivity family contains the same eight comparisons with WT. Day-6 AUCs are plate-edge-limited. All primary day 1–5 comparisons remain non-significant. The day 1–6 Welch sensitivity comparison for ΔGrn1-1 gives adjusted P = 0.0401; this plate-edge-limited sensitivity result is not substituted for the primary analysis.

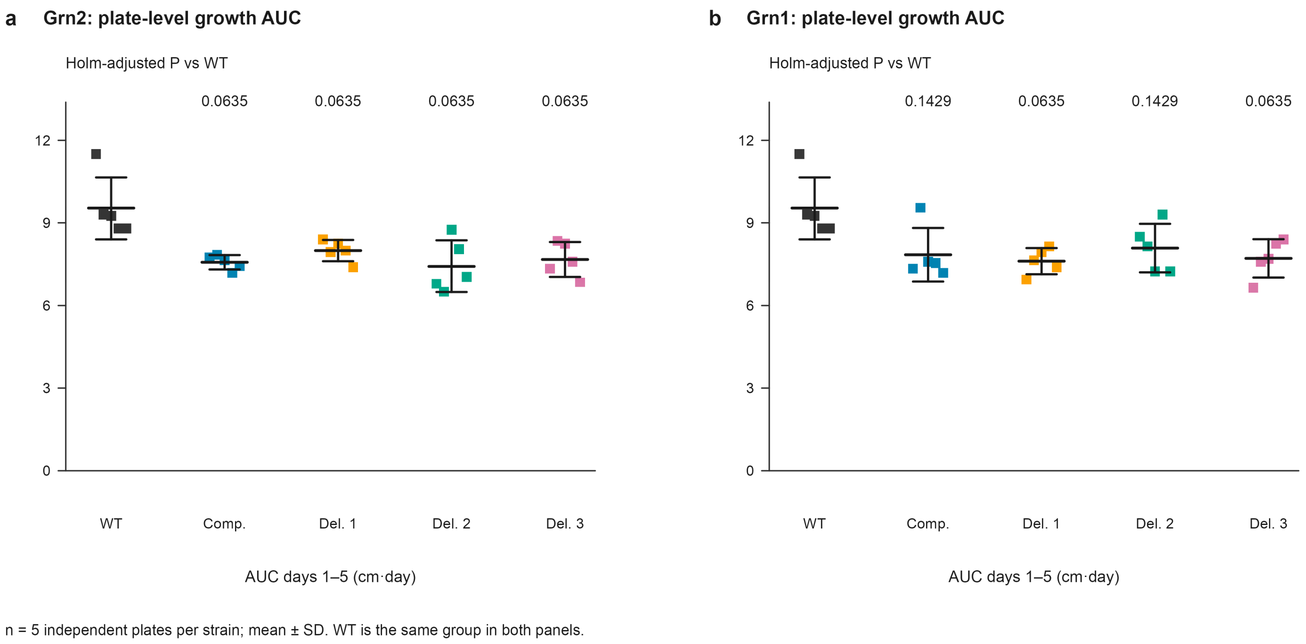

Figure S4. Plate-level growth AUC over days 1–5 for (a) Grn2 and (b) Grn1 strains. Each square is one independent plate; horizontal marks and error bars show mean ± SD (n = 5 per strain). WT is the same five-plate group shown in both panels. Comp., complemented strain; Del. 1–3, the three independent deletion isolates for the indicated gene. Values above non-WT groups are two-sided exact Mann–Whitney P values adjusted jointly across all eight comparisons by Holm's method. The complete numerical results and sensitivity analyses are in Table S5; the complete day 1–6 measurements are in Data S3.

S4. Receptor transcript assays

Table S6. RT-qPCR primers and assay performance.

Target	Primer	Sequence (5′–3′)	Tm (°C)	Amplicon (bp)	Efficiency (%)	R^2
PR8 (Grn2)	R8F	CGGATGACGCTGATGAAGAT	58.4	111	99.39	0.9999
PR8 (Grn2)	R8R	TCGAGAGCTGACGAGACTATAA	54.9	111	99.39	0.9999
200 (Grn1)	20F	TGTGGATGAGTGCTGTTATGT	53.4	117	99.14	0.9999
200 (Grn1)	20R	GAAGTGCTCTAGCCGATGTT	54.6	117	99.14	0.9999
Tub	TF	CCACCTTCGTCGGTAACTCG	60.4	110	109.04	0.9986
Tub	TR	TGCTCCTCATCGGCATAAG	56.8	110	109.04	0.9986
5K6 (Grn3)	K6F	CCTCAATCTCACACCGAGAAAG	62.0	101	99.96	0.9941
5K6 (Grn3)	K6R	GCCCAACTTCCACAATTCTAATG	62.0	101	99.96	0.9941

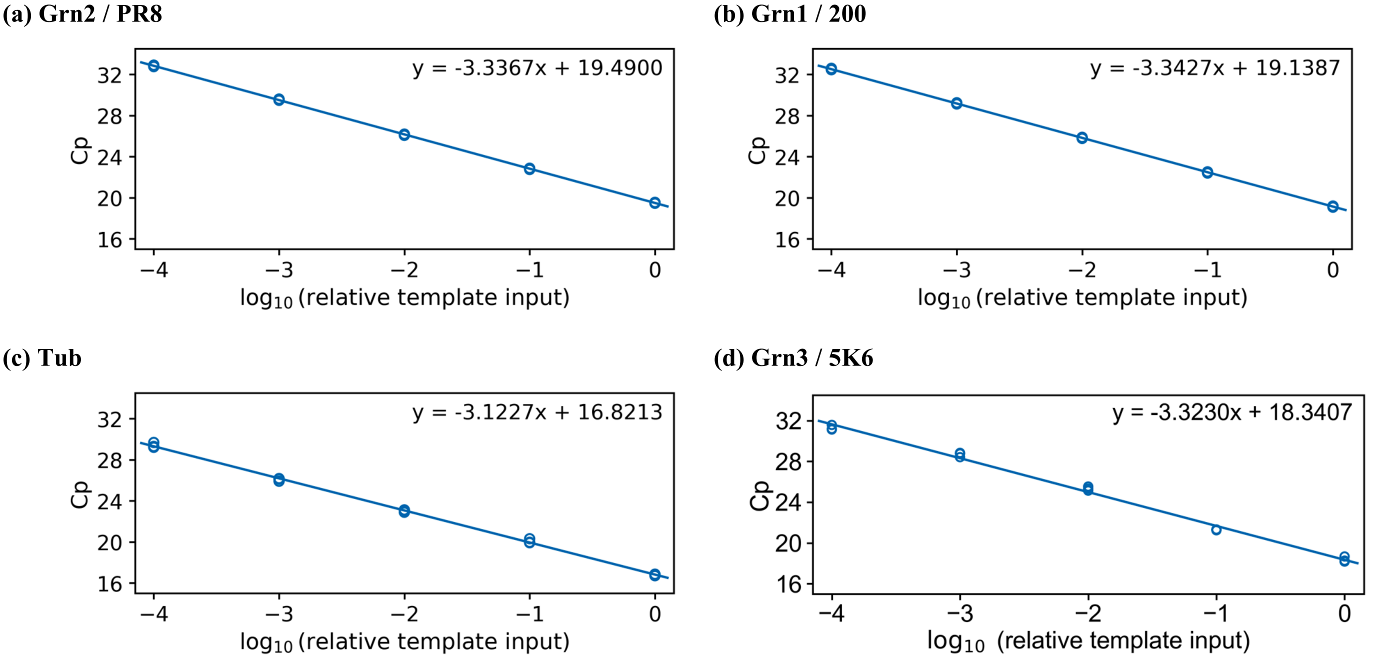

Figure S5. Standard curves for (a) Grn2, (b) Grn1, (c) Tub and (d) Grn3 from the separate standard-dilution workbook (Data S2). Symbols show mean Ct of the three technical wells at each dilution. Regression equations are shown in the panels; Table S6 reports efficiency and R^2 from all 15 wells per assay. The x-axis is $\log_{10}$(relative template input). These standard-dilution measurements are distinct from the biological expression workbook.

S4.1. RT-qPCR expression analysis

The expression analysis uses the supplied qRTPCR raw-data workbook exclusively: 96 sample–assay triplicates, comprising 288 technical reactions from 24 biological sample–time units. The design includes WT, ΔGrn1, ΔGrn2 and ΔGrn3 at 0 h and 10 h, with three biological samples per genotype–time condition and three technical reactions per sample–assay. Source zero codes indicate non-detection and are retained in the raw table but excluded from Ct arithmetic. All 54 ND reactions occurred in the corresponding deletion-target assays; all Tub assays were detected.

Detected technical triplicates were averaged within each biological sample. ΔCt equals target mean Ct minus the matched Tub mean Ct. ΔΔCt subtracts the gene-specific mean ΔCt of the three WT 0 h samples; relative expression is $2^{(-\Delta\Delta Ct)}$, assuming an amplification factor of 2 per cycle for target and reference. The WT 0 h geometric mean is calibrated to 1; individual baseline values are retained. Fourteen detected triplicates met the technical review flag (Ct SD > 0.5 or range > 1 cycle); no reactions were excluded.

Biological samples were matched by replicate number for paired analyses. Two-sided paired t tests were applied to −ΔCt contrasts, with 3 pairs and 2 degrees of freedom. The nine time, twelve same-time genotype-versus-WT, and six genotype-by-time contrasts form 27 comparisons. The displayed figure and the global adjusted P column use Holm adjustment across all 27; family-specific adjustments are additionally recorded in Data S2 and are not substituted for the displayed correction. Exact sign-flip sensitivity P values are supplied because the normality assumption cannot be reliably checked with $n = 3$.

WT paired geometric-mean 10 h/0 h ratios were 2.567 for Grn1, 1.716 for Grn2 and 9.759 for Grn3. Their global Holm-adjusted P values were 0.310, 0.797 and 0.092, respectively. None reached 0.05 under this global correction. Corresponding target transcripts were ND in all 9 technical reactions per deletion-target/time condition. No numerical fold change or t test was assigned to ND targets.

Table S7. Source Ct measurements and biological-replicate expression analyses.

(A) Complete source Ct measurements

Excel row	Source sample	Assay	Time	Ct1	Ct2	Ct3
2	WT1	Tub	0h	18.79	18.79	18.09
3	WT2	Tub	0h	18.49	18.06	18.55
4	WT3	Tub	0h	18.44	18.89	18.35
5	WT1	Grn1	0h	25.71	25.08	25.08
6	WT2	Grn1	0h	25.99	25.37	25.37
7	WT3	Grn1	0h	25.81	25.95	25.99
8	WT1	Grn2	0h	25.51	24.75	24.17
9	WT2	Grn2	0h	25.55	25.12	24.85
10	WT3	Grn2	0h	25.81	24.22	24.99
11	WT1	Grn3	0h	26.02	26.94	26.11
12	WT2	Grn3	0h	26.24	26.24	27.3
13	WT3	Grn3	0h	27.49	27.17	27.92
14	WT1	Tub	10h	18.31	18.21	18.86
15	WT2	Tub	10h	18.15	18.96	17.91
16	WT3	Tub	10h	18.97	17.95	18.88
17	WT1	Grn1	10h	24.06	24.99	23.15
18	WT2	Grn1	10h	24.11	24.94	24.05
19	WT3	Grn1	10h	24.26	24.39	23.91
20	WT1	Grn2	10h	24.26	24.17	24.8
21	WT2	Grn2	10h	23.09	23.56	24.9
22	WT3	Grn2	10h	24.78	23.91	24.24
23	WT1	Grn3	10h	23.28	23.19	23.46
24	WT2	Grn3	10h	23.35	23.58	23.08
25	WT3	Grn3	10h	23.97	23.99	23.7
26	ΔGrn1-1	Tub	0h	17.81	17.71	17.97
27	ΔGrn1-2	Tub	0h	17.93	17.52	17.51
28	ΔGrn1-3	Tub	0h	17.69	17.91	17.99
29	ΔGrn1-1	Grn1	0h	0	0	0
30	ΔGrn1-2	Grn1	0h	0	0	0
31	ΔGrn1-3	Grn1	0h	0	0	0
32	ΔGrn1-1	Grn2	0h	25.1	26.47	26.38
33	ΔGrn1-2	Grn2	0h	26.7	26.69	25.94
34	ΔGrn1-3	Grn2	0h	26.31	26.61	26.44
35	ΔGrn1-1	Grn3	0h	26.17	26.32	26.09
36	ΔGrn1-2	Grn3	0h	26.67	26.42	26.16
37	ΔGrn1-3	Grn3	0h	26.47	26.43	26.25
38	ΔGrn1-1	Tub	10h	17.74	18.13	17.94
39	ΔGrn1-2	Tub	10h	18.47	18.36	18.35
40	ΔGrn1-3	Tub	10h	18.02	17.97	17.85

Excel row	Source sample	Assay	Time	Ct1	Ct2	Ct3
41	ΔGrn1-1	Grn1	10h	0	0	0
42	ΔGrn1-2	Grn1	10h	0	0	0
43	ΔGrn1-3	Grn1	10h	0	0	0
44	ΔGrn1-1	Grn2	10h	22.23	23.34	23.04
45	ΔGrn1-2	Grn2	10h	23.54	23.04	23.7
46	ΔGrn1-3	Grn2	10h	23.1	23.12	23.75
47	ΔGrn1-1	Grn3	10h	21.81	21.27	21.06
48	ΔGrn1-2	Grn3	10h	22.51	22.24	22.41
49	ΔGrn1-3	Grn3	10h	21.43	21.27	21.03
50	ΔGrn2-1	Tub	0h	15.62	15.86	15.95
51	ΔGrn2-2	Tub	0h	15.15	15.93	15.49
52	ΔGrn2-3	Tub	0h	15.26	15.46	15.98
53	ΔGrn2-1	Grn1	0h	23.49	23.33	23.63
54	ΔGrn2-2	Grn1	0h	23.24	23.08	23.13
55	ΔGrn2-3	Grn1	0h	23.13	23.15	23.14
56	ΔGrn2-1	Grn2	0h	0	0	0
57	ΔGrn2-2	Grn2	0h	0	0	0
58	ΔGrn2-3	Grn2	0h	0	0	0
59	ΔGrn2-1	Grn3	0h	25.28	24.36	24.69
60	ΔGrn2-2	Grn3	0h	25.79	24.95	25.34
61	ΔGrn2-3	Grn3	0h	24.34	24.38	24.08
62	ΔGrn2-1	Tub	10h	16.17	16.28	16.18
63	ΔGrn2-2	Tub	10h	16.09	16.12	16.46
64	ΔGrn2-3	Tub	10h	16.21	16.36	16.5
65	ΔGrn2-1	Grn1	10h	21.38	20.08	21.6
66	ΔGrn2-2	Grn1	10h	21.26	20.16	21.75
67	ΔGrn2-3	Grn1	10h	21.84	20.12	20.55
68	ΔGrn2-1	Grn2	10h	0	0	0
69	ΔGrn2-2	Grn2	10h	0	0	0
70	ΔGrn2-3	Grn2	10h	0	0	0
71	ΔGrn2-1	Grn3	10h	24.61	24.5	23.37
72	ΔGrn2-2	Grn3	10h	23.42	23.74	23.97
73	ΔGrn2-3	Grn3	10h	24.24	23.74	23.93
74	ΔGrn3-1	Tub	0h	18.75	18.04	18.25
75	ΔGrn3-2	Tub	0h	18.71	18.9	18.51
76	ΔGrn3-3	Tub	0h	18.53	18.11	18.45
77	ΔGrn3-1	Grn1	0h	22.89	22.59	22.68
78	ΔGrn3-2	Grn1	0h	22.79	22.5	22.09
79	ΔGrn3-3	Grn1	0h	22.54	22.59	22.75
80	ΔGrn3-1	Grn2	0h	24.43	24.13	24.28
81	ΔGrn3-2	Grn2	0h	24.36	24.65	24.57
82	ΔGrn3-3	Grn2	0h	24.36	24.99	24.61
83	ΔGrn3-1	Grn3	0h	0	0	0
84	ΔGrn3-2	Grn3	0h	0	0	0
85	ΔGrn3-3	Grn3	0h	0	0	0
86	ΔGrn3-1	Tub	10h	18.07	17.48	17.54
87	ΔGrn3-2	Tub	10h	17.75	17.04	17.64
88	ΔGrn3-3	Tub	10h	17.42	17.65	17.24
89	ΔGrn3-1	Grn1	10h	20.24	20.1	20.15
90	ΔGrn3-2	Grn1	10h	20.25	20.16	20.19
91	ΔGrn3-3	Grn1	10h	20.29	20.17	20.9
92	ΔGrn3-1	Grn2	10h	21.08	21.52	21.41
93	ΔGrn3-2	Grn2	10h	21.98	21.11	21.4
94	ΔGrn3-3	Grn2	10h	21.97	21.87	21.82
95	ΔGrn3-1	Grn3	10h	0	0	0
96	ΔGrn3-2	Grn3	10h	0	0	0
97	ΔGrn3-3	Grn3	10h	0	0	0

All 96 source rows are reproduced in workbook order. Excel row identifies the exact worksheet row. A source value of 0 is the code for ND, not a measured Ct of zero. Source workbooks, all technical values and QC flags are provided in Data S2.

Table S7 (continued).

(B) Biological-replicate expression summaries

Target	Genotype	Time	Quantified n/3	ΔCt mean ± SD	Relative geometric mean	Relative mean ± SD	Detected wells / 9
Grn1	WT	0 h	3/3	7.100 ± 0.326	1.000	1.018 ± 0.240	9/9
Grn1	WT	10 h	3/3	5.740 ± 0.248	2.567	2.591 ± 0.422	9/9
Grn1	ΔGrn1	0 h	0/3	ND	ND	ND	0/9
Grn1	ΔGrn1	10 h	0/3	ND	ND	ND	0/9
Grn1	ΔGrn2	0 h	3/3	7.624 ± 0.050	0.695	0.696 ± 0.024	9/9
Grn1	ΔGrn2	10 h	3/3	4.708 ± 0.198	5.250	5.283 ± 0.749	9/9
Grn1	ΔGrn3	0 h	3/3	4.130 ± 0.331	7.835	7.979 ± 1.918	9/9
Grn1	ΔGrn3	10 h	3/3	2.736 ± 0.275	20.598	20.848 ± 3.934	9/9
Grn2	WT	0 h	3/3	6.502 ± 0.281	1.000	1.012 ± 0.191	9/9
Grn2	WT	10 h	3/3	5.723 ± 0.220	1.716	1.729 ± 0.261	9/9
Grn2	ΔGrn1	0 h	3/3	8.511 ± 0.326	0.248	0.253 ± 0.059	9/9
Grn2	ΔGrn1	10 h	3/3	5.114 ± 0.233	2.617	2.639 ± 0.408	9/9
Grn2	ΔGrn2	0 h	0/3	ND	ND	ND	0/9
Grn2	ΔGrn2	10 h	0/3	ND	ND	ND	0/9
Grn2	ΔGrn3	0 h	3/3	6.014 ± 0.245	1.402	1.415 ± 0.231	9/9
Grn2	ΔGrn3	10 h	3/3	4.037 ± 0.405	5.523	5.669 ± 1.564	9/9
Grn3	WT	0 h	3/3	8.331 ± 0.590	1.000	1.055 ± 0.401	9/9
Grn3	WT	10 h	3/3	5.044 ± 0.222	9.759	9.835 ± 1.474	9/9
Grn3	ΔGrn1	0 h	3/3	8.549 ± 0.202	0.860	0.865 ± 0.119	9/9
Grn3	ΔGrn1	10 h	3/3	3.578 ± 0.367	26.971	27.533 ± 6.528	9/9
Grn3	ΔGrn2	0 h	3/3	9.168 ± 0.594	0.560	0.590 ± 0.216	9/9
Grn3	ΔGrn2	10 h	3/3	7.683 ± 0.239	1.567	1.581 ± 0.253	9/9
Grn3	ΔGrn3	0 h	0/3	ND	ND	ND	0/9
Grn3	ΔGrn3	10 h	0/3	ND	ND	ND	0/9

Each condition contains three biological samples, each measured in technical triplicate. Relative expression is $2^{(-\Delta\Delta Ct)}$, calibrated to the gene-specific geometric mean of WT 0 h. Arithmetic mean ± SD describes the plotted biological values; the geometric mean and paired fold contrasts are different estimators. ND targets are not assigned zero expression.

Table S7 (continued).

(C) Complete paired contrasts and global multiplicity correction

Target	Comparison	Pairs	Paired fold [95% CI]	Raw P	Holm P, 27
Grn1	WT:10h / 0h	3	2.567 [1.390, 4.740]	0.0221	0.3095
Grn1	ΔGrn2:10h / 0h	3	7.551 [5.763, 9.894]	0.0010	0.0251
Grn1	ΔGrn3:10h / 0h	3	2.629 [1.198, 5.771]	0.0339	0.4406
Grn1	ΔGrn2 / WT at 0h	3	0.695 [0.365, 1.323]	0.1355	0.7969
Grn1	ΔGrn2 / WT at 10h	3	2.045 [1.428, 2.929]	0.0133	0.2000
Grn1	ΔGrn2 induction / WT induction	3	2.942 [2.043, 4.236]	0.0061	0.1222
Grn1	ΔGrn3 / WT at 0h	3	7.835 [2.994, 20.503]	0.0116	0.1854
Grn1	ΔGrn3 / WT at 10h	3	8.025 [4.135, 15.572]	0.0054	0.1140
Grn1	ΔGrn3 induction / WT induction	3	1.024 [0.322, 3.256]	0.9373	1.0000
Grn2	WT:10h / 0h	3	1.716 [0.728, 4.045]	0.1135	0.7969
Grn2	ΔGrn1:10h / 0h	3	10.532 [6.157, 18.016]	0.0028	0.0671
Grn2	ΔGrn3:10h / 0h	3	3.939 [2.457, 6.314]	0.0063	0.1222
Grn2	ΔGrn1 / WT at 0h	3	0.248 [0.201, 0.307]	0.0013	0.0315
Grn2	ΔGrn1 / WT at 10h	3	1.525 [0.820, 2.836]	0.0996	0.7969
Grn2	ΔGrn1 induction / WT induction	3	6.138 [3.930, 9.587]	0.0032	0.0747
Grn2	ΔGrn3 / WT at 0h	3	1.402 [0.658, 2.990]	0.1946	0.7969
Grn2	ΔGrn3 / WT at 10h	3	3.219 [1.245, 8.327]	0.0339	0.4406
Grn2	ΔGrn3 induction / WT induction	3	2.296 [0.633, 8.321]	0.1089	0.7969
Grn3	WT:10h / 0h	3	9.759 [5.176, 18.399]	0.0042	0.0915
Grn3	ΔGrn1:10h / 0h	3	31.366 [21.074, 46.683]	0.0007	0.0194
Grn3	ΔGrn2:10h / 0h	3	2.798 [0.769, 10.186]	0.0756	0.6882
Grn3	ΔGrn1 / WT at 0h	3	0.860 [0.319, 2.317]	0.5796	1.0000
Grn3	ΔGrn1 / WT at 10h	3	2.764 [1.176, 6.493]	0.0361	0.4406
Grn3	ΔGrn1 induction / WT induction	3	3.214 [2.100, 4.920]	0.0071	0.1280
Grn3	ΔGrn2 / WT at 0h	3	0.560 [0.103, 3.031]	0.2776	0.8329
Grn3	ΔGrn2 / WT at 10h	3	0.161 [0.080, 0.324]	0.0079	0.1336
Grn3	ΔGrn2 induction / WT induction	3	0.287 [0.065, 1.270]	0.0688	0.6882

Time contrasts are 10 h / 0 h; genotype contrasts compare the indicated mutant with WT at the same time; interaction contrasts compare their time responses. Confidence intervals are pointwise 95% t intervals after back-transformation, not simultaneous multiplicity-adjusted intervals. Global Holm P values control the stated family of 27 comparisons. Family-specific Holm and exact sign-flip sensitivity results are retained in Data S2. All paired n = 3; technical wells do not increase n.

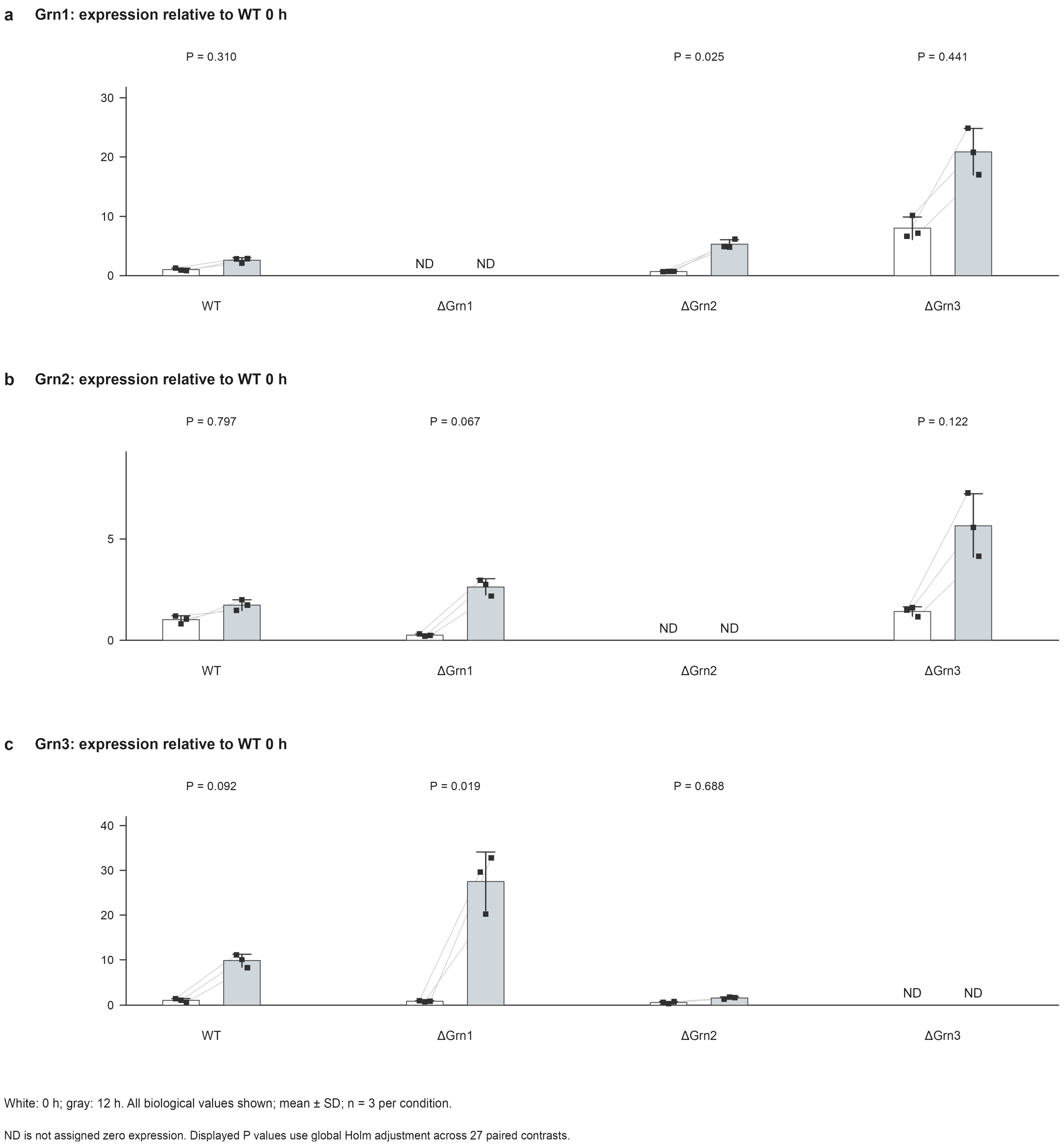

Figure S6. Relative transcript abundance of (a) Grn1, (b) Grn2 and (c) Grn3 in WT and deletion genotypes, recalculated from the source Ct workbook in Data S2. White bars, 0 h; gray bars, 10 h. Squares show all three biological samples per condition; bars and error bars show arithmetic mean ± SD. Gray lines connect matched replicate numbers. Relative expression is 2^(−ΔΔCt), with the gene-specific WT 0 h geometric mean set to 1 and an amplification-factor-of-2 assumption. ND indicates non-detection in all nine technical reactions and is not treated as zero expression. The plotted time-comparison P values come from paired t tests on −ΔCt with global Holm adjustment across all 27 paired contrasts. Y-axis scales differ among panels.

S5. BRET assay controls

(a) Gin3 positive control

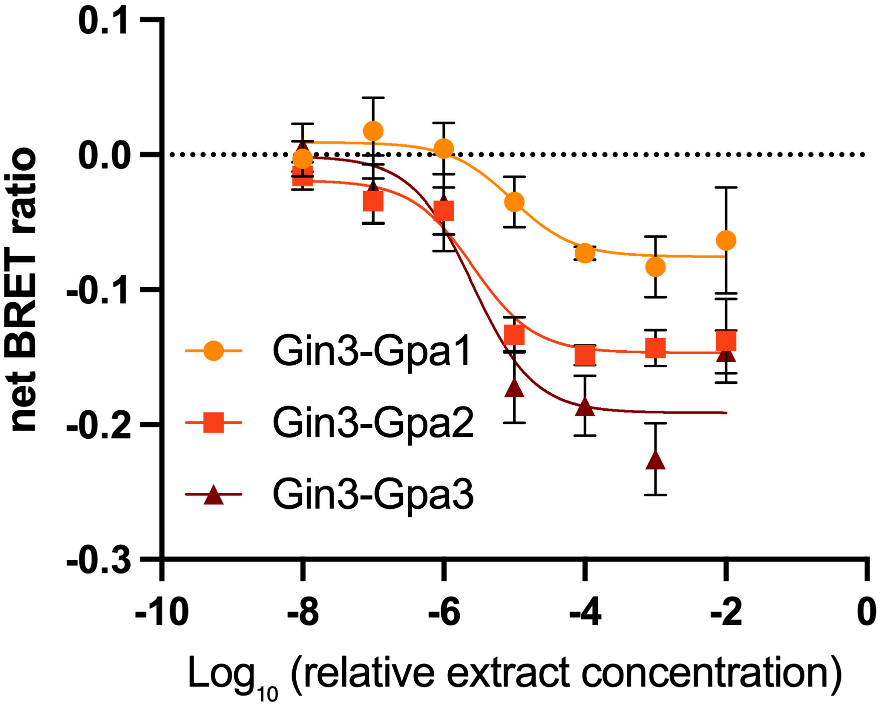

(b) Receptor-free negative control

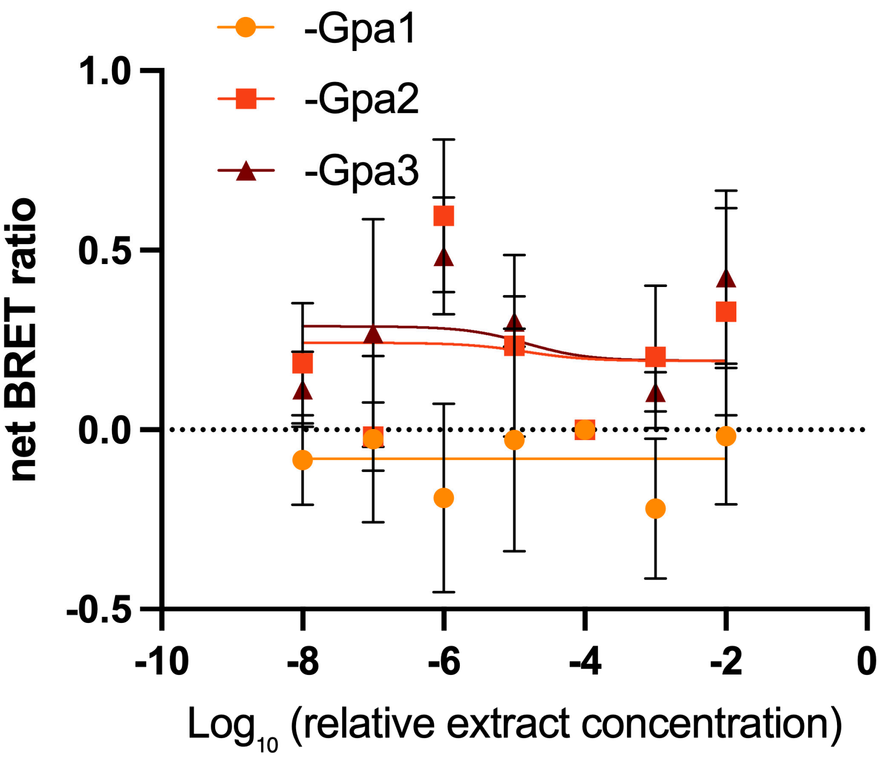

Figure S7. BRET positive and receptor-free negative controls. (a) Gin3 coexpressed with the G-protein sensor containing Gpa1, Gpa2 or Gpa3. (b) The corresponding negative-control systems express Gα, Gβ and Gγ without a receptor. Each condition was tested in three independent experiments with two technical replicates per experiment. The x-axis denotes $\log_{10}$(relative nematode-extract concentration), not a molar concentration. Symbols show means and error bars denote SD. Curves show three-parameter log(agonist)-versus-response fits generated in GraphPad Prism. Y-axis scales differ between panels.

S6. Public transcriptome resource and prioritization sensitivity

The 12-series resource contains 213 GEO sample records and 19 expression/count matrices. Matrix-level assignments reuse samples across processed-expression and count representations and are not additional biological replicates. Fifteen downloaded source files underlie these matrices. The series and matrix inventory is given in Table S8; complete sample mappings, public source files with verified SHA-256 hashes, and parsed matrices are provided in Data S5. The initial 89-protein inventory maps to 87 entries in the archived expression resource. The historical expression tables contain 93 candidate identifiers; Data S5 supplies an explicit 89-to-87 scope map and a retained-inventory evidence table.

Nine series are retained for the WT-induction display after excluding GSE253759 and GSE303416; GSE322740 has no comparable time-induction contrast. Induction denotes the series-specific condition and is not uniformly nematode exposure: GSE212735 uses nitrogen deficiency. The two unsupported/unmapped inventory entries remain NA rather than zero in the Figure 2 source table. Table S9 summarizes support changes and the 16-candidate exclusion analysis. Table S10 reports all 13 ranked candidates, alternative ranks and the signed regulator effects from which the scores were reconstructed.

S6.1. Source support criteria and statistical layers

For the archived screening-effect table used to generate candidate_evidence_by_gse.tsv, processed TPM/FPKM values were transformed as log2(value + 0.1). WT time and mutant-versus-WT effects were differences between group means on that transformed scale, with two-sided Welch t tests. Benjamini–Hochberg adjustment was performed within matrix × contrast-type × time families. A supported screening contrast required both adjusted $q < 0.05$ and $|\log_2 \text{fold change}| \geq 1$. Figure 2a dots read the resulting wt_induction_supported flag and therefore inherit both criteria; the abbreviated q-only key in the archived figure omits the effect-size criterion. A series contributes one supporting series when at least one of its WT contrasts meets both criteria. These screening effects, rather than the separately fitted series-model results, underpin the reported support counts and score reconstruction.

The archive also contains a separate series-model analysis: limma models for processed log2-expression matrices and DESeq2 v1.46.0 for raw-count matrices. Their model status, results and processed/count concordance tables are supplied separately in Data S5. They are not substituted for the screening-effect table. Per-series WT effects are summarized using the median of the available screening contrasts; this can encompass more than one time contrast and/or processed matrix. TPM and FPKM abundance values are not pooled across units.

Table S8. Public transcriptome resource and matrix inventory.

(A) GEO series. Sample counts refer to archived GEO sample records and sum to 213; the nine retained WT-display series are marked Yes. No unchanged supplementary table or figure has been renumbered.

GEO series	GEO samples	Matrices	Units	WT display	Condition scope
GSE210886	24	2	TPM	Yes	Series-specific nematode induction
GSE212735	12	1	TPM	Yes	Nitrogen deficiency
GSE213542	12	1	FPKM	Yes	Series-specific nematode induction
GSE215079	24	1	TPM	Yes	Series-specific nematode induction
GSE245014	36	2	TPM	Yes	Series-specific nematode induction
GSE253759	24	1	TPM	No: duplicate-like	Series-specific nematode induction
GSE301423	18	2	TPM/count	Yes	Series-specific nematode induction
GSE301452	12	2	FPKM/count	Yes	Series-specific nematode induction
GSE303416	12	2	FPKM/count	No: provisional mapping	Series-specific nematode induction
GSE306156	12	2	TPM/count	Yes	Series-specific nematode induction
GSE322548	18	2	TPM/count	Yes	Series-specific nematode induction
GSE322740	9	1	FPKM	No: day-5 single state	Day-5 aerial hyphae

(B) Matrix identifiers and mapping. Raw counts and their corresponding normalized expression matrices may derive from the same source file and samples. The 19-matrix total is not a count of independent experiments. Full column-to-sample mappings and source hashes are in Data S5.

Matrix identifier	Unit	Samples	Time points	Mapping status	Model layer
GSE210886_Acy	TPM	12	0h;10h	title_based	Processed screening / limma
GSE210886_PKA	TPM	12	0h;10h	title_based	Processed screening / limma
GSE212735	TPM	12	0h;10h	title_based	Processed screening / limma
GSE213542	FPKM	12	0h;24h	title_based	Processed screening / limma
GSE215079	TPM	24	0h;6h;10h;24h	title_based	Processed screening / limma

Matrix identifier	Unit	Samples	Time points	Mapping status	Model layer
GSE245014_Rab7	TPM	18	0h;10h;36h	title_based	Processed screening / limma
GSE245014_Vps35	TPM	18	0h;10h;36h	title_based	Processed screening / limma
GSE253759	TPM	24	0h;6h;10h;24h	title_based	Processed screening / limma
GSE301423_TPM	TPM	18	0h;24h	title_based	Processed screening / limma
GSE301423_counts	count	18	0h;24h	title_based	DESeq2 (separate)
GSE301452_FPKM	FPKM	12	0h;24h	title_based	Processed screening / limma
GSE301452_counts	count	12	0h;24h	title_based	DESeq2 (separate)
GSE303416_FPKM	FPKM	12	0h;10h	order_based	Processed screening / limma
GSE303416_counts	count	12	0h;10h	order_based	DESeq2 (separate)
GSE306156_TPM	TPM	12	0h;18h	title_based	Processed screening / limma
GSE306156_counts	count	12	0h;18h	title_based	DESeq2 (separate)
GSE322548_TPM	TPM	18	0h;10h;24h	title_based	Processed screening / limma
GSE322548_counts	count	18	0h;10h;24h	title_based	DESeq2 (separate)
GSE322740_FPKM	FPKM	9	Day5_or_unknown	title_based	Processed screening / limma

Table S9. WT-response support and exclusion sensitivity.

(A) Global support among 87 mapped candidates. Support requires $q < 0.05$ and $|\log2FC| \geq 1$ in at least one underlying WT-induction contrast. Distinct series, rather than contrasts, are counted. The original 79/54 values belong to the 11-series atlas; the retained nine-series analysis yields 78/47.

Scenario	WT series	Mapped	≥1 supporting series	≥3 supporting series
Full WT atlas	11	87	79	54
Exclude GSE303416	10	87	78	51
Exclude GSE253759	10	87	79	50
Exclude both	9	87	78	47

(B) Number of supporting WT series for each of the 16 filter-positive candidates. GIN2, GIN6 and GIN9 are retained here to document the filter before their exclusion from the 13-candidate ranking.

Candidate	Alias / prior name	Full 11	−GSE303416	−GSE253
AOL_s00007g309.1	AoGrn1	3	3	2
AOL_s00054g605.1	GIN2	5	5	4
AOL_s00054g150.1	AoGrn3	7	7	6
AOL_s00176g42.1	AoGrn2	5	5	4
AOL_s00076g450.1	—	4	4	3
AOL_s00004g22.1	—	6	6	5
AOL_s00097g309.3	—	2	1	2
AOL_s00173g137.1	—	5	5	4
AOL_s00054g270.1	—	4	4	3
AOL_s00210g385.1	GIN6	4	4	3
AOL_s00075g112.1	—	4	4	3
AOL_s00007g90.1	—	2	1	2
AOL_s00210g301.1	GIN9	4	3	4
AOL_s00215g906.3	—	6	5	5
AOL_s00043g686.1	—	4	4	4
AOL_s00080g416.1	—	1	1	1

(C) Leave-one-series-out audit of the 11 original WT-series contrasts. Removal of GSE210886 leaves AOL_s00080g416.1 without WT support; the other 15 candidates retain support. The archived raw audit additionally includes removal of GSE322740, a no-WT-contrast catalogue record, which is a no-op check; all 192 candidate-by-catalogue-exclusion records are retained in Data S5.

Excluded WT series	Candidates retaining support /16	Candidate losing WT support
GSE210886	15	AOL_s00080g416.1
GSE212735	16	None
GSE213542	16	None
GSE215079	16	None
GSE245014	16	None
GSE253759	16	None
GSE301423	16	None
GSE301452	16	None
GSE303416	16	None
GSE306156	16	None
GSE322548	16	None

Table S10. Candidate ranking and regulator-effect sensitivity.

(A) Original and alternative module summaries for all 13 uncharacterized candidates. For regulator r, m(r) is the median mutant-versus-WT screening log2FC across available times. Original score = max(|m(Hog1)|, |m(Opy2)|) + max(|m(Acy)|, |m(PkaC1)|). Alternative score replaces each module maximum by the median of its absolute regulator effects. Values are displayed to three decimals; full precision is retained in Data S5. Ranks use minimum rank for ties.

Candidate	Alias	Original score	Original rank	Alternative score
AOL_s00007g309.1	AoGrn1	6.260	1	4.445
AOL_s00054g150.1	AoGrn3	4.731	2	3.080
AOL_s00176g42.1	AoGrn2	4.702	3	3.276
AOL_s00076g450.1	—	4.692	4	2.534
AOL_s00004g22.1	—	3.969	5	2.452
AOL_s00097g309.3	—	3.549	6	2.180
AOL_s00173g137.1	—	3.218	7	2.412
AOL_s00054g270.1	—	3.163	8	2.313
AOL_s00075g112.1	—	2.967	9	1.910
AOL_s00007g90.1	—	2.950	10	1.753
AOL_s00215g906.3	—	2.639	11	1.393
AOL_s00043g686.1	—	2.181	12	1.484
AOL_s00080g416.1	—	1.785	13	1.429

(B) Rank under each single-regulator combination. Combination scores sum the absolute regulator medians for one MAPK regulator and one cAMP–PKA regulator. The three advanced candidates remain exploratory choices; ranks can change across component combinations.

Candidate	Alias	Hog1 + Acy	Hog1 + PkaC1	Opy2 + Acy
AOL_s00007g309.1	AoGrn1	1	1	3
AOL_s00054g150.1	AoGrn3	2	4	4
AOL_s00176g42.1	AoGrn2	3	6	1
AOL_s00076g450.1	—	9	2	13
AOL_s00004g22.1	—	5	3	12
AOL_s00097g309.3	—	4	11	2
AOL_s00173g137.1	—	10	5	10
AOL_s00054g270.1	—	6	7	7
AOL_s00075g112.1	—	7	10	5
AOL_s00007g90.1	—	8	8	9
AOL_s00215g906.3	—	11	12	8
AOL_s00043g686.1	—	12	13	6
AOL_s00080g416.1	—	13	9	11

(C) Signed regulator medians underlying the 13 ranked candidates. The full 64-row regulator table for all 16 filter-positive candidates includes contrast and supported-contrast counts in Data S5. A negative value denotes lower expression in the mutant than WT in the archived genotype-effect summary; it does not establish pathway placement of the receptor.

Candidate	Alias	AoHog1	AoOpy2	AoAcy
AOL_s00007g309.1	AoGrn1	+4.545	+1.255	-1.715
AOL_s00054g150.1	AoGrn3	+3.072	-0.952	-1.659
AOL_s00176g42.1	AoGrn2	-2.072	+1.198	+2.630
AOL_s00076g450.1	—	+2.769	+0.251	-0.126
AOL_s00004g22.1	—	+2.807	+0.460	-0.475
AOL_s00097g309.3	—	+0.771	+0.325	+2.777
AOL_s00173g137.1	—	+1.996	+0.760	+0.846
AOL_s00054g270.1	—	+1.237	+0.080	+1.926
AOL_s00075g112.1	—	+1.058	+0.547	+1.909
AOL_s00007g90.1	—	+1.426	+0.217	+1.524
AOL_s00215g906.3	—	+0.936	-0.100	+1.703
AOL_s00043g686.1	—	+0.169	-0.313	+1.869
AOL_s00080g416.1	—	-0.443	+0.742	-0.629

Data S7 Trap Induction Source Data

S7A Original Trap Counts

Each entry is the integer trap count in a combined sampled area of 4.5 cm² per plate. Ten plates were assayed per group. Plate numbers identify observations within groups; no pairing across groups is implied.

Counts in sampled area groups 1 to 6

Plate	WT	ΔGrn1-1	ΔGrn1-2	ΔGrn1-3	ΔGrn1c	ΔGrn2-1
1	485	88	182	233	355	7
2	342	161	177	149	413	11
3	596	61	59	192	287	71
4	548	146	117	161	273	47
5	766	218	191	152	336	39
6	594	141	180	77	386	7
7	426	232	273	74	238	31
8	312	115	154	31	205	117
9	641	63	155	289	500	20
10	502	52	94	183	260	229

Counts in sampled area groups 7 to 12

Plate	ΔGrn2-2	ΔGrn2-3	ΔGrn2c	ΔGrn3-1	ΔGrn3-2	ΔGrn3-3
1	172	26	180	427	395	561
2	138	20	156	401	491	397
3	37	39	193	431	507	491
4	162	31	224	554	581	434
5	21	113	205	437	512	677
6	23	120	293	499	591	654
7	31	79	213	484	674	631
8	9	73	327	510	584	591
9	71	77	272	301	674	497
10	112	21	169	639	491	581

Source: TrapsNum_original.xlsx, Sheet1, B2:M11. All 120 source counts are reproduced in worksheet order.

S7B Trap Density for Every Plate

Density = count / 4.5 cm², reported here in traps/cm² to two decimal places. Tests and summaries were calculated from full precision values.

Density in traps per cm² groups 1 to 6

Plate	WT	ΔGrn1-1	ΔGrn1-2	ΔGrn1-3	ΔGrn1c	ΔGrn2-1
1	107.78	19.56	40.44	51.78	78.89	1.56
2	76.00	35.78	39.33	33.11	91.78	2.44
3	132.44	13.56	13.11	42.67	63.78	15.78
4	121.78	32.44	26.00	35.78	60.67	10.44
5	170.22	48.44	42.44	33.78	74.67	8.67
6	132.00	31.33	40.00	17.11	85.78	1.56
7	94.67	51.56	60.67	16.44	52.89	6.89
8	69.33	25.56	34.22	6.89	45.56	26.00
9	142.44	14.00	34.44	64.22	111.11	4.44
10	111.56	11.56	20.89	40.67	57.78	50.89

Density in traps per cm² groups 7 to 12

Plate	ΔGrn2-2	ΔGrn2-3	ΔGrn2c	ΔGrn3-1	ΔGrn3-2	ΔGrn3-3
1	38.22	5.78	40.00	94.89	87.78	124.67
2	30.67	4.44	34.67	89.11	109.11	88.22
3	8.22	8.67	42.89	95.78	112.67	109.11
4	36.00	6.89	49.78	123.11	129.11	96.44
5	4.67	25.11	45.56	97.11	113.78	150.44
6	5.11	26.67	65.11	110.89	131.33	145.33
7	6.89	17.56	47.33	107.56	149.78	140.22
8	2.00	16.22	72.67	113.33	129.78	131.33
9	15.78	17.11	60.44	66.89	149.78	110.44
10	24.89	4.67	37.56	142.00	109.11	129.11

Source: plate_counts_and_densities.csv. Scaling every observation by the same positive constant preserves ranks and therefore leaves the exact rank test P values unchanged.

S7C Trap Group Summaries

Group	n	Count mean ± SD	Density mean ± SD	Shapiro–Wilk P
WT	10	521.20 ± 138.45	115.82 ± 30.77	0.945685
ΔGrn1-1	10	127.70 ± 63.91	28.38 ± 14.20	0.373706
ΔGrn1-2	10	158.20 ± 59.01	35.16 ± 13.11	0.688115
ΔGrn1-3	10	154.10 ± 77.67	34.24 ± 17.26	0.893692
ΔGrn1c	10	325.30 ± 90.34	72.29 ± 20.08	0.811273
ΔGrn2-1	10	57.90 ± 69.09	12.87 ± 15.35	0.004066
ΔGrn2-2	10	77.60 ± 62.91	17.24 ± 13.98	0.095312
ΔGrn2-3	10	59.90 ± 37.69	13.31 ± 8.38	0.126596
ΔGrn2c	10	223.20 ± 56.47	49.60 ± 12.55	0.382608
ΔGrn3-1	10	468.30 ± 91.90	104.07 ± 20.42	0.93554
ΔGrn3-2	10	550.00 ± 87.45	122.22 ± 19.43	0.517574
ΔGrn3-3	10	551.40 ± 93.93	122.53 ± 20.87	0.727036

Count units: traps per 4.5 cm^2 sampled area. Density units: traps/cm^2. SD is the sample standard deviation. Shapiro–Wilk values are reproduced from group_summary_and_normality.csv.

Experimental units and interpretation

All 120 plates came from one trap induction batch using approximately 200 live L4 stage C. elegans per plate. Three separately labelled deletion isolates per gene originated from one transformation batch per gene. One complemented isolate per gene was tested for Grn1 and Grn2. Ten plates per group represent plate replication within this assay.

The archived primary analysis uses two sided unpaired Welch t tests with Holm adjustment across 17 comparisons. Exact rank based permutation tests use the same 17 comparisons as a distributional sensitivity analysis. One deletion group, ΔGrn2-1, has Shapiro–Wilk $P = 0.004066$. The permutation analysis assumes exchangeability of plate labels under the null; inference remains conditional on this batch.

Fourteen of the 17 rank comparisons have Holm adjusted $P < 0.05$. All three Grn3 deletion isolates have adjusted $P = 1$ versus WT. Both complemented isolates differ from their corresponding deletion isolates and also remain different from WT.

S7D Exact Rank Tests for Trap Induction

Each comparison contains 10 plates per group. All C(20,10) = 184,756 allocations were enumerated with average ranks for ties. U is defined for the first named group. Two sided P is twice the smaller inclusive tail probability, capped at 1. Holm adjustment is joint across all 17 comparisons.

Group 1	Group 2	U_1	Exact rank P	Rank P Holm 17	Welch P Holm 17
ΔGrn1-1	WT	0	1.08251e-05	0.000184026	2.7801e-05
ΔGrn1-2	WT	0	1.08251e-05	0.000184026	6.18493e-05
ΔGrn1-3	WT	0	1.08251e-05	0.000184026	4.31463e-05
ΔGrn1c	WT	11	0.002089	0.008357	0.00739
ΔGrn2-1	WT	0	1.08251e-05	0.000184026	5.00159e-06
ΔGrn2-2	WT	0	1.08251e-05	0.000184026	9.52175e-06
ΔGrn2-3	WT	0	1.08251e-05	0.000184026	1.58074e-05
ΔGrn2c	WT	1	2.16502e-05	0.000216502	0.000312491
ΔGrn3-1	WT	37	0.352681	1	0.988205
ΔGrn3-2	WT	56	0.670582	1	1
ΔGrn3-3	WT	57	0.630529	1	1
ΔGrn1c	ΔGrn1-1	98	4.33004e-05	0.000389703	0.000312491
ΔGrn1c	ΔGrn1-2	96.5	8.66007e-05	0.000606205	0.001058
ΔGrn1c	ΔGrn1-3	94	0.000324753	0.001949	0.001323
ΔGrn2c	ΔGrn2-1	93	0.000487129	0.002436	0.000176309
ΔGrn2c	ΔGrn2-2	97	7.57756e-05	0.000606205	0.000312491
ΔGrn2c	ΔGrn2-3	100	1.08251e-05	0.000184026	1.67931e-05

The family comprises 11 non WT versus WT comparisons and 6 comparisons of the Grn1 or Grn2 complemented isolate against its three corresponding deletion isolates. Exact rank P values and Holm values were independently recalculated from the original workbook and agree with statistics_17_comparisons.csv.

Welch Holm values are included to distinguish the archived primary analysis from the rank sensitivity analysis. They are reproduced from statistics_17_comparisons.csv. No claim of equivalence follows from a nonsignificant comparison.

S3A Growth Source Data for WT and Grn2

Colony diameters in cm for days 1–6. The same five plates per strain were followed over time. AUC is the trapezoidal integral over days 1–5, in cm·day. Day 6 remains in the raw table and is excluded from the primary AUC because growth was plate limited.

Strain	Plate	D1	D2	D3	D4	D5	D6	AUC D1–5
WT	1	0	2	3	4	5	6	11.50
WT	2	0	1	2	3.8	5	6	9.30
WT	3	0	1.2	2.4	3.2	4.9	5.8	9.25
WT	4	0	0.8	2.1	3.5	4.8	6	8.80
WT	5	0	1.1	2.3	2.9	5	5.9	8.80
ΔGrn2c	1	0	0.9	2	2.3	5.1	6	7.75
ΔGrn2c	2	0	0.2	1.8	3.4	4.9	5.4	7.85
ΔGrn2c	3	0	0.1	2.1	3.2	4.5	5.3	7.65
ΔGrn2c	4	0	0.3	1.5	3	4.8	6	7.20
ΔGrn2c	5	0	0.2	1.9	2.8	5.1	5.9	7.45
ΔGrn2-1	1	0	0.2	2.5	3.4	4.6	5.9	8.40
ΔGrn2-1	2	0	0.7	1.7	2.9	5.3	5.7	7.95
ΔGrn2-1	3	0	0.4	2.5	2.9	4.9	5.7	8.25
ΔGrn2-1	4	0	0.9	2.5	2.7	3.8	5.9	8.00
ΔGrn2-1	5	0	0.1	2.4	3.1	3.6	5.9	7.40
ΔGrn2-2	1	0	0.3	1.5	2.6	4.8	6	6.80
ΔGrn2-2	2	0	0.1	1.6	3	3.6	5.8	6.50
ΔGrn2-2	3	0	0.9	2.4	3.1	4.7	5.7	8.75
ΔGrn2-2	4	0	0.9	1.5	2.9	5.5	5.9	8.05
ΔGrn2-2	5	0	0.3	1.3	3.1	4.7	5.7	7.05
ΔGrn2-3	1	0	0.7	1.9	2.6	4.3	6	7.35
ΔGrn2-3	2	0	0.8	2.5	2.6	4.9	5.9	8.35
ΔGrn2-3	3	0	0.6	2.1	3.3	4.5	5.8	8.25
ΔGrn2-3	4	0	0.5	1.9	2.9	4.6	5.9	7.60
ΔGrn2-3	5	0	0.2	1.2	2.7	5.5	5.9	6.85

Source: GrowthData_original.xlsx, Sheet1, B2:Z7; plate_endpoints.csv. Each five column block is one strain, with plates ordered 1–5 from left to right. D denotes day.

S3B Growth Source Data for Grn1

Colony diameters are in cm; AUC D1–5 is in cm·day. Table columns and calculation rules are identical to S3A.

Strain	Plate	D1	D2	D3	D4	D5	D6	AUC D1–5
ΔGrn1c	1	0	0.1	2	3.1	4.3	5.6	7.35
ΔGrn1c	2	0	0.9	2.6	3.3	5.5	6	9.55
ΔGrn1c	3	0	0.9	1.2	2.8	5.4	5.8	7.60
ΔGrn1c	4	0	0.6	1.8	3.2	3.9	5.9	7.55
ΔGrn1c	5	0	0.3	2.4	2.7	3.6	5.7	7.20
ΔGrn1-1	1	0	0.3	2.2	2.6	3.7	6	6.95
ΔGrn1-1	2	0	0.7	2.1	3.1	3.5	5.8	7.65
ΔGrn1-1	3	0	0.9	2.1	3.1	3.7	5.6	7.95
ΔGrn1-1	4	0	0.9	1.6	3.2	4.9	6	8.15
ΔGrn1-1	5	0	0.8	1.5	3.3	3.6	5.6	7.40
ΔGrn1-2	1	0	0.6	2.3	3.5	4.2	5.7	8.50
ΔGrn1-2	2	0	0.1	2.3	3.1	5.3	6	8.15
ΔGrn1-2	3	0	0.2	2.4	2.9	3.5	6	7.25
ΔGrn1-2	4	0	0.9	2.5	3.3	5.2	5.8	9.30
ΔGrn1-2	5	0	0.6	1.8	2.8	4.1	5.9	7.25
ΔGrn1-3	1	0	0	1.8	3	3.7	5.8	6.65
ΔGrn1-3	2	0	0.1	2.3	2.7	5	5.7	7.60
ΔGrn1-3	3	0	0.7	2	2.6	4.8	5.7	7.70
ΔGrn1-3	4	0	0	2.1	3.5	5.3	5.9	8.25
ΔGrn1-3	5	0	0.2	2.3	3.4	5	5.9	8.40

Source: GrowthData_original.xlsx, Sheet1, AA2:AT7; plate_endpoints.csv. This table completes all 270 daily measurements and 45 plate AUCs.

AUC calculation and ties

For observations one day apart, the primary AUC equals half the day 1 diameter plus the full day 2, day 3 and day 4 diameters, plus half the day 5 diameter. For WT plate 1, the diameters 0, 2, 3, 4 and 5 cm give an AUC of 11.50 cm·day.

AUCs are recorded to 0.01 cm·day. Mathematical ties at this precision were retained. For the exact tests, average ranks were assigned to tied AUC values and all 252 allocations of the ten labels were enumerated.

S3C Growth AUC Summaries and Exact Tests

Strain	n	Mean AUC	SD	Median AUC
WT	5	9.530	1.127	9.250
ΔGrn2c	5	7.580	0.259	7.650
ΔGrn2-1	5	8.000	0.382	8.000
ΔGrn2-2	5	7.430	0.940	7.050
ΔGrn2-3	5	7.680	0.628	7.600
ΔGrn1c	5	7.850	0.964	7.550
ΔGrn1-1	5	7.620	0.471	7.650
ΔGrn1-2	5	8.090	0.873	8.150
ΔGrn1-3	5	7.720	0.690	7.700

AUC units: cm·day. WT mean AUC = 9.530 cm·day. Sample SDs and medians were calculated from all five plate values.

Eight exact comparisons against WT

Strain versus WT	U_1	Mean difference	Exact P	P Holm 8
ΔGrn2c	0	-1.950	0.007937	0.063492
ΔGrn2-1	0	-1.530	0.007937	0.063492
ΔGrn2-2	0	-2.100	0.007937	0.063492
ΔGrn2-3	0	-1.850	0.007937	0.063492
ΔGrn1c	4	-1.680	0.079365	0.142857
ΔGrn1-1	0	-1.910	0.007937	0.063492
ΔGrn1-2	3.5	-1.440	0.071429	0.142857
ΔGrn1-3	0	-1.810	0.007937	0.063492

Each test compares five non WT plates against the same five WT plates. Mean difference is non WT minus WT in cm·day. U is the rank statistic for the non WT strain. The P values test rank distributions; the mean difference is descriptive. All eight Holm adjusted P values exceed 0.05.

Source: primary_AUC_comparisons.csv, columns U, p_mw_exact and p_mw_holm. These values were independently recalculated from the original diameters. The archived p_exact_permutation column instead tests a mean difference; it is a separate sensitivity analysis.

Data S6 Structural Scores and Sensitivity Analyses

International Journal of Molecular Sciences | Manuscript ijms-4611842

This dataset documents the initial structural screen of 89 candidate proteins against three Gα subunits, yielding 267 combinations and 801 scored models at seed 0. It also reports 18 additional scores at seed 101 for AoGrn1 and AoGrn2, complete receptor ranking checks, and orthogroup assignments for the 89 screened proteins.

Two entries, AOL_s00076g157.1 and AOL_s00188g124.1, were retained in the initial structural screen but excluded from the subsequent 87-candidate transcriptome prioritization. The 36-row two-seed focal table comprises 18 original seed-0 scores and 18 additional seed-101 scores, giving 819 distinct scoring records overall.

S6A Scope and archived modeling configuration

Parameter	Archived value
Model type	alphafold2_multimer_v3
Software	LocalColabFold 1.5.5
Commit	e8ebd9a612b9da5fe12bbbc09513d5c52a9af633
MSA mode	mmseqs2_uniref_env
Pair mode and strategy	unpaired_paired; greedy
Model weights	1, 2 and 3
Maximum recycles; early-stop tolerance	3; 0.5
Random seed in archived configuration	0
Additional focal random seed	101
Templates; relaxation; dropout	False; 0 relaxed models; False
Ensemble; maximum sequences; extra sequences	1; 508; 2048

Parameter	Archived value
Archived num_queries value	12; configuration metadata, not the complete screen total

Source: config.json. The original screen consists of three model weights at one seed, not three independent random seeds or biological replicates. The num_queries value is reproduced as archived and does not establish a single configuration file covering all 267 jobs.

Partners were ranked within each receptor by maximum ipTM across model weights and compared with mean ipTM and weight-specific rankings. Max PAE in the score tables is the archived global maximum PAE in Å; it is not an interface-specific PAE. Score preferences and margins are descriptive computational measures and do not establish biological coupling specificity.

S6B Focal receptors and partner rankings

AoGrn1 = AOL_s00007g309.1; AoGrn2 = AOL_s00176g42.1; AoGrn3 = AOL_s00054g150.1. The fourth receptor below retains its archived locus identifier. Each row summarizes three seed-0 model weights.

Receptor	Partner	Min ipTM	Median	Mean	Max	SD
AoGrn1	Gpa1	0.670000	0.690000	0.686667	0.700000	0.015275
AoGrn1	Gpa2	0.180000	0.290000	0.323333	0.500000	0.162583
AoGrn1	Gpa3	0.570000	0.600000	0.593333	0.610000	0.020817
AoGrn3	Gpa1	0.610000	0.660000	0.646667	0.670000	0.032146
AoGrn3	Gpa2	0.440000	0.530000	0.513333	0.570000	0.066583
AoGrn3	Gpa3	0.300000	0.560000	0.473333	0.560000	0.150111
AOL_s00076g450.1	Gpa1	0.670000	0.690000	0.686667	0.700000	0.015275
AOL_s00076g450.1	Gpa2	0.260000	0.310000	0.303333	0.340000	0.040415
AOL_s00076g450.1	Gpa3	0.600000	0.620000	0.623333	0.650000	0.025166
AoGrn2	Gpa1	0.290000	0.350000	0.336667	0.370000	0.041633
AoGrn2	Gpa2	0.200000	0.230000	0.223333	0.240000	0.020817
AoGrn2	Gpa3	0.210000	0.220000	0.220000	0.230000	0.010000

Source: af2_focal_pair_sensitivity.tsv. Model scores for all four focal receptors are included in the complete score tables below.

Rank stability between seeds 0 and 101

Receptor	Seed	Max winner	Mean winner	Max margin	Mean margin	W1 / W2 / W3
AoGrn1	0	Gpa1	Gpa1	0.090000	0.093333	Gpa1 / Gpa1 / Gpa1
AoGrn1	101	Gpa1	Gpa1	0.100000	0.100000	Gpa1 / Gpa1 / Gpa1
AoGrn2	0	Gpa1	Gpa1	0.130000	0.113333	Gpa1 / Gpa1 / Gpa1
AoGrn2	101	Gpa1	Gpa1	0.130000	0.106667	Gpa1 / Gpa1 / Gpa1

Source: af2_focal_seed_sensitivity.tsv. Both AoGrn1 and AoGrn2 retain Gpa1 as the top-scoring partner under the maximum, mean and all three model-weight rules at both seeds. This stability concerns computational score ranking. For AoGrn2–Gpa1, ipTM spans 0.29–0.37 across the evaluated weights and seeds.

S6C Complete focal scores at two random seeds

All 36 focal scoring records are reproduced. W denotes model weight. Rank is the archived rank within a receptor–Gα job. Ranking-confidence entries were blank in the source and are not imputed. Original score files are identified by the deterministic naming rule in S6H.

Receptor	Partner	Seed	W	Rank	ipTM	pTM	Max PAE Å
AoGrn1	Gpa1	0	1	1	0.7	0.73	31.546875
AoGrn1	Gpa1	0	2	2	0.69	0.72	31.65625
AoGrn1	Gpa1	0	3	3	0.67	0.7	31.6875
AoGrn1	Gpa2	0	1	2	0.29	0.49	31.640625
AoGrn1	Gpa2	0	2	1	0.5	0.59	31.6875
AoGrn1	Gpa2	0	3	3	0.18	0.47	31.71875
AoGrn1	Gpa3	0	1	2	0.6	0.65	31.609375
AoGrn1	Gpa3	0	2	1	0.61	0.65	31.640625
AoGrn1	Gpa3	0	3	3	0.57	0.63	31.65625
AoGrn1	Gpa1	101	1	1	0.7	0.73	31.546875
AoGrn1	Gpa1	101	2	2	0.69	0.71	31.671875
AoGrn1	Gpa1	101	3	3	0.67	0.7	31.65625
AoGrn1	Gpa2	101	1	2	0.51	0.58	31.625
AoGrn1	Gpa2	101	2	1	0.55	0.61	31.6875
AoGrn1	Gpa2	101	3	3	0.45	0.57	31.703125
AoGrn1	Gpa3	101	1	2	0.59	0.65	31.609375
AoGrn1	Gpa3	101	2	1	0.6	0.64	31.671875
AoGrn1	Gpa3	101	3	3	0.57	0.63	31.671875
AoGrn2	Gpa1	0	1	2	0.35	0.51	31.65625
AoGrn2	Gpa1	0	2	1	0.37	0.5	31.6875
AoGrn2	Gpa1	0	3	3	0.29	0.46	31.6875
AoGrn2	Gpa2	0	1	3	0.2	0.44	31.671875
AoGrn2	Gpa2	0	2	2	0.23	0.42	31.703125
AoGrn2	Gpa2	0	3	1	0.24	0.45	31.71875
AoGrn2	Gpa3	0	1	3	0.21	0.47	31.671875
AoGrn2	Gpa3	0	2	1	0.23	0.47	31.703125
AoGrn2	Gpa3	0	3	2	0.22	0.44	31.703125
AoGrn2	Gpa1	101	1	1	0.36	0.52	31.6875
AoGrn2	Gpa1	101	2	2	0.34	0.48	31.6875
AoGrn2	Gpa1	101	3	3	0.29	0.46	31.703125
AoGrn2	Gpa2	101	1	3	0.19	0.45	31.6875
AoGrn2	Gpa2	101	2	2	0.22	0.43	31.703125
AoGrn2	Gpa2	101	3	1	0.21	0.44	31.71875
AoGrn2	Gpa3	101	1	2	0.21	0.47	31.6875
AoGrn2	Gpa3	101	2	1	0.23	0.47	31.6875
AoGrn2	Gpa3	101	3	3	0.23	0.42	31.703125

Source: af2_focal_two_seed_scores.tsv. Seed 0 contributes 18 original records; seed 101 contributes 18 additional records.

S6D Focal score ranges and available structures

Receptor	Partner	Seed	Min ipTM	Mean ipTM	Max ipTM
AoGrn1	Gpa1	0	0.67	0.686667	0.7
AoGrn1	Gpa1	101	0.67	0.686667	0.7
AoGrn1	Gpa2	0	0.18	0.323333	0.5
AoGrn1	Gpa2	101	0.45	0.503333	0.55
AoGrn1	Gpa3	0	0.57	0.593333	0.61
AoGrn1	Gpa3	101	0.57	0.586667	0.6
AoGrn2	Gpa1	0	0.29	0.336667	0.37
AoGrn2	Gpa1	101	0.29	0.330000	0.36
AoGrn2	Gpa2	0	0.2	0.223333	0.24
AoGrn2	Gpa2	101	0.19	0.206667	0.22
AoGrn2	Gpa3	0	0.21	0.220000	0.23
AoGrn2	Gpa3	101	0.21	0.223333	0.23

Source: af2_focal_seed_pair_ranges.tsv. Each range uses three model weights at the indicated seed.

Available representative PDB coordinates

File ID	Receptor	Partner	Seed	Weight	Rank	Bytes
P01	AoGrn1	Gpa1	0	1	1	471339
P02	AoGrn1	Gpa2	0	2	1	473850
P03	AoGrn1	Gpa3	0	2	1	474336
P04	AoGrn2	Gpa1	0	2	1	524880
P05	AoGrn2	Gpa2	0	3	1	527391
P06	AoGrn2	Gpa3	0	2	1	527877
P07	AoGrn1	Gpa1	101	1	1	471339
P08	AoGrn1	Gpa2	101	2	1	473850
P09	AoGrn1	Gpa3	101	2	1	474336
P10	AoGrn2	Gpa1	101	1	1	524880
P11	AoGrn2	Gpa2	101	3	1	527391
P12	AoGrn2	Gpa3	101	2	1	527877

The 12 coordinate files are located under inputs/focal_structures/seed0/ and seed101/. Their basename follows <job>_unrelaxed_rank_<rank3>_alphafold2_multimer_v3_model_<weight>_seed_<seed3>.pdb, where <job> is the receptor identifier with its final dot replaced by an underscore, followed by __AOGpa1, __AOGpa2 or __AOGpa3. Zero-padded rank and seed fields have three digits. Full coordinate models for every one of the 267 combinations are not present in this archive.

S6E Ranking robustness across all 89 receptors part 1

Winner is the highest ipTM partner. Ties follow the archived alphabetical partner ordering. Max and mean margins are top-minus-second score differences. Support counts model weights agreeing with the maximum-based winner; model weights are computational settings.

Receptor	Max winner	Mean winner	Max margin	Mean margin	Weight winners W1 / W2 / W3	Support /3
AOL_s00004g178.1	Gpa1	Gpa1	0.300000	0.193333	Gpa1 / Gpa1 / Gpa2	2
AOL_s00004g22.1	Gpa1	Gpa1	0.140000	0.226667	Gpa1 / Gpa1 / Gpa1	3
AOL_s00004g290.1	Gpa1	Gpa1	0.110000	0.103333	Gpa1 / Gpa1 / Gpa3	2
AOL_s00006g420.1	Gpa2	Gpa1	0.020000	0.010000	Gpa2 / Gpa2 / Gpa1	2
AOL_s00006g519.1	Gpa1	Gpa1	0.040000	0.120000	Gpa1 / Gpa1 / Gpa1	3
AOL_s00007g229.1	Gpa1	Gpa1	0.010000	0.013333	Gpa1 / Gpa1 / Gpa1	3
AOL_s00007g309.1	Gpa1	Gpa1	0.090000	0.093333	Gpa1 / Gpa1 / Gpa1	3
AOL_s00007g90.1	Gpa1	Gpa1	0.090000	0.140000	Gpa1 / Gpa1 / Gpa1	3
AOL_s00043g195.1	Gpa1	Gpa1	0.130000	0.226667	Gpa1 / Gpa1 / Gpa1	3
AOL_s00043g223.1	Gpa3	Gpa1	0.050000	0.203333	Gpa1 / Gpa1 / Gpa3	1
AOL_s00043g267.1	Gpa1	Gpa1	0.070000	0.090000	Gpa1 / Gpa1 / Gpa1	3
AOL_s00043g324.1	Gpa1	Gpa1	0.250000	0.286667	Gpa1 / Gpa1 / Gpa1	3
AOL_s00043g686.1	Gpa1	Gpa1	0.260000	0.153333	Gpa1 / Gpa1 / Gpa2	2
AOL_s00043g705.1	Gpa2	Gpa2	0.070000	0.036667	Gpa2 / Gpa2 / Gpa2	3
AOL_s00043g731.1	Gpa1	Gpa1	0.120000	0.173333	Gpa1 / Gpa1 / Gpa1	3
AOL_s00043g738.2	Gpa1	Gpa1	0.210000	0.206667	Gpa1 / Gpa1 / Gpa1	3
AOL_s00054g135.1	Gpa1	Gpa1	0.370000	0.300000	Gpa1 / Gpa1 / Gpa1	3
AOL_s00054g150.1	Gpa1	Gpa1	0.100000	0.133333	Gpa1 / Gpa1 / Gpa1	3
AOL_s00054g200.1	Gpa1	Gpa1	0.150000	0.226667	Gpa1 / Gpa1 / Gpa1	3
AOL_s00054g223.1	Gpa1	Gpa1	0.240000	0.283333	Gpa1 / Gpa1 / Gpa1	3
AOL_s00054g270.1	Gpa1	Gpa1	0.000000	0.013333	Gpa1 / Gpa1 / Gpa3	2
AOL_s00054g291.1	Gpa3	Gpa3	0.020000	0.010000	Gpa2 / Gpa3 / Gpa3	2
AOL_s00054g428.1	Gpa1	Gpa1	0.070000	0.056667	Gpa1 / Gpa1 / Gpa1	3
AOL_s00054g605.1	Gpa1	Gpa1	0.010000	0.056667	Gpa1 / Gpa1 / Gpa1	3
AOL_s00054g626.1	Gpa3	Gpa3	0.240000	0.073333	Gpa3 / Gpa3 / Gpa2	2
AOL_s00054g63.1	Gpa2	Gpa2	0.040000	0.000000	Gpa1 / Gpa3 / Gpa2	1
AOL_s00054g92.1	Gpa1	Gpa1	0.110000	0.266667	Gpa1 / Gpa1 / Gpa1	3
AOL_s00075g112.1	Gpa1	Gpa1	0.090000	0.263333	Gpa1 / Gpa1 / Gpa1	3
AOL_s00075g223.1	Gpa1	Gpa1	0.050000	0.026667	Gpa1 / Gpa1 / Gpa1	3
AOL_s00076g157.1	Gpa3	Gpa3	0.020000	0.006667	Gpa2 / Gpa2 / Gpa3	1
AOL_s00076g249.1	Gpa1	Gpa1	0.210000	0.226667	Gpa1 / Gpa1 / Gpa1	3
AOL_s00076g286.1	Gpa1	Gpa1	0.250000	0.223333	Gpa1 / Gpa1 / Gpa1	3
AOL_s00076g450.1	Gpa1	Gpa1	0.050000	0.063333	Gpa1 / Gpa1 / Gpa1	3
AOL_s00076g465.1	Gpa1	Gpa1	0.310000	0.273333	Gpa1 / Gpa1 / Gpa1	3
AOL_s00076g508.1	Gpa1	Gpa1	0.370000	0.403333	Gpa1 / Gpa1 / Gpa1	3
AOL_s00076g553.1	Gpa1	Gpa1	0.050000	0.076667	Gpa1 / Gpa1 / Gpa1	3
AOL_s00076g86.1	Gpa1	Gpa1	0.000000	0.266667	Gpa1 / Gpa1 / Gpa1	3
AOL_s00078g446.1	Gpa1	Gpa1	0.010000	0.003333	Gpa3 / Gpa1 / Gpa3	1
AOL_s00078g563.1	Gpa1	Gpa1	0.170000	0.156667	Gpa1 / Gpa1 / Gpa2	2
AOL_s00078g598.1	Gpa1	Gpa1	0.040000	0.063333	Gpa1 / Gpa1 / Gpa1	3
AOL_s00079g307.1	Gpa1	Gpa3	0.040000	0.016667	Gpa1 / Gpa1 / Gpa3	2
AOL_s00079g452.1	Gpa1	Gpa1	0.040000	0.110000	Gpa1 / Gpa1 / Gpa1	3
AOL_s00080g10.1	Gpa1	Gpa1	0.180000	0.206667	Gpa1 / Gpa1 / Gpa1	3
AOL_s00080g416.1	Gpa1	Gpa1	0.130000	0.136667	Gpa1 / Gpa1 / Gpa1	3
AOL_s00081g15.1	Gpa1	Gpa1	0.140000	0.160000	Gpa1 / Gpa1 / Gpa1	3

Source: af2_receptor_rank_robustness.tsv. Maximum and mean rankings agree for 84/89 receptors; all three model weights select the same partner for 69/89. The original maximum-based winner counts are Gpa1 79, Gpa2 5 and Gpa3 5.

S6E Ranking robustness across all 89 receptors part 2

Winner is the highest ipTM partner. Ties follow the archived alphabetical partner ordering. Max and mean margins are top-minus-second score differences. Support counts model weights agreeing with the maximum-based winner; model weights are computational settings.

Receptor	Max winner	Mean winner	Max margin	Mean margin	Weight winners W1 / W2 / W3	Support /3
AOL_s00081g30.1	Gpa1	Gpa3	0.000000	0.010000	Gpa1 / Gpa1 / Gpa3	2
AOL_s00083g156.1	Gpa1	Gpa1	0.060000	0.056667	Gpa1 / Gpa1 / Gpa1	3
AOL_s00097g260.1	Gpa1	Gpa1	0.300000	0.226667	Gpa1 / Gpa1 / Gpa1	3
AOL_s00097g290.1	Gpa1	Gpa1	0.330000	0.353333	Gpa1 / Gpa1 / Gpa1	3
AOL_s00097g309.3	Gpa1	Gpa1	0.170000	0.243333	Gpa1 / Gpa1 / Gpa1	3
AOL_s00097g558.3	Gpa1	Gpa1	0.120000	0.140000	Gpa1 / Gpa1 / Gpa1	3
AOL_s00097g69.1	Gpa2	Gpa2	0.300000	0.243333	Gpa2 / Gpa2 / Gpa2	3
AOL_s00109g63.1	Gpa1	Gpa1	0.160000	0.233333	Gpa1 / Gpa1 / Gpa1	3
AOL_s00109g8.1	Gpa1	Gpa1	0.040000	0.223333	Gpa1 / Gpa1 / Gpa1	3
AOL_s00110g16.1	Gpa1	Gpa1	0.210000	0.246667	Gpa1 / Gpa1 / Gpa1	3
AOL_s00110g307.1	Gpa1	Gpa1	0.030000	0.036667	Gpa1 / Gpa1 / Gpa1	3
AOL_s00110g337.1	Gpa1	Gpa1	0.100000	0.210000	Gpa1 / Gpa1 / Gpa1	3
AOL_s00140g54.6	Gpa1	Gpa1	0.070000	0.063333	Gpa1 / Gpa1 / Gpa1	3
AOL_s00169g110.1	Gpa1	Gpa1	0.170000	0.293333	Gpa1 / Gpa1 / Gpa1	3
AOL_s00169g261.1	Gpa1	Gpa1	0.100000	0.093333	Gpa1 / Gpa1 / Gpa1	3
AOL_s00170g20.1	Gpa1	Gpa1	0.200000	0.213333	Gpa1 / Gpa1 / Gpa1	3
AOL_s00170g54.1	Gpa1	Gpa1	0.180000	0.263333	Gpa1 / Gpa1 / Gpa1	3
AOL_s00170g99.1	Gpa1	Gpa1	0.170000	0.173333	Gpa1 / Gpa1 / Gpa1	3
AOL_s00173g137.1	Gpa1	Gpa1	0.280000	0.370000	Gpa1 / Gpa1 / Gpa1	3
AOL_s00173g258.2	Gpa2	Gpa2	0.080000	0.053333	Gpa2 / Gpa2 / Gpa2	3
AOL_s00173g37.1	Gpa1	Gpa1	0.110000	0.120000	Gpa1 / Gpa1 / Gpa1	3
AOL_s00176g24.1	Gpa1	Gpa1	0.270000	0.173333	Gpa1 / Gpa1 / Gpa2	2
AOL_s00176g42.1	Gpa1	Gpa1	0.130000	0.113333	Gpa1 / Gpa1 / Gpa1	3
AOL_s00188g124.1	Gpa1	Gpa1	0.020000	0.026667	Gpa1 / Gpa1 / Gpa1	3
AOL_s00188g142.1	Gpa1	Gpa1	0.080000	0.076667	Gpa1 / Gpa1 / Gpa1	3
AOL_s00188g284.1	Gpa1	Gpa1	0.110000	0.156667	Gpa1 / Gpa1 / Gpa1	3
AOL_s00188g320.1	Gpa1	Gpa1	0.220000	0.160000	Gpa1 / Gpa1 / Gpa2	2
AOL_s00188g342.1	Gpa1	Gpa1	0.170000	0.266667	Gpa1 / Gpa1 / Gpa1	3
AOL_s00188g346.1	Gpa1	Gpa1	0.250000	0.170000	Gpa1 / Gpa1 / Gpa3	2
AOL_s00193g107.1	Gpa1	Gpa1	0.040000	0.036667	Gpa1 / Gpa1 / Gpa1	3
AOL_s00210g135.2	Gpa1	Gpa1	0.110000	0.193333	Gpa1 / Gpa1 / Gpa1	3
AOL_s00210g301.1	Gpa1	Gpa1	0.090000	0.213333	Gpa1 / Gpa1 / Gpa1	3
AOL_s00210g381.1	Gpa1	Gpa1	0.350000	0.096667	Gpa1 / Gpa2 / Gpa1	2
AOL_s00210g385.1	Gpa1	Gpa1	0.210000	0.266667	Gpa1 / Gpa1 / Gpa1	3
AOL_s00215g252.1	Gpa1	Gpa1	0.170000	0.213333	Gpa1 / Gpa1 / Gpa1	3
AOL_s00215g301.1	Gpa1	Gpa1	0.290000	0.330000	Gpa1 / Gpa1 / Gpa1	3
AOL_s00215g417.1	Gpa1	Gpa1	0.220000	0.153333	Gpa1 / Gpa1 / Gpa2	2
AOL_s00215g444.1	Gpa1	Gpa1	0.080000	0.170000	Gpa1 / Gpa1 / Gpa1	3
AOL_s00215g562.1	Gpa3	Gpa1	0.010000	0.026667	Gpa3 / Gpa1 / Gpa3	2
AOL_s00215g603.1	Gpa1	Gpa1	0.110000	0.166667	Gpa1 / Gpa1 / Gpa1	3
AOL_s00215g796.1	Gpa1	Gpa1	0.130000	0.166667	Gpa1 / Gpa1 / Gpa1	3
AOL_s00215g906.3	Gpa1	Gpa1	0.100000	0.103333	Gpa1 / Gpa1 / Gpa1	3
AOL_s00215g915.1	Gpa1	Gpa1	0.190000	0.150000	Gpa1 / Gpa1 / Gpa1	3
AOL_s00215g924.1	Gpa1	Gpa1	0.210000	0.193333	Gpa1 / Gpa1 / Gpa1	3

Source: af2_receptor_rank_robustness.tsv. Maximum and mean rankings agree for 84/89 receptors; all three model weights select the same partner for 69/89. The original maximum-based winner counts are Gpa1 79, Gpa2 5 and Gpa3 5.

S6F Summary of all 267 receptor Gα combinations part 1

Seed 0; n = 3 model weights per combination. SD is the sample standard deviation of ipTM across model weights. Means and SDs are displayed to six decimal places.

Receptor	Partner	n	Min ipTM	Median	Mean	Max	SD
AOL_s00004g178.1	Gpa1	3	0.120000	0.520000	0.393333	0.540000	0.236925
AOL_s00004g178.1	Gpa2	3	0.160000	0.170000	0.183333	0.220000	0.032146
AOL_s00004g178.1	Gpa3	3	0.140000	0.220000	0.200000	0.240000	0.052915
AOL_s00004g22.1	Gpa1	3	0.610000	0.620000	0.620000	0.630000	0.010000
AOL_s00004g22.1	Gpa2	3	0.190000	0.230000	0.266667	0.380000	0.100167
AOL_s00004g22.1	Gpa3	3	0.310000	0.380000	0.393333	0.490000	0.090738
AOL_s00004g290.1	Gpa1	3	0.190000	0.420000	0.366667	0.490000	0.156950
AOL_s00004g290.1	Gpa2	3	0.150000	0.200000	0.243333	0.380000	0.120968
AOL_s00004g290.1	Gpa3	3	0.190000	0.260000	0.263333	0.340000	0.075056
AOL_s00006g420.1	Gpa1	3	0.510000	0.590000	0.583333	0.650000	0.070238
AOL_s00006g420.1	Gpa2	3	0.300000	0.640000	0.536667	0.670000	0.205508
AOL_s00006g420.1	Gpa3	3	0.510000	0.570000	0.573333	0.640000	0.065064
AOL_s00006g519.1	Gpa1	3	0.640000	0.670000	0.660000	0.670000	0.017321
AOL_s00006g519.1	Gpa2	3	0.380000	0.610000	0.540000	0.630000	0.138924
AOL_s00006g519.1	Gpa3	3	0.310000	0.350000	0.393333	0.520000	0.111505
AOL_s00007g229.1	Gpa1	3	0.650000	0.650000	0.650000	0.650000	0.000000
AOL_s00007g229.1	Gpa2	3	0.630000	0.640000	0.636667	0.640000	0.005774
AOL_s00007g229.1	Gpa3	3	0.550000	0.550000	0.560000	0.580000	0.017321
AOL_s00007g309.1	Gpa1	3	0.670000	0.690000	0.686667	0.700000	0.015275
AOL_s00007g309.1	Gpa2	3	0.180000	0.290000	0.323333	0.500000	0.162583
AOL_s00007g309.1	Gpa3	3	0.570000	0.600000	0.593333	0.610000	0.020817
AOL_s00007g90.1	Gpa1	3	0.430000	0.510000	0.486667	0.520000	0.049329
AOL_s00007g90.1	Gpa2	3	0.190000	0.230000	0.253333	0.340000	0.077675
AOL_s00007g90.1	Gpa3	3	0.300000	0.310000	0.346667	0.430000	0.072342
AOL_s00043g195.1	Gpa1	3	0.300000	0.620000	0.516667	0.630000	0.187705
AOL_s00043g195.1	Gpa2	3	0.180000	0.190000	0.290000	0.500000	0.181934
AOL_s00043g195.1	Gpa3	3	0.120000	0.190000	0.193333	0.270000	0.075056
AOL_s00043g223.1	Gpa1	3	0.560000	0.590000	0.583333	0.600000	0.020817
AOL_s00043g223.1	Gpa2	3	0.210000	0.210000	0.223333	0.250000	0.023094
AOL_s00043g223.1	Gpa3	3	0.230000	0.260000	0.380000	0.650000	0.234307
AOL_s00043g267.1	Gpa1	3	0.640000	0.670000	0.670000	0.700000	0.030000
AOL_s00043g267.1	Gpa2	3	0.220000	0.250000	0.273333	0.350000	0.068069
AOL_s00043g267.1	Gpa3	3	0.520000	0.590000	0.580000	0.630000	0.055678
AOL_s00043g324.1	Gpa1	3	0.520000	0.570000	0.553333	0.570000	0.028868
AOL_s00043g324.1	Gpa2	3	0.150000	0.200000	0.206667	0.270000	0.060277
AOL_s00043g324.1	Gpa3	3	0.230000	0.250000	0.266667	0.320000	0.047258
AOL_s00043g686.1	Gpa1	3	0.170000	0.470000	0.430000	0.650000	0.242487
AOL_s00043g686.1	Gpa2	3	0.210000	0.210000	0.213333	0.220000	0.005774
AOL_s00043g686.1	Gpa3	3	0.210000	0.230000	0.276667	0.390000	0.098658
AOL_s00043g705.1	Gpa1	3	0.170000	0.220000	0.206667	0.230000	0.032146
AOL_s00043g705.1	Gpa2	3	0.210000	0.240000	0.260000	0.330000	0.062450
AOL_s00043g705.1	Gpa3	3	0.190000	0.220000	0.223333	0.260000	0.035119
AOL_s00043g731.1	Gpa1	3	0.580000	0.620000	0.606667	0.620000	0.023094
AOL_s00043g731.1	Gpa2	3	0.240000	0.370000	0.346667	0.430000	0.097125
AOL_s00043g731.1	Gpa3	3	0.370000	0.430000	0.433333	0.500000	0.065064

Source: af2_pair_all_model_summary.tsv. All 267 summaries were recalculated from the 801 original scores and reconciled with the archived table.

S6F Summary of all 267 receptor Gα combinations part 2

Seed 0; n = 3 model weights per combination. SD is the sample standard deviation of ipTM across model weights. Means and SDs are displayed to six decimal places.

Receptor	Partner	n	Min ipTM	Median	Mean	Max	SD
AOL_s00043g738.2	Gpa1	3	0.590000	0.620000	0.620000	0.650000	0.030000
AOL_s00043g738.2	Gpa2	3	0.370000	0.430000	0.413333	0.440000	0.037859
AOL_s00043g738.2	Gpa3	3	0.360000	0.410000	0.400000	0.430000	0.036056
AOL_s00054g135.1	Gpa1	3	0.410000	0.690000	0.596667	0.690000	0.161658
AOL_s00054g135.1	Gpa2	3	0.260000	0.310000	0.296667	0.320000	0.032146
AOL_s00054g135.1	Gpa3	3	0.240000	0.290000	0.276667	0.300000	0.032146
AOL_s00054g150.1	Gpa1	3	0.610000	0.660000	0.646667	0.670000	0.032146
AOL_s00054g150.1	Gpa2	3	0.440000	0.530000	0.513333	0.570000	0.066583
AOL_s00054g150.1	Gpa3	3	0.300000	0.560000	0.473333	0.560000	0.150111
AOL_s00054g200.1	Gpa1	3	0.310000	0.560000	0.476667	0.560000	0.144338
AOL_s00054g200.1	Gpa2	3	0.180000	0.190000	0.193333	0.210000	0.015275
AOL_s00054g200.1	Gpa3	3	0.160000	0.180000	0.250000	0.410000	0.138924
AOL_s00054g223.1	Gpa1	3	0.490000	0.510000	0.506667	0.520000	0.015275
AOL_s00054g223.1	Gpa2	3	0.190000	0.210000	0.206667	0.220000	0.015275
AOL_s00054g223.1	Gpa3	3	0.190000	0.200000	0.223333	0.280000	0.049329
AOL_s00054g270.1	Gpa1	3	0.530000	0.540000	0.543333	0.560000	0.015275
AOL_s00054g270.1	Gpa2	3	0.330000	0.340000	0.376667	0.460000	0.072342
AOL_s00054g270.1	Gpa3	3	0.500000	0.530000	0.530000	0.560000	0.030000
AOL_s00054g291.1	Gpa1	3	0.140000	0.160000	0.163333	0.190000	0.025166
AOL_s00054g291.1	Gpa2	3	0.140000	0.170000	0.163333	0.180000	0.020817
AOL_s00054g291.1	Gpa3	3	0.150000	0.160000	0.173333	0.210000	0.032146
AOL_s00054g428.1	Gpa1	3	0.240000	0.260000	0.263333	0.290000	0.025166
AOL_s00054g428.1	Gpa2	3	0.190000	0.210000	0.206667	0.220000	0.015275
AOL_s00054g428.1	Gpa3	3	0.170000	0.210000	0.196667	0.210000	0.023094
AOL_s00054g605.1	Gpa1	3	0.480000	0.600000	0.560000	0.600000	0.069282
AOL_s00054g605.1	Gpa2	3	0.350000	0.570000	0.503333	0.590000	0.133167
AOL_s00054g605.1	Gpa3	3	0.380000	0.450000	0.436667	0.480000	0.051316
AOL_s00054g626.1	Gpa1	3	0.130000	0.140000	0.140000	0.150000	0.010000
AOL_s00054g626.1	Gpa2	3	0.160000	0.160000	0.163333	0.170000	0.005774
AOL_s00054g626.1	Gpa3	3	0.130000	0.170000	0.236667	0.410000	0.151438
AOL_s00054g63.1	Gpa1	3	0.200000	0.240000	0.280000	0.400000	0.105830
AOL_s00054g63.1	Gpa2	3	0.210000	0.220000	0.293333	0.450000	0.135769
AOL_s00054g63.1	Gpa3	3	0.190000	0.280000	0.293333	0.410000	0.110604
AOL_s00054g92.1	Gpa1	3	0.600000	0.630000	0.626667	0.650000	0.025166
AOL_s00054g92.1	Gpa2	3	0.230000	0.310000	0.293333	0.340000	0.056862
AOL_s00054g92.1	Gpa3	3	0.250000	0.290000	0.360000	0.540000	0.157162
AOL_s00075g112.1	Gpa1	3	0.680000	0.690000	0.690000	0.700000	0.010000
AOL_s00075g112.1	Gpa2	3	0.190000	0.420000	0.363333	0.480000	0.153080
AOL_s00075g112.1	Gpa3	3	0.330000	0.340000	0.426667	0.610000	0.158850
AOL_s00075g223.1	Gpa1	3	0.610000	0.640000	0.646667	0.690000	0.040415
AOL_s00075g223.1	Gpa2	3	0.590000	0.630000	0.620000	0.640000	0.026458
AOL_s00075g223.1	Gpa3	3	0.520000	0.570000	0.560000	0.590000	0.036056
AOL_s00076g157.1	Gpa1	3	0.170000	0.180000	0.180000	0.190000	0.010000
AOL_s00076g157.1	Gpa2	3	0.190000	0.190000	0.206667	0.240000	0.028868
AOL_s00076g157.1	Gpa3	3	0.190000	0.190000	0.213333	0.260000	0.040415

Source: af2_pair_all_model_summary.tsv. All 267 summaries were recalculated from the 801 original scores and reconciled with the archived table.

S6F Summary of all 267 receptor Gα combinations part 3

Seed 0; n = 3 model weights per combination. SD is the sample standard deviation of ipTM across model weights. Means and SDs are displayed to six decimal places.

Receptor	Partner	n	Min ipTM	Median	Mean	Max	SD
AOL_s00076g249.1	Gpa1	3	0.430000	0.450000	0.446667	0.460000	0.015275
AOL_s00076g249.1	Gpa2	3	0.170000	0.180000	0.180000	0.190000	0.010000
AOL_s00076g249.1	Gpa3	3	0.200000	0.210000	0.220000	0.250000	0.026458
AOL_s00076g286.1	Gpa1	3	0.380000	0.590000	0.536667	0.640000	0.137961
AOL_s00076g286.1	Gpa2	3	0.170000	0.380000	0.313333	0.390000	0.124231
AOL_s00076g286.1	Gpa3	3	0.240000	0.280000	0.286667	0.340000	0.050332
AOL_s00076g450.1	Gpa1	3	0.670000	0.690000	0.686667	0.700000	0.015275
AOL_s00076g450.1	Gpa2	3	0.260000	0.310000	0.303333	0.340000	0.040415
AOL_s00076g450.1	Gpa3	3	0.600000	0.620000	0.623333	0.650000	0.025166
AOL_s00076g465.1	Gpa1	3	0.370000	0.600000	0.526667	0.610000	0.135769
AOL_s00076g465.1	Gpa2	3	0.160000	0.240000	0.233333	0.300000	0.070238
AOL_s00076g465.1	Gpa3	3	0.230000	0.250000	0.253333	0.280000	0.025166
AOL_s00076g508.1	Gpa1	3	0.630000	0.680000	0.666667	0.690000	0.032146
AOL_s00076g508.1	Gpa2	3	0.170000	0.190000	0.186667	0.200000	0.015275
AOL_s00076g508.1	Gpa3	3	0.200000	0.270000	0.263333	0.320000	0.060277
AOL_s00076g553.1	Gpa1	3	0.640000	0.640000	0.646667	0.660000	0.011547
AOL_s00076g553.1	Gpa2	3	0.260000	0.300000	0.306667	0.360000	0.050332
AOL_s00076g553.1	Gpa3	3	0.500000	0.600000	0.570000	0.610000	0.060828
AOL_s00076g86.1	Gpa1	3	0.580000	0.610000	0.600000	0.610000	0.017321
AOL_s00076g86.1	Gpa2	3	0.190000	0.200000	0.333333	0.610000	0.239653
AOL_s00076g86.1	Gpa3	3	0.210000	0.220000	0.223333	0.240000	0.015275
AOL_s00078g446.1	Gpa1	3	0.170000	0.280000	0.266667	0.350000	0.090738
AOL_s00078g446.1	Gpa2	3	0.120000	0.150000	0.150000	0.180000	0.030000
AOL_s00078g446.1	Gpa3	3	0.220000	0.230000	0.263333	0.340000	0.066583
AOL_s00078g563.1	Gpa1	3	0.300000	0.590000	0.503333	0.620000	0.176730
AOL_s00078g563.1	Gpa2	3	0.260000	0.340000	0.346667	0.440000	0.090185
AOL_s00078g563.1	Gpa3	3	0.190000	0.200000	0.280000	0.450000	0.147309
AOL_s00078g598.1	Gpa1	3	0.660000	0.670000	0.673333	0.690000	0.015275
AOL_s00078g598.1	Gpa2	3	0.560000	0.620000	0.610000	0.650000	0.045826
AOL_s00078g598.1	Gpa3	3	0.480000	0.490000	0.496667	0.520000	0.020817
AOL_s00079g307.1	Gpa1	3	0.300000	0.520000	0.466667	0.580000	0.147422
AOL_s00079g307.1	Gpa2	3	0.200000	0.430000	0.360000	0.450000	0.138924
AOL_s00079g307.1	Gpa3	3	0.440000	0.470000	0.483333	0.540000	0.051316
AOL_s00079g452.1	Gpa1	3	0.600000	0.610000	0.616667	0.640000	0.020817
AOL_s00079g452.1	Gpa2	3	0.220000	0.230000	0.263333	0.340000	0.066583
AOL_s00079g452.1	Gpa3	3	0.340000	0.580000	0.506667	0.600000	0.144684
AOL_s00080g10.1	Gpa1	3	0.480000	0.540000	0.526667	0.560000	0.041633
AOL_s00080g10.1	Gpa2	3	0.220000	0.240000	0.240000	0.260000	0.020000
AOL_s00080g10.1	Gpa3	3	0.200000	0.380000	0.320000	0.380000	0.103923
AOL_s00080g416.1	Gpa1	3	0.520000	0.570000	0.560000	0.590000	0.036056
AOL_s00080g416.1	Gpa2	3	0.330000	0.380000	0.380000	0.430000	0.050000
AOL_s00080g416.1	Gpa3	3	0.370000	0.440000	0.423333	0.460000	0.047258
AOL_s00081g15.1	Gpa1	3	0.600000	0.610000	0.610000	0.620000	0.010000
AOL_s00081g15.1	Gpa2	3	0.420000	0.450000	0.450000	0.480000	0.030000
AOL_s00081g15.1	Gpa3	3	0.230000	0.260000	0.266667	0.310000	0.040415

Source: af2_pair_all_model_summary.tsv. All 267 summaries were recalculated from the 801 original scores and reconciled with the archived table.

S6F Summary of all 267 receptor Gα combinations part 4

Seed 0; n = 3 model weights per combination. SD is the sample standard deviation of ipTM across model weights. Means and SDs are displayed to six decimal places.

Receptor	Partner	n	Min ipTM	Median	Mean	Max	SD
AOL_s00081g30.1	Gpa1	3	0.250000	0.250000	0.306667	0.420000	0.098150
AOL_s00081g30.1	Gpa2	3	0.200000	0.220000	0.213333	0.220000	0.011547
AOL_s00081g30.1	Gpa3	3	0.240000	0.290000	0.316667	0.420000	0.092916
AOL_s00083g156.1	Gpa1	3	0.640000	0.650000	0.656667	0.680000	0.020817
AOL_s00083g156.1	Gpa2	3	0.200000	0.300000	0.286667	0.360000	0.080829
AOL_s00083g156.1	Gpa3	3	0.580000	0.600000	0.600000	0.620000	0.020000
AOL_s00097g260.1	Gpa1	3	0.240000	0.520000	0.436667	0.550000	0.170978
AOL_s00097g260.1	Gpa2	3	0.170000	0.210000	0.203333	0.230000	0.030551
AOL_s00097g260.1	Gpa3	3	0.170000	0.210000	0.210000	0.250000	0.040000
AOL_s00097g290.1	Gpa1	3	0.620000	0.650000	0.646667	0.670000	0.025166
AOL_s00097g290.1	Gpa2	3	0.210000	0.210000	0.210000	0.210000	0.000000
AOL_s00097g290.1	Gpa3	3	0.230000	0.310000	0.293333	0.340000	0.056862
AOL_s00097g309.3	Gpa1	3	0.590000	0.620000	0.610000	0.620000	0.017321
AOL_s00097g309.3	Gpa2	3	0.210000	0.340000	0.333333	0.450000	0.120139
AOL_s00097g309.3	Gpa3	3	0.330000	0.360000	0.366667	0.410000	0.040415
AOL_s00097g558.3	Gpa1	3	0.340000	0.400000	0.390000	0.430000	0.045826
AOL_s00097g558.3	Gpa2	3	0.150000	0.180000	0.176667	0.200000	0.025166
AOL_s00097g558.3	Gpa3	3	0.210000	0.230000	0.250000	0.310000	0.052915
AOL_s00097g69.1	Gpa1	3	0.170000	0.210000	0.216667	0.270000	0.050332
AOL_s00097g69.1	Gpa2	3	0.350000	0.460000	0.460000	0.570000	0.110000
AOL_s00097g69.1	Gpa3	3	0.190000	0.200000	0.200000	0.210000	0.010000
AOL_s00109g63.1	Gpa1	3	0.500000	0.570000	0.553333	0.590000	0.047258
AOL_s00109g63.1	Gpa2	3	0.240000	0.250000	0.306667	0.430000	0.106927
AOL_s00109g63.1	Gpa3	3	0.300000	0.300000	0.320000	0.360000	0.034641
AOL_s00109g8.1	Gpa1	3	0.580000	0.590000	0.590000	0.600000	0.010000
AOL_s00109g8.1	Gpa2	3	0.160000	0.170000	0.170000	0.180000	0.010000
AOL_s00109g8.1	Gpa3	3	0.250000	0.290000	0.366667	0.560000	0.168622
AOL_s00110g16.1	Gpa1	3	0.480000	0.590000	0.553333	0.590000	0.063509
AOL_s00110g16.1	Gpa2	3	0.170000	0.370000	0.306667	0.380000	0.118462
AOL_s00110g16.1	Gpa3	3	0.160000	0.250000	0.263333	0.380000	0.110604
AOL_s00110g307.1	Gpa1	3	0.640000	0.650000	0.653333	0.670000	0.015275
AOL_s00110g307.1	Gpa2	3	0.600000	0.610000	0.616667	0.640000	0.020817
AOL_s00110g307.1	Gpa3	3	0.590000	0.620000	0.610000	0.620000	0.017321
AOL_s00110g337.1	Gpa1	3	0.460000	0.520000	0.510000	0.550000	0.045826
AOL_s00110g337.1	Gpa2	3	0.190000	0.260000	0.300000	0.450000	0.134536
AOL_s00110g337.1	Gpa3	3	0.220000	0.220000	0.243333	0.290000	0.040415
AOL_s00140g54.6	Gpa1	3	0.630000	0.640000	0.643333	0.660000	0.015275
AOL_s00140g54.6	Gpa2	3	0.190000	0.260000	0.236667	0.260000	0.040415
AOL_s00140g54.6	Gpa3	3	0.570000	0.580000	0.580000	0.590000	0.010000
AOL_s00169g110.1	Gpa1	3	0.600000	0.620000	0.616667	0.630000	0.015275
AOL_s00169g110.1	Gpa2	3	0.140000	0.350000	0.310000	0.440000	0.153948
AOL_s00169g110.1	Gpa3	3	0.170000	0.340000	0.323333	0.460000	0.145717
AOL_s00169g261.1	Gpa1	3	0.650000	0.670000	0.666667	0.680000	0.015275
AOL_s00169g261.1	Gpa2	3	0.180000	0.280000	0.276667	0.370000	0.095044
AOL_s00169g261.1	Gpa3	3	0.560000	0.580000	0.573333	0.580000	0.011547

Source: af2_pair_all_model_summary.tsv. All 267 summaries were recalculated from the 801 original scores and reconciled with the archived table.

S6F Summary of all 267 receptor Gα combinations part 5

Seed 0; n = 3 model weights per combination. SD is the sample standard deviation of ipTM across model weights. Means and SDs are displayed to six decimal places.

Receptor	Partner	n	Min ipTM	Median	Mean	Max	SD
AOL_s00170g20.1	Gpa1	3	0.480000	0.520000	0.523333	0.570000	0.045092
AOL_s00170g20.1	Gpa2	3	0.240000	0.320000	0.310000	0.370000	0.065574
AOL_s00170g20.1	Gpa3	3	0.160000	0.200000	0.206667	0.260000	0.050332
AOL_s00170g54.1	Gpa1	3	0.640000	0.640000	0.643333	0.650000	0.005774
AOL_s00170g54.1	Gpa2	3	0.300000	0.350000	0.343333	0.380000	0.040415
AOL_s00170g54.1	Gpa3	3	0.300000	0.370000	0.380000	0.470000	0.085440
AOL_s00170g99.1	Gpa1	3	0.440000	0.450000	0.486667	0.570000	0.072342
AOL_s00170g99.1	Gpa2	3	0.270000	0.270000	0.313333	0.400000	0.075056
AOL_s00170g99.1	Gpa3	3	0.230000	0.260000	0.276667	0.340000	0.056862
AOL_s00173g137.1	Gpa1	3	0.640000	0.650000	0.646667	0.650000	0.005774
AOL_s00173g137.1	Gpa2	3	0.210000	0.210000	0.246667	0.320000	0.063509
AOL_s00173g137.1	Gpa3	3	0.200000	0.260000	0.276667	0.370000	0.086217
AOL_s00173g258.2	Gpa1	3	0.420000	0.470000	0.463333	0.500000	0.040415
AOL_s00173g258.2	Gpa2	3	0.490000	0.560000	0.550000	0.600000	0.055678
AOL_s00173g258.2	Gpa3	3	0.480000	0.490000	0.496667	0.520000	0.020817
AOL_s00173g37.1	Gpa1	3	0.650000	0.670000	0.666667	0.680000	0.015275
AOL_s00173g37.1	Gpa2	3	0.230000	0.280000	0.266667	0.290000	0.032146
AOL_s00173g37.1	Gpa3	3	0.520000	0.550000	0.546667	0.570000	0.025166
AOL_s00176g24.1	Gpa1	3	0.140000	0.540000	0.420000	0.580000	0.243311
AOL_s00176g24.1	Gpa2	3	0.190000	0.250000	0.233333	0.260000	0.037859
AOL_s00176g24.1	Gpa3	3	0.160000	0.270000	0.246667	0.310000	0.077675
AOL_s00176g42.1	Gpa1	3	0.290000	0.350000	0.336667	0.370000	0.041633
AOL_s00176g42.1	Gpa2	3	0.200000	0.230000	0.223333	0.240000	0.020817
AOL_s00176g42.1	Gpa3	3	0.210000	0.220000	0.220000	0.230000	0.010000
AOL_s00188g124.1	Gpa1	3	0.180000	0.200000	0.193333	0.200000	0.011547
AOL_s00188g124.1	Gpa2	3	0.150000	0.170000	0.166667	0.180000	0.015275
AOL_s00188g124.1	Gpa3	3	0.150000	0.160000	0.160000	0.170000	0.010000
AOL_s00188g142.1	Gpa1	3	0.700000	0.700000	0.710000	0.730000	0.017321
AOL_s00188g142.1	Gpa2	3	0.610000	0.640000	0.633333	0.650000	0.020817
AOL_s00188g142.1	Gpa3	3	0.530000	0.620000	0.600000	0.650000	0.062450
AOL_s00188g284.1	Gpa1	3	0.620000	0.660000	0.646667	0.660000	0.023094
AOL_s00188g284.1	Gpa2	3	0.190000	0.370000	0.333333	0.440000	0.128970
AOL_s00188g284.1	Gpa3	3	0.380000	0.540000	0.490000	0.550000	0.095394
AOL_s00188g320.1	Gpa1	3	0.150000	0.540000	0.413333	0.550000	0.228108
AOL_s00188g320.1	Gpa2	3	0.210000	0.220000	0.223333	0.240000	0.015275
AOL_s00188g320.1	Gpa3	3	0.120000	0.310000	0.253333	0.330000	0.115902
AOL_s00188g342.1	Gpa1	3	0.600000	0.600000	0.610000	0.630000	0.017321
AOL_s00188g342.1	Gpa2	3	0.240000	0.330000	0.343333	0.460000	0.110604
AOL_s00188g342.1	Gpa3	3	0.320000	0.330000	0.336667	0.360000	0.020817
AOL_s00188g346.1	Gpa1	3	0.210000	0.500000	0.406667	0.510000	0.170392
AOL_s00188g346.1	Gpa2	3	0.200000	0.210000	0.223333	0.260000	0.032146
AOL_s00188g346.1	Gpa3	3	0.220000	0.230000	0.236667	0.260000	0.020817
AOL_s00193g107.1	Gpa1	3	0.640000	0.640000	0.650000	0.670000	0.017321
AOL_s00193g107.1	Gpa2	3	0.320000	0.450000	0.420000	0.490000	0.088882
AOL_s00193g107.1	Gpa3	3	0.600000	0.610000	0.613333	0.630000	0.015275

Source: af2_pair_all_model_summary.tsv. All 267 summaries were recalculated from the 801 original scores and reconciled with the archived table.

S6F Summary of all 267 receptor Gα combinations part 6

Seed 0; n = 3 model weights per combination. SD is the sample standard deviation of ipTM across model weights. Means and SDs are displayed to six decimal places.

Receptor	Partner	n	Min ipTM	Median	Mean	Max	SD
AOL_s00210g135.2	Gpa1	3	0.600000	0.610000	0.610000	0.620000	0.010000
AOL_s00210g135.2	Gpa2	3	0.320000	0.430000	0.416667	0.500000	0.090738
AOL_s00210g135.2	Gpa3	3	0.240000	0.370000	0.373333	0.510000	0.135031
AOL_s00210g301.1	Gpa1	3	0.680000	0.690000	0.686667	0.690000	0.005774
AOL_s00210g301.1	Gpa2	3	0.210000	0.220000	0.220000	0.230000	0.010000
AOL_s00210g301.1	Gpa3	3	0.300000	0.520000	0.473333	0.600000	0.155349
AOL_s00210g381.1	Gpa1	3	0.150000	0.200000	0.313333	0.590000	0.240901
AOL_s00210g381.1	Gpa2	3	0.170000	0.240000	0.216667	0.240000	0.040415
AOL_s00210g381.1	Gpa3	3	0.170000	0.180000	0.180000	0.190000	0.010000
AOL_s00210g385.1	Gpa1	3	0.580000	0.590000	0.586667	0.590000	0.005774
AOL_s00210g385.1	Gpa2	3	0.250000	0.330000	0.320000	0.380000	0.065574
AOL_s00210g385.1	Gpa3	3	0.230000	0.260000	0.276667	0.340000	0.056862
AOL_s00215g252.1	Gpa1	3	0.410000	0.480000	0.463333	0.500000	0.047258
AOL_s00215g252.1	Gpa2	3	0.200000	0.220000	0.250000	0.330000	0.070000
AOL_s00215g252.1	Gpa3	3	0.190000	0.200000	0.220000	0.270000	0.043589
AOL_s00215g301.1	Gpa1	3	0.560000	0.580000	0.586667	0.620000	0.030551
AOL_s00215g301.1	Gpa2	3	0.190000	0.190000	0.193333	0.200000	0.005774
AOL_s00215g301.1	Gpa3	3	0.200000	0.240000	0.256667	0.330000	0.066583
AOL_s00215g417.1	Gpa1	3	0.150000	0.510000	0.393333	0.520000	0.210792
AOL_s00215g417.1	Gpa2	3	0.170000	0.250000	0.240000	0.300000	0.065574
AOL_s00215g417.1	Gpa3	3	0.130000	0.200000	0.196667	0.260000	0.065064
AOL_s00215g444.1	Gpa1	3	0.620000	0.630000	0.630000	0.640000	0.010000
AOL_s00215g444.1	Gpa2	3	0.170000	0.450000	0.393333	0.560000	0.201080
AOL_s00215g444.1	Gpa3	3	0.300000	0.540000	0.460000	0.540000	0.138564
AOL_s00215g562.1	Gpa1	3	0.300000	0.310000	0.333333	0.390000	0.049329
AOL_s00215g562.1	Gpa2	3	0.170000	0.180000	0.193333	0.230000	0.032146
AOL_s00215g562.1	Gpa3	3	0.120000	0.400000	0.306667	0.400000	0.161658
AOL_s00215g603.1	Gpa1	3	0.540000	0.580000	0.580000	0.620000	0.040000
AOL_s00215g603.1	Gpa2	3	0.230000	0.500000	0.413333	0.510000	0.158850
AOL_s00215g603.1	Gpa3	3	0.190000	0.420000	0.353333	0.450000	0.142244
AOL_s00215g796.1	Gpa1	3	0.600000	0.620000	0.613333	0.620000	0.011547
AOL_s00215g796.1	Gpa2	3	0.200000	0.200000	0.233333	0.300000	0.057735
AOL_s00215g796.1	Gpa3	3	0.370000	0.480000	0.446667	0.490000	0.066583
AOL_s00215g906.3	Gpa1	3	0.590000	0.600000	0.610000	0.640000	0.026458
AOL_s00215g906.3	Gpa2	3	0.180000	0.230000	0.266667	0.390000	0.109697
AOL_s00215g906.3	Gpa3	3	0.470000	0.510000	0.506667	0.540000	0.035119
AOL_s00215g915.1	Gpa1	3	0.280000	0.340000	0.360000	0.460000	0.091652
AOL_s00215g915.1	Gpa2	3	0.110000	0.190000	0.190000	0.270000	0.080000
AOL_s00215g915.1	Gpa3	3	0.170000	0.200000	0.210000	0.260000	0.045826
AOL_s00215g924.1	Gpa1	3	0.360000	0.490000	0.456667	0.520000	0.085049
AOL_s00215g924.1	Gpa2	3	0.130000	0.200000	0.190000	0.240000	0.055678
AOL_s00215g924.1	Gpa3	3	0.240000	0.240000	0.263333	0.310000	0.040415

Source: af2_pair_all_model_summary.tsv. All 267 summaries were recalculated from the 801 original scores and reconciled with the archived table.

S6G Orthogroup mapping for the 89 screened proteins part 1

Mappings are extracted from the genome-wide orthogroup table for the 89 proteins in the structural screen. AOL and TWF counts refer to complete orthogroup membership. TWF identifiers shown here are orthogroup members; an explicit orthologue call is reported as a group-level flag, not as proof of every possible protein pair.

AOL protein	Orthogroup	AOL / TWF n	Relation	TWF protein members	Explicit call / pairs
AOL_s00004g178.1	OG0001754	1 / 1	one-to-one	EYR41_009763	no / 0
AOL_s00004g22.1	OG0010318	1 / 1	one-to-one	EYR41_009924	no / 0
AOL_s00004g290.1	OG0010109	1 / 1	one-to-one	EYR41_009642	no / 0
AOL_s00006g420.1	OG0003011	1 / 1	one-to-one	EYR41_004122	no / 0
AOL_s00006g519.1	OG0001631	1 / 1	one-to-one	EYR41_004233	no / 0
AOL_s00007g229.1	OG0000177	1 / 3	one-to-many	EYR41_011115; EYR41_011115_1; EYR41_011115_2	yes / 3
AOL_s00007g309.1	OG0008623	1 / 1	one-to-one	EYR41_011201	no / 0
AOL_s00007g90.1	OG0008782	1 / 1	one-to-one	EYR41_010973	no / 0
AOL_s00043g195.1	OG0000279	1 / 2	one-to-many	EYR41_005717; EYR41_005717_1	yes / 2
AOL_s00043g223.1	OG0001311	1 / 1	one-to-one	EYR41_005746	no / 0
AOL_s00043g267.1	OG0000548	1 / 2	one-to-many	EYR41_005791; EYR41_005791_1	yes / 2
AOL_s00043g324.1	OG0004248	1 / 1	one-to-one	EYR41_005855	no / 0
AOL_s00043g686.1	OG0004548	1 / 1	one-to-one	EYR41_006290	no / 0
AOL_s00043g705.1	OG0004564	1 / 1	one-to-one	EYR41_006308	no / 0
AOL_s00043g731.1	OG0004585	1 / 1	one-to-one	EYR41_006336	no / 0
AOL_s00043g738.2	OG0004591	1 / 1	one-to-one	EYR41_006344	no / 0
AOL_s00054g135.1	OG0002543	1 / 1	one-to-one	EYR41_003539	no / 0
AOL_s00054g150.1	OG0000376	1 / 2	one-to-many	EYR41_002638; EYR41_003524	yes / 2
AOL_s00054g200.1	OG0002492	1 / 1	one-to-one	EYR41_003469	no / 0
AOL_s00054g223.1	OG0002470	1 / 1	one-to-one	EYR41_003442	no / 0
AOL_s00054g270.1	OG0002429	1 / 1	one-to-one	EYR41_003390	no / 0
AOL_s00054g291.1	OG0002413	1 / 1	one-to-one	EYR41_003367	no / 0
AOL_s00054g428.1	OG0002295	1 / 1	one-to-one	EYR41_003224	no / 0
AOL_s00054g605.1	OG0002157	1 / 1	one-to-one	EYR41_003042	no / 0
AOL_s00054g626.1	OG0002142	1 / 1	one-to-one	EYR41_003020	no / 0
AOL_s00054g63.1	OG0000021	1 / 4	one-to-many	EYR41_003613; EYR41_003613_1; EYR41_003613_2; EYR41_003613_3	yes / 4
AOL_s00054g92.1	OG0000092	1 / 3	one-to-many	EYR41_003584; EYR41_003584_1; EYR41_003584_2	yes / 3
AOL_s00075g112.1	OG0008308	1 / 1	one-to-one	EYR41_011616	no / 0
AOL_s00075g223.1	OG0008391	1 / 1	one-to-one	EYR41_011505	no / 0
AOL_s00076g157.1	OG0003544	1 / 1	one-to-one	EYR41_004917	no / 0

Source: orthology/AOL_TWF_orthogroup_mapping.tsv. The full table has 10,828 orthogroup records and is retained with the companion source files.

S6G Orthogroup mapping for the 89 screened proteins part 2

Mappings are extracted from the genome-wide orthogroup table for the 89 proteins in the structural screen. AOL and TWF counts refer to complete orthogroup membership. TWF identifiers shown here are orthogroup members; an explicit orthologue call is reported as a group-level flag, not as proof of every possible protein pair.

AOL protein	Orthogroup	AOL / TWF n	Relation	TWF protein members	Explicit call / pairs
AOL_s00076g249.1	OG0003617	1 / 1	one-to-one	EYR41_005010	no / 0
AOL_s00076g286.1	OG0003650	1 / 1	one-to-one	EYR41_005056	no / 0
AOL_s00076g450.1	OG0003766	1 / 1	one-to-one	EYR41_005221	no / 0
AOL_s00076g465.1	OG0001673	1 / 1	one-to-one	EYR41_005237	no / 0
AOL_s00076g508.1	OG0003812	1 / 1	one-to-one	EYR41_005283	no / 0
AOL_s00076g553.1	OG0000522	1 / 2	one-to-many	EYR41_005331; EYR41_005331_1	yes / 2
AOL_s00076g86.1	OG0003483	1 / 1	one-to-one	EYR41_004841	no / 0
AOL_s00078g446.1	OG0006563	1 / 1	one-to-one	EYR41_008678	no / 0
AOL_s00078g563.1	OG0006471	1 / 1	one-to-one	EYR41_008805	no / 0
AOL_s00078g598.1	OG0001632	1 / 1	one-to-one	EYR41_008840	no / 0
AOL_s00079g307.1	OG0008072	1 / 1	one-to-one	EYR41_007468	no / 0
AOL_s00079g452.1	OG0008192	1 / 1	one-to-one	EYR41_007625	no / 0
AOL_s00080g10.1	OG0001647	1 / 1	one-to-one	EYR41_001767	no / 0
AOL_s00080g416.1	OG0004675	1 / 1	one-to-one	EYR41_002191	no / 0
AOL_s00081g15.1	OG0007307	1 / 1	one-to-one	EYR41_007715	no / 0
AOL_s00081g30.1	OG0007293	1 / 1	one-to-one	EYR41_007731	no / 0
AOL_s00083g156.1	OG0005460	1 / 1	one-to-one	EYR41_001158	no / 0
AOL_s00097g260.1	OG0007520	1 / 1	one-to-one	EYR41_006715	no / 0
AOL_s00097g290.1	OG0007542	1 / 1	one-to-one	EYR41_006747	no / 0
AOL_s00097g309.3	OG0007557	1 / 1	one-to-one	EYR41_006767	no / 0
AOL_s00097g558.3	OG0007755	1 / 1	one-to-one	EYR41_007055	no / 0
AOL_s00097g69.1	OG0000828	1 / 2	one-to-many	EYR41_006517; EYR41_006517_1	yes / 2
AOL_s00109g63.1	OG0009176	1 / 1	one-to-one	EYR41_010407	no / 0
AOL_s00109g8.1	OG0009131	1 / 1	one-to-one	EYR41_010470	no / 0
AOL_s00110g16.1	OG0001674	1 / 1	one-to-one	EYR41_012081	no / 0
AOL_s00110g307.1	OG0010767	1 / 1	one-to-one	EYR41_011781	no / 0
AOL_s00110g337.1	OG0000234	1 / 2	one-to-many	EYR41_011744; EYR41_011744_1	yes / 2
AOL_s00140g54.6	OG0000183	1 / 3	one-to-many	EYR41_010002; EYR41_010002_1; EYR41_010002_2	yes / 3
AOL_s00169g110.1	OG0010449	1 / 1	one-to-one	EYR41_002387	no / 0
AOL_s00169g261.1	OG0010339	1 / 1	one-to-one	EYR41_002235	no / 0

Source: orthology/AOL_TWF_orthogroup_mapping.tsv. The full table has 10,828 orthogroup records and is retained with the companion source files.

S6G Orthogroup mapping for the 89 screened proteins part 3

Mappings are extracted from the genome-wide orthogroup table for the 89 proteins in the structural screen. AOL and TWF counts refer to complete orthogroup membership. TWF identifiers shown here are orthogroup members; an explicit orthologue call is reported as a group-level flag, not as proof of every possible protein pair.

AOL protein	Orthogroup	AOL / TWF n	Relation	TWF protein members	Explicit call / pairs
AOL_s00170g20.1	OG0006367	1 / 1	one-to-one	EYR41_002539	no / 0
AOL_s00170g54.1	OG0006393	1 / 1	one-to-one	EYR41_002574	no / 0
AOL_s00170g99.1	OG0006433	1 / 1	one-to-one	EYR41_002623	no / 0
AOL_s00173g137.1	OG0003209	1 / 1	one-to-one	EYR41_004453	no / 0
AOL_s00173g258.2	OG0001648	1 / 1	one-to-one	EYR41_004587	no / 0
AOL_s00173g37.1	OG0003133	1 / 1	one-to-one	EYR41_004340	no / 0
AOL_s00176g24.1	OG0009374	1 / 1	one-to-one	EYR41_010142	no / 0
AOL_s00176g42.1	OG0009389	1 / 1	one-to-one	EYR41_010125	no / 0
AOL_s00188g124.1	OG0001041	1 / 2	one-to-many	EYR41_009112; EYR41_009112_1	yes / 2
AOL_s00188g142.1	OG0009675	1 / 1	one-to-one	EYR41_009087	no / 0
AOL_s00188g284.1	OG0009568	1 / 1	one-to-one	EYR41_008941	no / 0
AOL_s00188g320.1	OG0009541	1 / 1	one-to-one	EYR41_008902	no / 0
AOL_s00188g342.1	OG0009527	1 / 1	one-to-one	EYR41_008882	no / 0
AOL_s00188g346.1	OG0001029	1 / 2	one-to-many	EYR41_008878; EYR41_008878_1	yes / 2
AOL_s00193g107.1	OG0005081	1 / 1	one-to-one	EYR41_001665	no / 0
AOL_s00210g135.2	OG0009030	1 / 1	one-to-one	EYR41_010612	no / 0
AOL_s00210g301.1	OG0000153	3 / 1	many-to-one	EYR41_010777	yes / 3
AOL_s00210g381.1	OG0000235	1 / 2	one-to-many	EYR41_003116; EYR41_010857	yes / 2
AOL_s00210g385.1	OG0008858	1 / 1	one-to-one	EYR41_010861	no / 0
AOL_s00215g252.1	OG0001310	1 / 1	one-to-one	EYR41_000734	no / 0
AOL_s00215g301.1	OG0000147	1 / 3	one-to-many	EYR41_000680; EYR41_000680_1; EYR41_000680_2	yes / 3
AOL_s00215g417.1	OG0001753	1 / 1	one-to-one	EYR41_000552	no / 0
AOL_s00215g444.1	OG0005950	1 / 1	one-to-one	EYR41_000519	no / 0
AOL_s00215g562.1	OG0006047	1 / 1	one-to-one	EYR41_000394	no / 0
AOL_s00215g603.1	OG0006087	1 / 1	one-to-one	EYR41_000346	no / 0
AOL_s00215g796.1	OG0006247	1 / 1	one-to-one	EYR41_000145	no / 0
AOL_s00215g906.3	OG0006334	1 / 1	one-to-one	EYR41_000025	no / 0
AOL_s00215g915.1	OG0006342	1 / 1	one-to-one	EYR41_000013	no / 0
AOL_s00215g924.1	OG0001543	1 / 1	one-to-one	EYR41_000003	no / 0

Source: orthology/AOL_TWF_orthogroup_mapping.tsv. The full table has 10,828 orthogroup records and is retained with the companion source files.

S6H Source files and reproducibility records

Data S6 source files are archived in Data_S6_Structure.zip. Relative paths below refer to its root. The original scoring and mapping tables are unchanged. This Word document reproduces the complete numeric score dataset and candidate mapping subset; atomic coordinates, alignments, sequences and scripts retain their native formats in the source archive.

Relative file path	Bytes	Content
README.txt	551	Native source or analysis file
af2_all_model_scores_clean.tsv	107865	801 original scores
af2_focal_pair_sensitivity.tsv	2182	12 focal pair summaries
af2_focal_seed_pair_ranges.tsv	671	12 pair-by-seed ranges
af2_focal_seed_sensitivity.tsv	474	4 receptor-by-seed ranking summaries
af2_focal_seed_summary.json	1446	Native source or analysis file
af2_focal_two_seed_scores.tsv	5852	36 focal scores, including 18 reused seed-0 scores
af2_pair_all_model_summary.tsv	20525	267 pair summaries
af2_receptor_rank_robustness.tsv	9075	89 receptor ranking records
analyse_focal_seed.py	3073	Native source or analysis file
config.json	1127	Native source or analysis file
inputs/af2_all_model_scores.tsv	110280	Native source or analysis file
inputs/af2_focal_seed101_scores.tsv	2562	Native source or analysis file
inputs/focal_a3m/AOL_s00007g309_1__AOGpa1.a3m	13006553	Native source or analysis file
inputs/focal_a3m/AOL_s00007g309_1__AOGpa2.a3m	11788942	Native source or analysis file
inputs/focal_a3m/AOL_s00007g309_1__AOGpa3.a3m	11379207	Native source or analysis file
inputs/focal_a3m/AOL_s00176g42_1__AOGpa1.a3m	13600722	Native source or analysis file
inputs/focal_a3m/AOL_s00176g42_1__AOGpa2.a3m	12271142	Native source or analysis file
inputs/focal_a3m/AOL_s00176g42_1__AOGpa3.a3m	11829981	Native source or analysis file
orthology/AOL_TWF_orthogroup_mapping.tsv	681174	10828 genome-wide orthogroup records
orthology/Arthrobotrys_oligospora_GPCR_new.fasta	44929	Native source or analysis file
orthology/Arthrobotrys_oligospora_TWF_GPCR.fasta	38902	Native source or analysis file
orthology/Galpha.fasta	1091	Native source or analysis file
orthology/build_id_mapping.py	10908	Native source or analysis file
orthology/idmapping_AOL.xlsx	36600	Native source or analysis file
reanalyse_computation.py	11575	Native source or analysis file

The 12 representative PDB coordinate files are itemized in S6D. Their total size is 5999346 bytes. Six focal A3M alignments are retained. The three FASTA files retain the two strain GPCR sequence sets and the Gα sequence set.

Original score basename rule

<job>_scores_rank_<rank3>_alphafold2_multimer_v3_model_<weight>_seed_<seed3>.json

The rule reproduces every original score-file identifier. The <job>, model weight, rank and seed values fully identify each tabulated score. The source ranking_confidence column is blank for all original and seed-101 input records. No ranking-confidence value has been added.

Analysis scripts and scope

reanalyse_computation.py, analyse_focal_seed.py and orthology/build_id_mapping.py are retained as provenance records. Their relative paths reflect the original analysis project; running them requires the corresponding original project inputs and layout. The Word tables were checked directly against the archived numeric data. No structural prediction was rerun for this document.

Source archive SHA256 62309b82e7f7039cfc61fe57c2a346a98cb687dca42c67edb2544cc4976cb0ff

S6I Complete original model scores part 1

All rows use seed 0. One row represents one receptor–Gα combination; W1, W2 and W3 denote the three model weights. Each block gives archived rank, ipTM, pTM and maximum PAE in Å. Together, these 267 rows reproduce all 801 original models without averaging.

Receptor	Partner	W1 Rank	W1 ipTM	W1 pTM	W1 Max PAE Å	W2 Rank	W2 ipTM	W2 pTM	W2 Max PAE Å	W3 Rank	W3 ipTM	W3 pTM	W3 Max PAE Å
AOL_s00004g178.1	Gpa1	2	0.52	0.68	31.609375	1	0.54	0.68	31.671875	3	0.12	0.48	31.71875
AOL_s00004g178.1	Gpa2	3	0.16	0.51	31.71875	1	0.22	0.54	31.71875	2	0.17	0.51	31.71875
AOL_s00004g178.1	Gpa3	1	0.24	0.49	31.703125	2	0.22	0.52	31.6875	3	0.14	0.49	31.703125
AOL_s00004g22.1	Gpa1	2	0.62	0.63	31.65625	1	0.63	0.66	31.6875	3	0.61	0.64	31.703125
AOL_s00004g22.1	Gpa2	3	0.19	0.47	31.703125	2	0.23	0.49	31.71875	1	0.38	0.52	31.703125
AOL_s00004g22.1	Gpa3	3	0.31	0.52	31.6875	2	0.38	0.55	31.703125	1	0.49	0.59	31.703125
AOL_s00004g290.1	Gpa1	2	0.42	0.54	31.671875	1	0.49	0.55	31.703125	3	0.19	0.42	31.71875
AOL_s00004g290.1	Gpa2	1	0.38	0.51	31.671875	3	0.15	0.41	31.734375	2	0.2	0.42	31.703125
AOL_s00004g290.1	Gpa3	3	0.19	0.45	31.71875	2	0.26	0.46	31.703125	1	0.34	0.48	31.71875
AOL_s00006g420.1	Gpa1	2	0.59	0.53	31.703125	1	0.65	0.55	31.71875	3	0.51	0.5	31.703125
AOL_s00006g420.1	Gpa2	2	0.64	0.55	31.703125	1	0.67	0.54	31.703125	3	0.3	0.42	31.703125
AOL_s00006g420.1	Gpa3	2	0.57	0.52	31.703125	1	0.64	0.51	31.703125	3	0.51	0.5	31.703125
AOL_s00006g519.1	Gpa1	1	0.67	0.67	31.65625	2	0.67	0.67	31.6875	3	0.64	0.65	31.6875
AOL_s00006g519.1	Gpa2	1	0.63	0.66	31.671875	3	0.38	0.55	31.703125	2	0.61	0.64	31.703125
AOL_s00006g519.1	Gpa3	3	0.31	0.54	31.6875	2	0.35	0.55	31.6875	1	0.52	0.6	31.703125
AOL_s00007g229.1	Gpa1	2	0.65	0.67	31.6875	1	0.65	0.67	31.71875	3	0.65	0.66	31.703125
AOL_s00007g229.1	Gpa2	3	0.63	0.66	31.671875	1	0.64	0.66	31.703125	2	0.64	0.66	31.71875
AOL_s00007g229.1	Gpa3	3	0.55	0.62	31.6875	1	0.58	0.63	31.71875	2	0.55	0.61	31.6875
AOL_s00007g309.1	Gpa1	1	0.7	0.73	31.546875	2	0.69	0.72	31.65625	3	0.67	0.7	31.6875
AOL_s00007g309.1	Gpa2	2	0.29	0.49	31.640625	1	0.5	0.59	31.6875	3	0.18	0.47	31.71875
AOL_s00007g309.1	Gpa3	2	0.6	0.65	31.609375	1	0.61	0.65	31.640625	3	0.57	0.63	31.65625
AOL_s00007g90.1	Gpa1	1	0.51	0.57	31.671875	3	0.43	0.52	31.6875	2	0.52	0.55	31.703125
AOL_s00007g90.1	Gpa2	2	0.23	0.45	31.671875	1	0.34	0.46	31.703125	3	0.19	0.43	31.71875
AOL_s00007g90.1	Gpa3	2	0.3	0.48	31.703125	1	0.43	0.5	31.703125	3	0.31	0.46	31.6875
AOL_s00043g195.1	Gpa1	1	0.63	0.71	31.609375	2	0.62	0.71	31.671875	3	0.3	0.56	31.6875
AOL_s00043g195.1	Gpa2	2	0.19	0.5	31.65625	1	0.5	0.64	31.6875	3	0.18	0.5	31.6875
AOL_s00043g195.1	Gpa3	2	0.19	0.5	31.65625	1	0.27	0.54	31.671875	3	0.12	0.47	31.6875

Source: af2_all_model_scores_clean.tsv. Original score basenames are reconstructed exactly using the rule in S6H. Global maximum PAE values are reproduced at source precision.

S6I Complete original model scores part 2

All rows use seed 0. One row represents one receptor–Gα combination; W1, W2 and W3 denote the three model weights. Each block gives archived rank, ipTM, pTM and maximum PAE in Å. Together, these 267 rows reproduce all 801 original models without averaging.

Receptor	Partner	W1 Rank	W1 ipTM	W1 pTM	W1 Max PAE Å	W2 Rank	W2 ipTM	W2 pTM	W2 Max PAE Å	W3 Rank	W3 ipTM	W3 pTM	W3 Max PAE Å
AOL_s00043g223.1	Gpa1	3	0.56	0.64	31.671875	1	0.6	0.66	31.71875	2	0.59	0.65	31.703125
AOL_s00043g223.1	Gpa2	3	0.21	0.45	31.6875	1	0.25	0.48	31.703125	2	0.21	0.47	31.71875
AOL_s00043g223.1	Gpa3	3	0.23	0.47	31.6875	2	0.26	0.48	31.71875	1	0.65	0.68	31.71875
AOL_s00043g267.1	Gpa1	2	0.67	0.66	31.640625	1	0.7	0.68	31.6875	3	0.64	0.64	31.71875
AOL_s00043g267.1	Gpa2	3	0.22	0.47	31.640625	1	0.35	0.51	31.6875	2	0.25	0.47	31.703125
AOL_s00043g267.1	Gpa3	3	0.52	0.6	31.671875	2	0.59	0.62	31.703125	1	0.63	0.63	31.703125
AOL_s00043g324.1	Gpa1	1	0.57	0.68	31.65625	2	0.57	0.66	31.6875	3	0.52	0.59	31.6875
AOL_s00043g324.1	Gpa2	2	0.2	0.51	31.671875	1	0.27	0.52	31.703125	3	0.15	0.49	31.703125
AOL_s00043g324.1	Gpa3	2	0.25	0.54	31.671875	1	0.32	0.57	31.6875	3	0.23	0.49	31.703125
AOL_s00043g686.1	Gpa1	1	0.65	0.69	31.65625	2	0.47	0.61	31.71875	3	0.17	0.44	31.71875
AOL_s00043g686.1	Gpa2	2	0.21	0.46	31.703125	1	0.22	0.47	31.703125	3	0.21	0.46	31.703125
AOL_s00043g686.1	Gpa3	1	0.39	0.59	31.671875	2	0.23	0.52	31.703125	3	0.21	0.46	31.71875
AOL_s00043g705.1	Gpa1	1	0.23	0.49	31.671875	2	0.22	0.48	31.703125	3	0.17	0.46	31.71875
AOL_s00043g705.1	Gpa2	2	0.24	0.49	31.6875	1	0.33	0.52	31.703125	3	0.21	0.47	31.71875
AOL_s00043g705.1	Gpa3	2	0.22	0.48	31.6875	1	0.26	0.49	31.734375	3	0.19	0.47	31.734375
AOL_s00043g731.1	Gpa1	2	0.62	0.67	31.640625	1	0.62	0.66	31.671875	3	0.58	0.64	31.671875
AOL_s00043g731.1	Gpa2	1	0.43	0.55	31.671875	2	0.37	0.52	31.671875	3	0.24	0.47	31.6875
AOL_s00043g731.1	Gpa3	3	0.37	0.55	31.671875	1	0.5	0.61	31.6875	2	0.43	0.56	31.6875
AOL_s00043g738.2	Gpa1	1	0.65	0.69	31.5625	2	0.62	0.67	31.6875	3	0.59	0.65	31.671875
AOL_s00043g738.2	Gpa2	3	0.37	0.53	31.640625	1	0.44	0.56	31.6875	2	0.43	0.56	31.703125
AOL_s00043g738.2	Gpa3	2	0.41	0.56	31.65625	3	0.36	0.54	31.6875	1	0.43	0.56	31.703125
AOL_s00054g135.1	Gpa1	2	0.69	0.63	31.671875	3	0.41	0.51	31.703125	1	0.69	0.62	31.703125
AOL_s00054g135.1	Gpa2	1	0.32	0.46	31.671875	3	0.26	0.45	31.703125	2	0.31	0.44	31.703125
AOL_s00054g135.1	Gpa3	3	0.24	0.45	31.671875	2	0.29	0.47	31.703125	1	0.3	0.43	31.703125
AOL_s00054g150.1	Gpa1	2	0.66	0.7	31.625	1	0.67	0.69	31.703125	3	0.61	0.65	31.6875
AOL_s00054g150.1	Gpa2	2	0.53	0.61	31.625	1	0.57	0.63	31.703125	3	0.44	0.56	31.6875
AOL_s00054g150.1	Gpa3	1	0.56	0.65	31.609375	2	0.56	0.64	31.703125	3	0.3	0.51	31.703125

Source: af2_all_model_scores_clean.tsv. Original score basenames are reconstructed exactly using the rule in S6H. Global maximum PAE values are reproduced at source precision.

S6I Complete original model scores part 3

All rows use seed 0. One row represents one receptor–Gα combination; W1, W2 and W3 denote the three model weights. Each block gives archived rank, ipTM, pTM and maximum PAE in Å. Together, these 267 rows reproduce all 801 original models without averaging.

Receptor	Partner	W1 Rank	W1 ipTM	W1 pTM	W1 Max PAE Å	W2 Rank	W2 ipTM	W2 pTM	W2 Max PAE Å	W3 Rank	W3 ipTM	W3 pTM	W3 Max PAE Å
AOL_s00054g200.1	Gpa1	2	0.56	0.67	31.640625	1	0.56	0.66	31.65625	3	0.31	0.52	31.703125
AOL_s00054g200.1	Gpa2	3	0.19	0.46	31.703125	1	0.21	0.51	31.71875	2	0.18	0.49	31.71875
AOL_s00054g200.1	Gpa3	2	0.18	0.48	31.671875	1	0.41	0.58	31.6875	3	0.16	0.47	31.71875
AOL_s00054g223.1	Gpa1	2	0.51	0.58	31.640625	1	0.52	0.58	31.71875	3	0.49	0.56	31.703125
AOL_s00054g223.1	Gpa2	2	0.21	0.45	31.671875	1	0.22	0.44	31.703125	3	0.19	0.43	31.71875
AOL_s00054g223.1	Gpa3	2	0.2	0.46	31.671875	1	0.28	0.45	31.71875	3	0.19	0.43	31.703125
AOL_s00054g270.1	Gpa1	3	0.53	0.63	31.5	1	0.56	0.65	31.625	2	0.54	0.63	31.65625
AOL_s00054g270.1	Gpa2	2	0.34	0.52	31.625	1	0.46	0.59	31.640625	3	0.33	0.53	31.6875
AOL_s00054g270.1	Gpa3	3	0.5	0.62	31.609375	2	0.53	0.63	31.640625	1	0.56	0.65	31.65625
AOL_s00054g291.1	Gpa1	2	0.16	0.4	31.703125	1	0.19	0.39	31.71875	3	0.14	0.37	31.71875
AOL_s00054g291.1	Gpa2	1	0.18	0.42	31.703125	2	0.17	0.4	31.734375	3	0.14	0.41	31.734375
AOL_s00054g291.1	Gpa3	2	0.16	0.43	31.703125	1	0.21	0.43	31.71875	3	0.15	0.4	31.734375
AOL_s00054g428.1	Gpa1	3	0.24	0.49	31.703125	1	0.29	0.52	31.703125	2	0.26	0.5	31.71875
AOL_s00054g428.1	Gpa2	3	0.19	0.5	31.703125	1	0.22	0.5	31.71875	2	0.21	0.48	31.71875
AOL_s00054g428.1	Gpa3	2	0.21	0.49	31.703125	1	0.21	0.5	31.703125	3	0.17	0.46	31.71875
AOL_s00054g605.1	Gpa1	2	0.6	0.69	31.578125	1	0.6	0.69	31.65625	3	0.48	0.61	31.6875
AOL_s00054g605.1	Gpa2	2	0.57	0.66	31.640625	1	0.59	0.66	31.65625	3	0.35	0.55	31.671875
AOL_s00054g605.1	Gpa3	2	0.45	0.63	31.671875	1	0.48	0.64	31.671875	3	0.38	0.54	31.71875
AOL_s00054g626.1	Gpa1	1	0.15	0.47	31.671875	3	0.13	0.44	31.703125	2	0.14	0.47	31.6875
AOL_s00054g626.1	Gpa2	3	0.16	0.5	31.703125	2	0.16	0.52	31.703125	1	0.17	0.5	31.703125
AOL_s00054g626.1	Gpa3	1	0.41	0.54	31.65625	2	0.17	0.53	31.703125	3	0.13	0.51	31.703125
AOL_s00054g63.1	Gpa1	1	0.4	0.44	31.703125	3	0.2	0.42	31.703125	2	0.24	0.42	31.703125
AOL_s00054g63.1	Gpa2	3	0.21	0.43	31.703125	2	0.22	0.41	31.71875	1	0.45	0.44	31.71875
AOL_s00054g63.1	Gpa3	3	0.19	0.43	31.71875	2	0.28	0.43	31.734375	1	0.41	0.46	31.703125
AOL_s00054g92.1	Gpa1	1	0.65	0.66	31.703125	2	0.63	0.66	31.703125	3	0.6	0.62	31.703125
AOL_s00054g92.1	Gpa2	1	0.34	0.51	31.671875	2	0.31	0.5	31.6875	3	0.23	0.44	31.71875
AOL_s00054g92.1	Gpa3	2	0.29	0.49	31.671875	1	0.54	0.6	31.6875	3	0.25	0.46	31.71875

Source: af2_all_model_scores_clean.tsv. Original score basenames are reconstructed exactly using the rule in S6H. Global maximum PAE values are reproduced at source precision.

S6I Complete original model scores part 4

All rows use seed 0. One row represents one receptor–Gα combination; W1, W2 and W3 denote the three model weights. Each block gives archived rank, ipTM, pTM and maximum PAE in Å. Together, these 267 rows reproduce all 801 original models without averaging.

Receptor	Partner	W1 Rank	W1 ipTM	W1 pTM	W1 Max PAE Å	W2 Rank	W2 ipTM	W2 pTM	W2 Max PAE Å	W3 Rank	W3 ipTM	W3 pTM	W3 Max PAE Å
AOL_s00075g112.1	Gpa1	3	0.68	0.65	31.671875	1	0.7	0.65	31.71875	2	0.69	0.65	31.6875
AOL_s00075g112.1	Gpa2	1	0.48	0.55	31.6875	2	0.42	0.52	31.71875	3	0.19	0.43	31.71875
AOL_s00075g112.1	Gpa3	3	0.33	0.5	31.6875	2	0.34	0.5	31.71875	1	0.61	0.61	31.6875
AOL_s00075g223.1	Gpa1	1	0.69	0.69	31.671875	2	0.64	0.67	31.6875	3	0.61	0.63	31.71875
AOL_s00075g223.1	Gpa2	2	0.63	0.66	31.6875	1	0.64	0.65	31.6875	3	0.59	0.64	31.703125
AOL_s00075g223.1	Gpa3	2	0.57	0.67	31.640625	1	0.59	0.64	31.6875	3	0.52	0.6	31.703125
AOL_s00076g157.1	Gpa1	3	0.17	0.49	31.71875	2	0.18	0.5	31.703125	1	0.19	0.5	31.71875
AOL_s00076g157.1	Gpa2	3	0.19	0.49	31.71875	1	0.24	0.5	31.71875	2	0.19	0.5	31.71875
AOL_s00076g157.1	Gpa3	2	0.19	0.49	31.71875	3	0.19	0.49	31.71875	1	0.26	0.5	31.703125
AOL_s00076g249.1	Gpa1	2	0.45	0.58	31.640625	3	0.43	0.55	31.671875	1	0.46	0.59	31.703125
AOL_s00076g249.1	Gpa2	2	0.18	0.47	31.671875	1	0.19	0.47	31.671875	3	0.17	0.46	31.703125
AOL_s00076g249.1	Gpa3	3	0.2	0.48	31.640625	2	0.21	0.48	31.6875	1	0.25	0.51	31.6875
AOL_s00076g286.1	Gpa1	1	0.64	0.7	31.609375	2	0.59	0.67	31.671875	3	0.38	0.57	31.703125
AOL_s00076g286.1	Gpa2	1	0.39	0.57	31.640625	2	0.38	0.58	31.6875	3	0.17	0.49	31.734375
AOL_s00076g286.1	Gpa3	3	0.24	0.51	31.65625	1	0.34	0.55	31.703125	2	0.28	0.53	31.703125
AOL_s00076g450.1	Gpa1	1	0.7	0.71	31.640625	2	0.69	0.7	31.671875	3	0.67	0.68	31.6875
AOL_s00076g450.1	Gpa2	2	0.31	0.5	31.625	1	0.34	0.5	31.6875	3	0.26	0.47	31.6875
AOL_s00076g450.1	Gpa3	1	0.65	0.67	31.640625	3	0.6	0.64	31.703125	2	0.62	0.64	31.6875
AOL_s00076g465.1	Gpa1	2	0.6	0.7	31.625	1	0.61	0.7	31.671875	3	0.37	0.58	31.703125
AOL_s00076g465.1	Gpa2	2	0.24	0.53	31.65625	1	0.3	0.56	31.671875	3	0.16	0.49	31.703125
AOL_s00076g465.1	Gpa3	2	0.25	0.53	31.6875	1	0.28	0.55	31.6875	3	0.23	0.53	31.703125
AOL_s00076g508.1	Gpa1	1	0.69	0.71	31.609375	2	0.68	0.7	31.671875	3	0.63	0.65	31.703125
AOL_s00076g508.1	Gpa2	3	0.17	0.46	31.703125	2	0.19	0.48	31.71875	1	0.2	0.48	31.71875
AOL_s00076g508.1	Gpa3	1	0.32	0.55	31.640625	2	0.27	0.52	31.6875	3	0.2	0.46	31.71875
AOL_s00076g553.1	Gpa1	1	0.66	0.58	31.65625	3	0.64	0.58	31.6875	2	0.64	0.58	31.6875
AOL_s00076g553.1	Gpa2	3	0.26	0.42	31.703125	1	0.36	0.44	31.703125	2	0.3	0.44	31.703125
AOL_s00076g553.1	Gpa3	1	0.61	0.56	31.671875	3	0.5	0.5	31.703125	2	0.6	0.54	31.703125

Source: af2_all_model_scores_clean.tsv. Original score basenames are reconstructed exactly using the rule in S6H. Global maximum PAE values are reproduced at source precision.

S6I Complete original model scores part 5

All rows use seed 0. One row represents one receptor–Gα combination; W1, W2 and W3 denote the three model weights. Each block gives archived rank, ipTM, pTM and maximum PAE in Å. Together, these 267 rows reproduce all 801 original models without averaging.

Receptor	Partner	W1 Rank	W1 ipTM	W1 pTM	W1 Max PAE Å	W2 Rank	W2 ipTM	W2 pTM	W2 Max PAE Å	W3 Rank	W3 ipTM	W3 pTM	W3 Max PAE Å
AOL_s00076g86.1	Gpa1	3	0.58	0.65	31.671875	1	0.61	0.66	31.671875	2	0.61	0.65	31.6875
AOL_s00076g86.1	Gpa2	2	0.2	0.46	31.6875	3	0.19	0.45	31.6875	1	0.61	0.65	31.703125
AOL_s00076g86.1	Gpa3	1	0.24	0.5	31.671875	2	0.22	0.51	31.671875	3	0.21	0.46	31.6875
AOL_s00078g446.1	Gpa1	2	0.28	0.53	31.609375	1	0.35	0.54	31.671875	3	0.17	0.47	31.703125
AOL_s00078g446.1	Gpa2	2	0.15	0.48	31.65625	1	0.18	0.5	31.671875	3	0.12	0.47	31.71875
AOL_s00078g446.1	Gpa3	1	0.34	0.56	31.671875	2	0.23	0.54	31.65625	3	0.22	0.49	31.703125
AOL_s00078g563.1	Gpa1	2	0.59	0.61	31.703125	1	0.62	0.6	31.703125	3	0.3	0.44	31.703125
AOL_s00078g563.1	Gpa2	2	0.34	0.51	31.6875	3	0.26	0.47	31.703125	1	0.44	0.53	31.71875
AOL_s00078g563.1	Gpa3	2	0.2	0.47	31.71875	1	0.45	0.57	31.71875	3	0.19	0.47	31.703125
AOL_s00078g598.1	Gpa1	1	0.69	0.64	31.65625	2	0.67	0.64	31.6875	3	0.66	0.62	31.71875
AOL_s00078g598.1	Gpa2	1	0.65	0.66	31.640625	3	0.56	0.53	31.6875	2	0.62	0.62	31.6875
AOL_s00078g598.1	Gpa3	1	0.52	0.6	31.671875	3	0.48	0.56	31.6875	2	0.49	0.57	31.71875
AOL_s00079g307.1	Gpa1	2	0.52	0.5	31.71875	1	0.58	0.54	31.703125	3	0.3	0.43	31.71875
AOL_s00079g307.1	Gpa2	2	0.43	0.47	31.71875	1	0.45	0.48	31.703125	3	0.2	0.39	31.734375
AOL_s00079g307.1	Gpa3	3	0.44	0.46	31.703125	2	0.47	0.5	31.71875	1	0.54	0.51	31.71875
AOL_s00079g452.1	Gpa1	1	0.64	0.61	31.71875	3	0.6	0.58	31.703125	2	0.61	0.59	31.703125
AOL_s00079g452.1	Gpa2	2	0.23	0.45	31.6875	1	0.34	0.45	31.703125	3	0.22	0.44	31.71875
AOL_s00079g452.1	Gpa3	2	0.58	0.58	31.6875	3	0.34	0.5	31.703125	1	0.6	0.58	31.703125
AOL_s00080g10.1	Gpa1	2	0.54	0.65	31.625	1	0.56	0.63	31.671875	3	0.48	0.59	31.703125
AOL_s00080g10.1	Gpa2	2	0.24	0.51	31.671875	1	0.26	0.53	31.671875	3	0.22	0.49	31.703125
AOL_s00080g10.1	Gpa3	3	0.2	0.52	31.625	1	0.38	0.6	31.671875	2	0.38	0.54	31.703125
AOL_s00080g416.1	Gpa1	2	0.57	0.61	31.65625	1	0.59	0.63	31.703125	3	0.52	0.58	31.703125
AOL_s00080g416.1	Gpa2	1	0.43	0.54	31.609375	2	0.38	0.45	31.703125	3	0.33	0.48	31.6875
AOL_s00080g416.1	Gpa3	1	0.46	0.55	31.640625	2	0.44	0.53	31.6875	3	0.37	0.51	31.703125
AOL_s00081g15.1	Gpa1	2	0.61	0.66	31.671875	3	0.6	0.65	31.703125	1	0.62	0.66	31.703125
AOL_s00081g15.1	Gpa2	3	0.42	0.57	31.640625	2	0.45	0.58	31.6875	1	0.48	0.58	31.71875
AOL_s00081g15.1	Gpa3	3	0.23	0.48	31.671875	2	0.26	0.48	31.703125	1	0.31	0.45	31.71875

Source: af2_all_model_scores_clean.tsv. Original score basenames are reconstructed exactly using the rule in S6H. Global maximum PAE values are reproduced at source precision.

S6I Complete original model scores part 6

All rows use seed 0. One row represents one receptor–Gα combination; W1, W2 and W3 denote the three model weights. Each block gives archived rank, ipTM, pTM and maximum PAE in Å. Together, these 267 rows reproduce all 801 original models without averaging.

Receptor	Partner	W1 Rank	W1 ipTM	W1 pTM	W1 Max PAE Å	W2 Rank	W2 ipTM	W2 pTM	W2 Max PAE Å	W3 Rank	W3 ipTM	W3 pTM	W3 Max PAE Å
AOL_s00081g30.1	Gpa1	2	0.25	0.44	31.6875	1	0.42	0.46	31.6875	3	0.25	0.4	31.71875
AOL_s00081g30.1	Gpa2	1	0.22	0.42	31.6875	3	0.2	0.42	31.703125	2	0.22	0.42	31.71875
AOL_s00081g30.1	Gpa3	3	0.24	0.43	31.6875	2	0.29	0.43	31.71875	1	0.42	0.41	31.71875
AOL_s00083g156.1	Gpa1	2	0.65	0.53	31.6875	3	0.64	0.52	31.703125	1	0.68	0.53	31.703125
AOL_s00083g156.1	Gpa2	1	0.36	0.43	31.71875	2	0.3	0.39	31.71875	3	0.2	0.39	31.703125
AOL_s00083g156.1	Gpa3	1	0.62	0.53	31.703125	2	0.6	0.5	31.71875	3	0.58	0.5	31.703125
AOL_s00097g260.1	Gpa1	2	0.52	0.68	31.578125	1	0.55	0.68	31.640625	3	0.24	0.53	31.71875
AOL_s00097g260.1	Gpa2	2	0.21	0.53	31.65625	1	0.23	0.52	31.640625	3	0.17	0.5	31.734375
AOL_s00097g260.1	Gpa3	2	0.21	0.53	31.6875	1	0.25	0.54	31.6875	3	0.17	0.5	31.71875
AOL_s00097g290.1	Gpa1	1	0.67	0.62	31.671875	2	0.65	0.61	31.6875	3	0.62	0.59	31.671875
AOL_s00097g290.1	Gpa2	2	0.21	0.44	31.703125	1	0.21	0.43	31.703125	3	0.21	0.41	31.71875
AOL_s00097g290.1	Gpa3	1	0.34	0.45	31.703125	2	0.31	0.48	31.734375	3	0.23	0.42	31.703125
AOL_s00097g309.3	Gpa1	2	0.62	0.67	31.640625	1	0.62	0.67	31.671875	3	0.59	0.65	31.671875
AOL_s00097g309.3	Gpa2	2	0.34	0.51	31.640625	1	0.45	0.56	31.703125	3	0.21	0.46	31.6875
AOL_s00097g309.3	Gpa3	3	0.33	0.55	31.625	1	0.41	0.58	31.6875	2	0.36	0.54	31.71875
AOL_s00097g558.3	Gpa1	2	0.4	0.57	31.640625	1	0.43	0.57	31.6875	3	0.34	0.52	31.703125
AOL_s00097g558.3	Gpa2	2	0.18	0.48	31.671875	1	0.2	0.48	31.6875	3	0.15	0.47	31.734375
AOL_s00097g558.3	Gpa3	3	0.21	0.49	31.6875	1	0.31	0.54	31.6875	2	0.23	0.49	31.703125
AOL_s00097g69.1	Gpa1	2	0.21	0.49	31.6875	1	0.27	0.5	31.703125	3	0.17	0.42	31.71875
AOL_s00097g69.1	Gpa2	1	0.57	0.62	31.6875	3	0.35	0.51	31.703125	2	0.46	0.57	31.71875
AOL_s00097g69.1	Gpa3	3	0.19	0.49	31.6875	1	0.21	0.49	31.703125	2	0.2	0.44	31.71875
AOL_s00109g63.1	Gpa1	2	0.57	0.68	31.609375	1	0.59	0.68	31.65625	3	0.5	0.63	31.6875
AOL_s00109g63.1	Gpa2	3	0.24	0.52	31.671875	1	0.43	0.59	31.6875	2	0.25	0.53	31.6875
AOL_s00109g63.1	Gpa3	1	0.36	0.56	31.671875	3	0.3	0.54	31.671875	2	0.3	0.53	31.703125
AOL_s00109g8.1	Gpa1	1	0.6	0.65	31.671875	2	0.59	0.64	31.6875	3	0.58	0.62	31.703125
AOL_s00109g8.1	Gpa2	3	0.16	0.45	31.703125	2	0.17	0.44	31.703125	1	0.18	0.44	31.71875
AOL_s00109g8.1	Gpa3	3	0.25	0.48	31.6875	2	0.29	0.51	31.6875	1	0.56	0.62	31.703125

Source: af2_all_model_scores_clean.tsv. Original score basenames are reconstructed exactly using the rule in S6H. Global maximum PAE values are reproduced at source precision.

S6I Complete original model scores part 7

All rows use seed 0. One row represents one receptor–Gα combination; W1, W2 and W3 denote the three model weights. Each block gives archived rank, ipTM, pTM and maximum PAE in Å. Together, these 267 rows reproduce all 801 original models without averaging.

Receptor	Partner	W1 Rank	W1 ipTM	W1 pTM	W1 Max PAE Å	W2 Rank	W2 ipTM	W2 pTM	W2 Max PAE Å	W3 Rank	W3 ipTM	W3 pTM	W3 Max PAE Å
AOL_s00110g16.1	Gpa1	1	0.59	0.69	31.609375	2	0.59	0.68	31.6875	3	0.48	0.62	31.6875
AOL_s00110g16.1	Gpa2	2	0.37	0.56	31.65625	1	0.38	0.56	31.671875	3	0.17	0.49	31.71875
AOL_s00110g16.1	Gpa3	2	0.25	0.53	31.6875	1	0.38	0.59	31.6875	3	0.16	0.48	31.703125
AOL_s00110g307.1	Gpa1	1	0.67	0.69	31.59375	2	0.65	0.67	31.65625	3	0.64	0.66	31.6875
AOL_s00110g307.1	Gpa2	2	0.61	0.65	31.625	3	0.6	0.64	31.640625	1	0.64	0.66	31.703125
AOL_s00110g307.1	Gpa3	2	0.62	0.65	31.640625	1	0.62	0.65	31.671875	3	0.59	0.64	31.6875
AOL_s00110g337.1	Gpa1	3	0.46	0.58	31.625	1	0.55	0.63	31.6875	2	0.52	0.62	31.6875
AOL_s00110g337.1	Gpa2	3	0.19	0.46	31.671875	1	0.45	0.59	31.703125	2	0.26	0.5	31.6875
AOL_s00110g337.1	Gpa3	3	0.22	0.47	31.6875	1	0.29	0.51	31.703125	2	0.22	0.48	31.71875
AOL_s00140g54.6	Gpa1	2	0.64	0.68	31.65625	1	0.66	0.69	31.671875	3	0.63	0.67	31.6875
AOL_s00140g54.6	Gpa2	3	0.19	0.47	31.65625	1	0.26	0.48	31.703125	2	0.26	0.47	31.703125
AOL_s00140g54.6	Gpa3	1	0.59	0.66	31.640625	2	0.58	0.64	31.703125	3	0.57	0.63	31.703125
AOL_s00169g110.1	Gpa1	2	0.62	0.7	31.609375	1	0.63	0.7	31.671875	3	0.6	0.68	31.6875
AOL_s00169g110.1	Gpa2	2	0.35	0.54	31.65625	1	0.44	0.6	31.671875	3	0.14	0.47	31.703125
AOL_s00169g110.1	Gpa3	1	0.46	0.56	31.6875	2	0.34	0.52	31.703125	3	0.17	0.48	31.6875
AOL_s00169g261.1	Gpa1	2	0.67	0.71	31.609375	1	0.68	0.72	31.6875	3	0.65	0.68	31.640625
AOL_s00169g261.1	Gpa2	2	0.28	0.49	31.671875	1	0.37	0.54	31.6875	3	0.18	0.47	31.703125
AOL_s00169g261.1	Gpa3	1	0.58	0.67	31.609375	2	0.58	0.65	31.6875	3	0.56	0.64	31.6875
AOL_s00170g20.1	Gpa1	2	0.52	0.54	31.671875	1	0.57	0.57	31.703125	3	0.48	0.52	31.6875
AOL_s00170g20.1	Gpa2	2	0.32	0.43	31.71875	1	0.37	0.49	31.703125	3	0.24	0.41	31.71875
AOL_s00170g20.1	Gpa3	2	0.2	0.41	31.703125	1	0.26	0.44	31.71875	3	0.16	0.39	31.71875
AOL_s00170g54.1	Gpa1	2	0.64	0.65	31.6875	3	0.64	0.64	31.71875	1	0.65	0.65	31.703125
AOL_s00170g54.1	Gpa2	1	0.38	0.49	31.671875	3	0.3	0.48	31.703125	2	0.35	0.49	31.703125
AOL_s00170g54.1	Gpa3	2	0.37	0.5	31.6875	3	0.3	0.5	31.703125	1	0.47	0.56	31.703125
AOL_s00170g99.1	Gpa1	1	0.57	0.63	31.59375	3	0.44	0.56	31.6875	2	0.45	0.56	31.671875
AOL_s00170g99.1	Gpa2	1	0.4	0.53	31.609375	3	0.27	0.49	31.6875	2	0.27	0.49	31.6875
AOL_s00170g99.1	Gpa3	3	0.23	0.51	31.65625	1	0.34	0.52	31.703125	2	0.26	0.48	31.703125

Source: af2_all_model_scores_clean.tsv. Original score basenames are reconstructed exactly using the rule in S6H. Global maximum PAE values are reproduced at source precision.

S6I Complete original model scores part 8

All rows use seed 0. One row represents one receptor–Gα combination; W1, W2 and W3 denote the three model weights. Each block gives archived rank, ipTM, pTM and maximum PAE in Å. Together, these 267 rows reproduce all 801 original models without averaging.

Receptor	Partner	W1 Rank	W1 ipTM	W1 pTM	W1 Max PAE Å	W2 Rank	W2 ipTM	W2 pTM	W2 Max PAE Å	W3 Rank	W3 ipTM	W3 pTM	W3 Max PAE Å
AOL_s00173g137.1	Gpa1	2	0.65	0.69	31.609375	1	0.65	0.69	31.6875	3	0.64	0.68	31.671875
AOL_s00173g137.1	Gpa2	2	0.21	0.47	31.65625	1	0.32	0.5	31.671875	3	0.21	0.46	31.71875
AOL_s00173g137.1	Gpa3	2	0.26	0.52	31.640625	1	0.37	0.57	31.65625	3	0.2	0.48	31.671875
AOL_s00173g258.2	Gpa1	2	0.47	0.61	31.640625	1	0.5	0.59	31.671875	3	0.42	0.53	31.703125
AOL_s00173g258.2	Gpa2	3	0.49	0.58	31.625	1	0.6	0.63	31.671875	2	0.56	0.59	31.6875
AOL_s00173g258.2	Gpa3	3	0.48	0.59	31.703125	1	0.52	0.58	31.703125	2	0.49	0.56	31.703125
AOL_s00173g37.1	Gpa1	2	0.67	0.65	31.671875	1	0.68	0.65	31.703125	3	0.65	0.64	31.6875
AOL_s00173g37.1	Gpa2	3	0.23	0.45	31.703125	1	0.29	0.46	31.703125	2	0.28	0.45	31.6875
AOL_s00173g37.1	Gpa3	2	0.55	0.59	31.671875	1	0.57	0.59	31.703125	3	0.52	0.57	31.6875
AOL_s00176g24.1	Gpa1	2	0.54	0.64	31.640625	1	0.58	0.65	31.703125	3	0.14	0.45	31.703125
AOL_s00176g24.1	Gpa2	1	0.26	0.52	31.671875	2	0.25	0.5	31.703125	3	0.19	0.48	31.71875
AOL_s00176g24.1	Gpa3	2	0.27	0.52	31.6875	1	0.31	0.53	31.71875	3	0.16	0.45	31.703125
AOL_s00176g42.1	Gpa1	2	0.35	0.51	31.65625	1	0.37	0.5	31.6875	3	0.29	0.46	31.6875
AOL_s00176g42.1	Gpa2	3	0.2	0.44	31.671875	2	0.23	0.42	31.703125	1	0.24	0.45	31.71875
AOL_s00176g42.1	Gpa3	3	0.21	0.47	31.671875	1	0.23	0.47	31.703125	2	0.22	0.44	31.703125
AOL_s00188g124.1	Gpa1	3	0.18	0.52	31.671875	1	0.2	0.53	31.703125	2	0.2	0.52	31.703125
AOL_s00188g124.1	Gpa2	2	0.17	0.51	31.6875	1	0.18	0.52	31.71875	3	0.15	0.5	31.6875
AOL_s00188g124.1	Gpa3	2	0.16	0.51	31.6875	1	0.17	0.51	31.703125	3	0.15	0.51	31.703125
AOL_s00188g142.1	Gpa1	3	0.7	0.5	31.71875	1	0.73	0.51	31.703125	2	0.7	0.51	31.703125
AOL_s00188g142.1	Gpa2	3	0.61	0.48	31.703125	1	0.64	0.5	31.703125	2	0.65	0.4	31.703125
AOL_s00188g142.1	Gpa3	1	0.65	0.49	31.703125	2	0.62	0.48	31.703125	3	0.53	0.44	31.703125
AOL_s00188g284.1	Gpa1	1	0.66	0.66	31.65625	2	0.66	0.65	31.6875	3	0.62	0.63	31.6875
AOL_s00188g284.1	Gpa2	3	0.19	0.47	31.65625	2	0.37	0.5	31.671875	1	0.44	0.47	31.703125
AOL_s00188g284.1	Gpa3	2	0.54	0.63	31.609375	1	0.55	0.6	31.671875	3	0.38	0.52	31.6875
AOL_s00188g320.1	Gpa1	2	0.54	0.64	31.65625	1	0.55	0.63	31.703125	3	0.15	0.46	31.734375
AOL_s00188g320.1	Gpa2	1	0.24	0.48	31.65625	2	0.22	0.47	31.71875	3	0.21	0.49	31.71875
AOL_s00188g320.1	Gpa3	2	0.31	0.52	31.6875	1	0.33	0.53	31.703125	3	0.12	0.44	31.703125

Source: af2_all_model_scores_clean.tsv. Original score basenames are reconstructed exactly using the rule in S6H. Global maximum PAE values are reproduced at source precision.

S6I Complete original model scores part 9

All rows use seed 0. One row represents one receptor–Gα combination; W1, W2 and W3 denote the three model weights. Each block gives archived rank, ipTM, pTM and maximum PAE in Å. Together, these 267 rows reproduce all 801 original models without averaging.

Receptor	Partner	W1 Rank	W1 ipTM	W1 pTM	W1 Max PAE Å	W2 Rank	W2 ipTM	W2 pTM	W2 Max PAE Å	W3 Rank	W3 ipTM	W3 pTM	W3 Max PAE Å
AOL_s00188g342.1	Gpa1	1	0.63	0.67	31.640625	3	0.6	0.64	31.6875	2	0.6	0.65	31.6875
AOL_s00188g342.1	Gpa2	3	0.24	0.47	31.65625	2	0.33	0.5	31.6875	1	0.46	0.56	31.6875
AOL_s00188g342.1	Gpa3	2	0.32	0.52	31.65625	1	0.36	0.53	31.703125	3	0.33	0.5	31.703125
AOL_s00188g346.1	Gpa1	2	0.5	0.59	31.6875	1	0.51	0.59	31.71875	3	0.21	0.43	31.71875
AOL_s00188g346.1	Gpa2	3	0.2	0.45	31.6875	1	0.26	0.46	31.703125	2	0.21	0.45	31.71875
AOL_s00188g346.1	Gpa3	2	0.23	0.49	31.703125	1	0.26	0.5	31.703125	3	0.22	0.45	31.734375
AOL_s00193g107.1	Gpa1	2	0.64	0.68	31.609375	3	0.64	0.67	31.71875	1	0.67	0.69	31.71875
AOL_s00193g107.1	Gpa2	2	0.45	0.55	31.609375	1	0.49	0.58	31.703125	3	0.32	0.51	31.71875
AOL_s00193g107.1	Gpa3	3	0.6	0.66	31.703125	2	0.61	0.66	31.703125	1	0.63	0.66	31.6875
AOL_s00210g135.2	Gpa1	1	0.62	0.68	31.53125	3	0.6	0.64	31.640625	2	0.61	0.65	31.671875
AOL_s00210g135.2	Gpa2	1	0.5	0.6	31.6875	3	0.32	0.52	31.71875	2	0.43	0.57	31.71875
AOL_s00210g135.2	Gpa3	3	0.24	0.52	31.640625	1	0.51	0.61	31.625	2	0.37	0.54	31.671875
AOL_s00210g301.1	Gpa1	3	0.68	0.67	31.671875	2	0.69	0.66	31.6875	1	0.69	0.67	31.703125
AOL_s00210g301.1	Gpa2	3	0.21	0.44	31.6875	2	0.22	0.45	31.703125	1	0.23	0.45	31.703125
AOL_s00210g301.1	Gpa3	2	0.52	0.47	31.6875	1	0.6	0.62	31.6875	3	0.3	0.47	31.703125
AOL_s00210g381.1	Gpa1	1	0.59	0.66	31.703125	3	0.15	0.48	31.71875	2	0.2	0.49	31.71875
AOL_s00210g381.1	Gpa2	1	0.24	0.5	31.6875	2	0.24	0.49	31.71875	3	0.17	0.46	31.71875
AOL_s00210g381.1	Gpa3	3	0.17	0.48	31.6875	2	0.18	0.49	31.71875	1	0.19	0.47	31.71875
AOL_s00210g385.1	Gpa1	3	0.58	0.64	31.640625	1	0.59	0.64	31.671875	2	0.59	0.62	31.703125
AOL_s00210g385.1	Gpa2	2	0.33	0.5	31.65625	1	0.38	0.52	31.671875	3	0.25	0.46	31.6875
AOL_s00210g385.1	Gpa3	2	0.26	0.51	31.671875	1	0.34	0.51	31.71875	3	0.23	0.47	31.703125
AOL_s00215g252.1	Gpa1	3	0.41	0.54	31.65625	1	0.5	0.58	31.703125	2	0.48	0.57	31.703125
AOL_s00215g252.1	Gpa2	2	0.22	0.44	31.6875	1	0.33	0.49	31.71875	3	0.2	0.43	31.71875
AOL_s00215g252.1	Gpa3	3	0.19	0.43	31.6875	1	0.27	0.47	31.734375	2	0.2	0.44	31.71875
AOL_s00215g301.1	Gpa1	2	0.58	0.62	31.640625	1	0.62	0.63	31.6875	3	0.56	0.6	31.703125
AOL_s00215g301.1	Gpa2	1	0.2	0.45	31.6875	3	0.19	0.44	31.703125	2	0.19	0.44	31.71875
AOL_s00215g301.1	Gpa3	2	0.24	0.46	31.6875	3	0.2	0.44	31.703125	1	0.33	0.49	31.6875

Source: af2_all_model_scores_clean.tsv. Original score basenames are reconstructed exactly using the rule in S6H. Global maximum PAE values are reproduced at source precision.

S6I Complete original model scores part 10

All rows use seed 0. One row represents one receptor–Gα combination; W1, W2 and W3 denote the three model weights. Each block gives archived rank, ipTM, pTM and maximum PAE in Å. Together, these 267 rows reproduce all 801 original models without averaging.

Receptor	Partner	W1 Rank	W1 ipTM	W1 pTM	W1 Max PAE Å	W2 Rank	W2 ipTM	W2 pTM	W2 Max PAE Å	W3 Rank	W3 ipTM	W3 pTM	W3 Max PAE Å
AOL_s00215g417.1	Gpa1	2	0.51	0.67	31.625	1	0.52	0.67	31.640625	3	0.15	0.49	31.671875
AOL_s00215g417.1	Gpa2	1	0.3	0.55	31.671875	2	0.25	0.53	31.65625	3	0.17	0.51	31.734375
AOL_s00215g417.1	Gpa3	2	0.2	0.52	31.6875	1	0.26	0.55	31.671875	3	0.13	0.49	31.71875
AOL_s00215g444.1	Gpa1	1	0.64	0.68	31.640625	2	0.63	0.67	31.6875	3	0.62	0.64	31.6875
AOL_s00215g444.1	Gpa2	3	0.17	0.44	31.6875	2	0.45	0.56	31.6875	1	0.56	0.62	31.6875
AOL_s00215g444.1	Gpa3	2	0.54	0.62	31.65625	3	0.3	0.51	31.6875	1	0.54	0.61	31.6875
AOL_s00215g562.1	Gpa1	1	0.39	0.55	31.625	2	0.31	0.49	31.6875	3	0.3	0.49	31.703125
AOL_s00215g562.1	Gpa2	3	0.17	0.44	31.671875	2	0.18	0.43	31.703125	1	0.23	0.47	31.703125
AOL_s00215g562.1	Gpa3	2	0.4	0.46	31.703125	3	0.12	0.43	31.703125	1	0.4	0.53	31.703125
AOL_s00215g603.1	Gpa1	2	0.58	0.64	31.6875	1	0.62	0.67	31.71875	3	0.54	0.63	31.703125
AOL_s00215g603.1	Gpa2	1	0.51	0.59	31.671875	3	0.23	0.5	31.703125	2	0.5	0.6	31.703125
AOL_s00215g603.1	Gpa3	1	0.45	0.6	31.671875	3	0.19	0.48	31.703125	2	0.42	0.6	31.703125
AOL_s00215g796.1	Gpa1	3	0.6	0.62	31.640625	1	0.62	0.64	31.6875	2	0.62	0.64	31.703125
AOL_s00215g796.1	Gpa2	2	0.2	0.44	31.703125	1	0.3	0.46	31.703125	3	0.2	0.43	31.703125
AOL_s00215g796.1	Gpa3	2	0.48	0.56	31.65625	3	0.37	0.52	31.703125	1	0.49	0.55	31.703125
AOL_s00215g906.3	Gpa1	2	0.6	0.62	31.703125	1	0.64	0.63	31.703125	3	0.59	0.61	31.703125
AOL_s00215g906.3	Gpa2	3	0.18	0.44	31.6875	1	0.39	0.51	31.71875	2	0.23	0.43	31.703125
AOL_s00215g906.3	Gpa3	3	0.47	0.56	31.6875	1	0.54	0.59	31.703125	2	0.51	0.56	31.6875
AOL_s00215g915.1	Gpa1	3	0.28	0.53	31.65625	1	0.46	0.61	31.65625	2	0.34	0.55	31.703125
AOL_s00215g915.1	Gpa2	2	0.19	0.48	31.6875	1	0.27	0.53	31.703125	3	0.11	0.46	31.6875
AOL_s00215g915.1	Gpa3	2	0.2	0.48	31.703125	1	0.26	0.52	31.703125	3	0.17	0.48	31.6875
AOL_s00215g924.1	Gpa1	2	0.49	0.65	31.625	1	0.52	0.64	31.671875	3	0.36	0.56	31.6875
AOL_s00215g924.1	Gpa2	2	0.2	0.5	31.6875	1	0.24	0.51	31.6875	3	0.13	0.47	31.71875
AOL_s00215g924.1	Gpa3	3	0.24	0.52	31.671875	1	0.31	0.54	31.671875	2	0.24	0.53	31.6875

Source: af2_all_model_scores_clean.tsv. Original score basenames are reconstructed exactly using the rule in S6H. Global maximum PAE values are reproduced at source precision.
